# A eukaryotic nicotinate-inducible gene cluster: convergent evolution in fungi and bacteria

**DOI:** 10.1098/rsob.170199

**Published:** 2017-12-06

**Authors:** Judit Ámon, Rafael Fernández-Martín, Eszter Bokor, Antonietta Cultrone, Joan M. Kelly, Michel Flipphi, Claudio Scazzocchio, Zsuzsanna Hamari

**Affiliations:** 1 Department of Microbiology, University of Szeged Faculty of Science and Informatics, Szeged, Hungary (present address of ZH); 2 Institute de Génétique et Microbiologie, Université Paris-Sud, Orsay, France; 3 Department of Biology, University of Essex, Colchester, UK; 4 Department of Microbiology, Imperial College, London, UK (present address of CS); 5 Institute for Integrative Biology of the Cell (I2BC), Gif-sur-Yvette, France (present address of CS)

**Keywords:** nicotinate catabolic gene cluster, convergent evolution, nicotinate hydroxylase, xanthine dehydrogenase, Cys2His2 transcription factor

## Abstract

Nicotinate degradation has hitherto been elucidated only in bacteria. In the ascomycete *Aspergillus nidulans*, six loci, *hxnS*/AN9178 encoding the molybdenum cofactor-containing nicotinate hydroxylase, AN11197 encoding a Cys2/His2 zinc finger regulator HxnR, together with AN11196/*hxnZ*, AN11188/*hxnY*, AN11189/*hxnP* and AN9177/*hxnT*, are clustered and stringently co-induced by a nicotinate derivative and subject to nitrogen metabolite repression mediated by the GATA factor AreA. These genes are strictly co-regulated by HxnR. Within the *hxnR* gene, constitutive mutations map in two discrete regions. *Aspergillus nidulans* is capable of using nicotinate and its oxidation products 6-hydroxynicotinic acid and 2,5-dihydroxypyridine as sole nitrogen sources in an HxnR-dependent way. HxnS is highly similar to HxA, the canonical xanthine dehydrogenase (XDH), and has originated by gene duplication, preceding the origin of the Pezizomycotina. This cluster is conserved with some variations throughout the Aspergillaceae. Our results imply that a fungal pathway has arisen independently from bacterial ones. Significantly, the neo-functionalization of XDH into nicotinate hydroxylase has occurred independently from analogous events in bacteria. This work describes for the first time a gene cluster involved in nicotinate catabolism in a eukaryote and has relevance for the formation and evolution of co-regulated primary metabolic gene clusters and the microbial degradation of *N*-heterocyclic compounds.

## 1. Introduction

Filamentous ascomycetes comprise metabolically versatile saprophytes that can use a large variety of metabolites as nitrogen and/or carbon sources. The utilization of nicotinic acid has been studied in bacteria, but it has only been addressed in a eukaryotic microorganism by our early work in *Aspergillus nidulans*. An enzyme of the xanthine dehydrogenase (XDH) group [1–3] is necessary for this process. Strains mutant in the *cnx* (*cnxABC*, *cnxE*, *cnxF*, *cnxG* and *cnxH*) or *hxB* genes cannot use nicotinate. The *cnx* genes are required for the synthesis of the molybdenum cofactor (MOCO) common to XDH and nitrate reductase [4,5]. The HxB protein catalyses the sulfuration of the Mo(VI), essential for the activity of the enzymes of the XDH group [5,6].

Two enzymes of the XDH family have been described in *A. nidulans*. Purine hydroxylase I (PHI, HxA encoded by the *hxA* gene) is a typical XDH [7–9]. Purine hydroxylase II (PHII, HxnS; see below) has unprecedented substrate specificity. Hypoxanthine, but not xanthine, serves as a substrate of PHII. It accepts nicotinate as a substrate and catalyses the first step of nicotinate catabolism [1,7,10]. Table 1 presents some kinetic parameters for PHI (HxA) and PHII (HxnS) summarized from the relevant literature.

**Table 1.** A summary of the properties of PHI and PHII compiled from the literature. Data from Lewis *et al.* [7] for the properties of the enzymes in crude extracts and from Mehra and Coughland [8] (PHI) and [11] (PHII) for the purified enzymes. The reader is referred to the original articles for further details. R. rate, relative rate to hypoxanthine, given an arbitrary value of 1. The concentration of each substrate was 2.5 times its *Km*.

	PHI (HxA)				PHII (HxnS)			
	crude extract		pur. enzyme		crude extract		pur. enzyme	
substrate	R. rate	*Km* (µM)	R. rate	*Km* (µM)	R. rate	*Km* (µM)	R. rate	*Km* (µM)
hypoxanthine (6-hydroxypurine)	1.00	51.2	1.00	16.4	1.00	90.4	1.00	116
xanthine (2,6-dihydroxypurine)	0.63	161.9	0.61	34.2	—	350^a^	<0.02	—
2-hydroxypurine	0.59	28.3	0.49	16.8	0.38	36.2	0.42	37
allopurinol (4-hydroxypyrazolo-[3,4-d]pyrimidine)	<0.005	—	0.007	—	0.006	0.5	0.007	1^a^
nicotinate	—	—	—	—	0.16	189	0.22	64

^a^*Ki*s of competitive inhibitors with hypoxanthine as a substrate.

PHII is absent in mycelia grown on nitrogen sources generally considered non-repressive. It is apparently induced by nicotinate but it is also present in nitrogen-starved mycelia [1]. The physiological inducer is either 6-OH nicotinate and/or a metabolite further along the nicotinate utilization pathway [12]. The expression of PHII is not under the control of UaY, the transcription factor specific for the expression of the genes in the purine utilization pathway including *hxA* [13–15].

**Figure 1.**
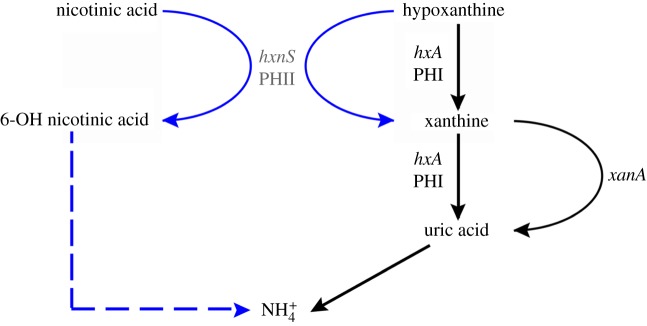
Metabolic cross-talk between the purine and nicotinate utilization pathways. PHI is a conventional XDH able to catalyse the conversion of hypoxanthine to xanthine and xanthine to uric acid. XanA is an α-ketoglutarate-dependent xanthine dioxygenase, accepting xanthine but not hypoxanthine as a substrate. From there uric acid is converted into ammonium (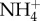) by the well-established purine utilization pathway ([21] for review). PHII is an unconventional MOCO carrying enzyme hydroxylating hypoxanthine to xanthine and nicotinic acid to presumably 6-OH nicotinic acid. As this latter compound is a nitrogen source, it is presumably converted into ammonium, which is indicated by a dashed blue connector. Note that unlike PHI, PHII cannot use xanthine as a substrate. In black: steps induced by uric acid, under the control of the UaY transcription factor. In blue: steps actually (*hxnS*, PHII) or presumably induced by nicotinic acid, 6-OH nicotinic acid or a further metabolite in the nicotinate utilization pathway and under the control of the HxnR/AplA transcription factor(s). Full references are given in the text.

Concentrations of nicotinate below those that can serve as sole nitrogen sources allow hypoxanthine utilization by *hxA*^−^ strains [16,17]. Nicotinate induces PHII, which catalyses the hydroxylation of hypoxanthine to xanthine. Xanthine is further hydroxylated to uric acid by a xanthine dioxygenase encoded by the *xanA* gene [18–20]. This is schematized in figure 1. The induction pattern implies that PHII belongs physiologically to the nicotinate utilization pathway and not to the purine utilization pathway.

In the 1970s and 1980s, we attempted to characterize genetically the nicotinate utilization pathway in *A. nidulans*. The results have only been published schematically [1–3,22] and thus will be summarized below. We isolated mutants able to grow on hypoxanthine as a nitrogen source, but not on a medium that contains hypoxanthine, allopurinol and nicotinate (1 mM), which, at this concentration, does not serve as a nitrogen source but fully induces PHII [1]. The wild-type grows on this medium, as PHII (resistant to allopurinol inhibition [1,7]) hydroxylates hypoxanthine to xanthine, which is further hydroxylated to uric acid by the XanA protein (figure 1). Three groups of mutations, mapping in three different genes, were obtained. One group, *hxnS,* results in the inability to grow on the isolation medium and on nicotinic acid as the sole nitrogen source (10 mM) but maintains its ability to grow on 6-OH nicotinate. These mutations define the structural gene for PHII. Non-leaky *hxnS* mutations resulted in the loss of PHII enzyme activity but were heterogeneous regarding PHII cross-reacting material (CRM) [2,22]. Furthermore, mutations in *hxnR* result also in the complete inability to grow on 6-OH nicotinate. *hxnR* mutants are non-inducible for PHII activity or CRM [22]. The *hxnR* mutations are fully recessive and thus represent loss-of-function mutations. They define an activating transcription factor, necessary for the expression of *hxnS* and at least one other enzyme of the nicotinate utilization pathway, involved in the downstream conversion of 6-OH nicotinate. A number of mutants constitutive for PHII were called *aplA^c^* [1]. These represent regulatory gain-of-function mutations [1]. The *aplA* and *hxnR* mutations could represent two tightly linked genes or a single gene where the relatively frequent constitutive mutations define (a) negative-acting domain(s). The *hxnS*, *hxnR* and *aplA* mutations are tightly linked on chromosome VI (less than 1 centiMorgan for crosses involving several alleles of the three classes). One mutation isolated, described elsewhere, defines the *xanA* gene [18,19].

We report here that *hxnS* and *hxnR* are part of an extended gene cluster that includes four additional co-regulated genes. The *aplA^c^* mutations map in specific domains of the *hxnR* gene product. We discuss the evolutionary relationships between the structurally similar but functionally distinct HxA and HxnS paralogues, the domain structure of HxnR and the conservation of the nicotinate gene cluster in the Aspergillaceae.

## 2. Results

### 2.1. Identification and characterization of the *hxnS* gene

**Figure 2.**
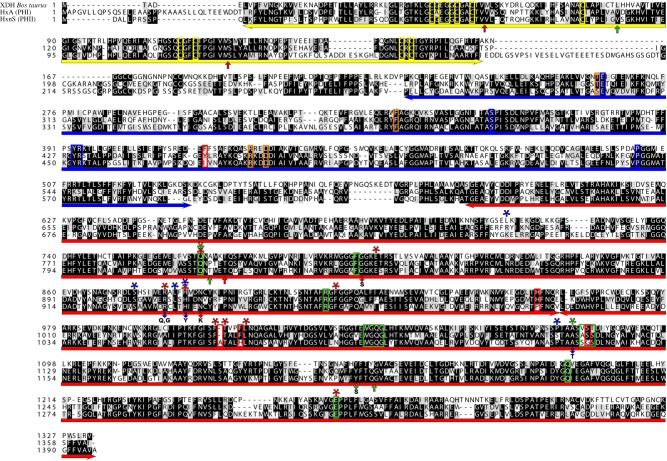
A comparison of PHI (HxA) and PHII (HxnS). An alignment of the two *A. nidulans* open reading frames with the structurally characterized XDH from *B. taurus* [24] is shown. Underlying the sequences: yellow, 2Fe/2S clusters; blue, FAD/NAD-binding domain; red, MOCO/substrate-binding subdomains I and II (as in [25]). Red arrows underlying the sequences indicate intron positions in the *hxA* gene, while green arrows indicate intron positions in *hxnS*. Boxed residues: yellow, conserved Cys in the 2Fe/2S clusters, also indicated the Glu45 and Gly46 (in *B. taurus*) residues belonging to the 2Fe/2S-binding loop, and separating this cluster from the flavin-binding ring; orange, FAD-binding residues [24]; blue, NAD+/NADH-interacting residues [26]; green, residues interacting with MOCO [25]; red, residues where HxnS and its putative orthologues differ from both HxA and typical XDHs represented by the *B. taurus* enzyme. Red asterisks mark residues involved in substrate binding of *B. taurus* XDH [24,27,28]. Blue asterisks mark residues lining the substrate access channel of *B. taurus* XDH [28]. Green asterisks mark residues hydrogen-bonding a molybdenum-bound oxygen [27]. Red downward arrows indicate mutational changes leading to complete loss of function in HxA; blue downward arrows indicate mutations leading to changes of substrate and inhibitor specificity in HxA [29]; the downward green arrow indicates the only extant missense mutation sequenced for HxnS. Alignment with MAFFT E-INS-i, visualized with BoxShade.

**Figure 3.**
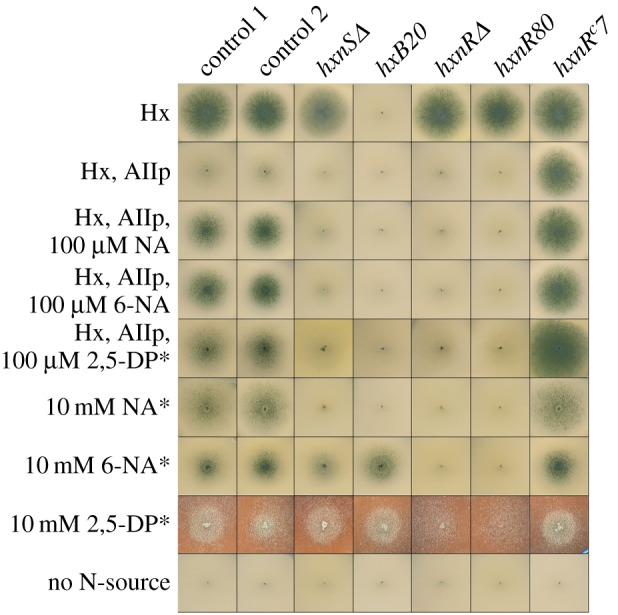
Utilization of different nitrogen sources by mutants described in this article. Above each column we indicate the relevant mutation carried by each tested strain. Hx indicates 1 mM hypoxanthine as the sole nitrogen source. Hx, Allp, as above including 5.5 µM allopurinol, which fully inhibits PHI (HxA) but not PHII (HxnS). NA, 6-NA and 2,5-DP indicate, respectively, nicotinic acid and 6-OH nicotinic acid added as the sodium salts (see ‘Material and methods’ section) and 2,5-dihydroxypyridine added as powder. Other relevant concentrations are indicated in the figure. Plates were incubated for 3 days at 37°C except those marked by asterisk (*), which were incubated for 4 days. Strains used: control 1 (HZS.120, parent of *hxnSΔ*), control 2 (TN02 A21) are wt for all *hxn* genes. Mutant strains: *hxnSΔ* (HZS.599), *hxB20* (HZS.135), *hxnRΔ* (HZS.136), *hxnR80* (HZS.220) and *hxnR^c^7* (FGSC A872). The complete genotypes are given in the electronic supplementary material, table S5.

We expected the *hxnS* gene to be a paralogue of *hxA* [7,16]. In the *A. nidulans* genomic sequences of the Cereon Aspergillus Sequencing Project (later incorporated into the Aspergillus Genome Database, AspGD [23]), we found an incomplete homologue of *hxA* [9]. We localized the sequence encoding this XDH paralogue to chromosome VI cosmid W31:H08 (see ‘Material and methods’ section), in line with the mapping of *hxnS*. This cosmid complements both the *hxnS41* and the *hxnR2* loss-of-function mutations. We sequenced the region comprising the putative *hxnS* gene to reveal a protein with very high (51%) identity to PHI encoded by the *hxA* gene and identical with the protein specified by the AN9178 locus in the AspGD genome database (GenBank accession number KY962010). The cognate full-length cDNA sequence was also obtained (GenBank accession number KX585438). The *hxnS* open reading frame is interrupted by three introns in different positions to those extant in *hxA* (figure 2). The *hxnS* gene encodes a protein of 1396 residues (HxA, 1363 residues). The molecular masses are compatible with those experimentally determined for PHI and PHII native dimers [7] and with the slower migration of HxnS seen in the electronic supplementary material, figure S1, in native polyacrylamide gels. We deleted the putative *hxnS* gene (see ‘Material and methods’ section). The deletion strain is able to grow on hypoxanthine, unable to use nicotinate as a nitrogen source and unable to grow on media containing hypoxanthine (N-source), allopurinol (inhibitor of PHI) and 100 µM nicotinate or 6-OH nicotinate (as inducer), which requires HxnS activity (figure 3; electronic supplementary material, figure S1, the latter showing enzyme activities with both hypoxanthine and nicotinic acid as substrates in native gels). *hxnS*Δ strains are able to use 6-OH nicotinic acid as a nitrogen source, albeit at a reduced level (figure 3; electronic supplementary material, figure S1). The significance of the latter is not clear as the cognate parent strain also uses 6-OH nicotinate badly, and an *hxB20* strain (see sections ‘Introduction’ and ‘A tightly co-regulated gene cluster in chromosome VI’, for HxB function) does not seem to be impaired in its utilization (figure 3). Previously isolated *hxnS* mutations result in the same phenotype as *hxnSΔ* on the N-source hypoxanthine supplemented with allopurinol or on nicotinate. However, they do not show any impairment in 6-OH nicotinate utilization (electronic supplementary material, figure S1). The three classical loss-of-function mutations available were all isolated in an *hxnR*^c^7 background, which results in overexpression of other genes under HxnR control (see below) encoding other proteins putatively involved in 6-OH nicotinate utilization (figure 6*a,b*). The *hxnS35* and *hxnS41* alleles are nonsense mutations (electronic supplementary material, figure S1 shows the corresponding mutational changes), while *hxnS29* results in a Phe1213Ser change in a conserved region (figure 2). The *hxnS35* and *hxnS41* mutations result in loss of PHII CRM, as assessed by immunoprecipitation, while *hxnS29*, a leaky mutation on allopurinol supplemented hypoxanthine medium (see electronic supplementary material, figure S1), fully retains CRM [22]. The above constitutes formal evidence that the locus AN9178 specifies the *hxnS* gene. Strains carrying the *hxnS29* mutation have a clear phenotype *in vivo*, despite showing HxnS activity *in vitro* (electronic supplementary material, figure S1). The Phe1213Ser mutation may affect the stability rather than the activity of the enzyme. We have checked if 6-OH nicotinate (i.e. the product of nicotinate hydroxylase activity) could also be a substrate for HxnS. A very faint staining can be seen after 48 h incubation, a signal not stronger than the one obtained in the absence of substrate, incubating the gel in the presence of the tetrazolium salt (not shown).

### 2.2. A comparison of HxnS (PHII) with HxA (PHI)

Figure 2 compares PHI (HxA) and PHII (HxnS) to the thoroughly chemically and structurally characterized *Bos taurus* XDH enzyme [24,27]. HxnS and HxA and their fungal orthologues (see below) differ less from each other than other eukaryotic XDH paralogues, such as so-called ‘aldehyde oxidases’ from genuine XDHs. Eukaryotic ‘aldehyde oxidases’, so denominated for historical reasons, are enzymes very similar to XDH, but with different substrate specificities [30,31]. Features that differentiate HxnS from HxA and those that are conserved in HxA and HxnS putative fungal orthologues are discussed below.

The residues involved in the two amino-terminal 2Fe/2S clusters, and the FAD- and NAD-binding residues identified in the crystal structure of the *B. taurus* enzyme are strictly conserved in HxA and HxnS (figure 2). HxnS comprises several insertions when compared with HxA and other characterized XDHs (figure 2). The first insertion occurs between the second and the third Cys residues of the second 2Fe/2S cluster. The sequence between the 2Fe/2S cluster domain and the FAD/NAD-binding domain is longer in HxnS. Within the FAD/NAD domain, the residue corresponding to Phe417 of the *B. tauru*s XDH is almost universally an aromatic residue in XDHs (Tyr454 in HxA) but it is Ile (Ile478) in HxnS and always an aliphatic hydrophobic residue in HxnS orthologues (figure 2). The carboxy-terminal MOCO/substrate-binding domain (starting from residue 590 in the *B. taurus* XDH) shows an almost complete conservation of both the residues interacting with MOCO [25] and those interacting with substrates, including most of the residues that line the substrate access channel. His954 of the *B. taurus* enzyme, a residue not involved in the enzyme active site, is conserved in HxA (His985) and in most of its orthologues. However, it is Pro (Pro1008) in HxnS (figure 2) and in all its putative orthologues. This change does not affect the modelled secondary structure (not shown, but see below). Other amino acid residues, which differ systematically among HxA and HxnS orthologues (see section below), correspond to some of the residues involved in MOCO binding; the Val1081 and Ser1082 of the *B. taurus* enzyme are Ala1112 and Ser1113 in HxA but Ser1137 and Gly1138 in HxnS (figure 2). Conserved residues include Arg880 of *B. taurus* XDH (Arg911 of HxA and Arg934 of HxnS), a residue that is never conserved in XDH-like aldehyde oxidases [9,29–31]. Mutations affecting this residue in *hxA* result in altered substrate specificity including a PHII-like resistance to allopurinol inhibition and the inability to accept xanthine as a substrate [18,29]. Glu803 of the *B. taurus* enzyme is conserved in HxA (Glu833) and HxnS (Glu856). This key residue is never conserved in XDH-like aldehyde oxidases [30]. Within the HxA MOCO/substrate-binding domain, several mutations result in either loss-of-function or altered substrate specificity phenotypes [29]. All the corresponding residues involved are conserved in HxnS (figure 2). The pair of aromatic amino acids that sandwich the purine ring and orient the substrate towards the MOCO are conserved (Phe914 and 1009 in the *B. taurus* enzyme, 954 and 1040 in HxA, 968 and 1064 in HxnS).

A striking exception to the sequence conservation is the insertion of an Ala (Ala1065 in HxnS) between the almost universally conserved Phe1009 and Thr1010 (numeration as in the *B. taurus* enzyme, Phe1040 and Thr1041 in HxA, Phe1064 and Thr1066 in HxnS; conserved in all characterized XDHs but not in the eukaryotic XDH-like aldehyde oxidases [30], figure 2). The Phe/Thr pair is also conserved in bacterial XDHs (residues 459 and 460 in subunit B of the *Rhodobacter capsulatus* XDH [32]). An Ala insertion at this position is an almost absolute feature of HxnS orthologues (FATAL in HxnS orthologues, FSTAL in *Choiromyces venosus* putative HxnS, compared with FTAL in all Pezizomycotina HxA orthologues). Phe1013 is universally conserved in XDHs (Phe1044 in HxA), but it is a His (His1069) in HxnS (figure 2) and its putative orthologues. HxA and HxnS can be modelled to and superimposed on the structure of the *B. taurus* XDH (electronic supplementary material, figure S2). While modelling the active site, no obvious differences can be seen in the orientation of the relevant active-site residues with the obvious exception of the orientation of Thr1066 of HxnS compared with Thr1041 (HxA) and Thr1010 (*B. taurus* XDH). This residue participates in the active site by interacting with the carbonyl group of Phe1009 [33]. The hydroxyl group of Thr1010 is involved in the binding of several inhibitors [33–35], but more importantly, either the N1 or the N7 of hypoxanthine [36]. The corresponding Thr460 (within an FTLTH motif) of the B subunit of the *Rhodobacter capsulatus* XDH has been shown to hydrogen-bind the N7 of hypoxanthine but the O6 of xanthine [34]. Further work should show whether the change of orientation of the Thr residue is the key feature that allows presentation of the nicotinate molecule to the MOCO centre.

### 2.3. Phylogeny of fungal purine hydroxylases

**Figure 4.**
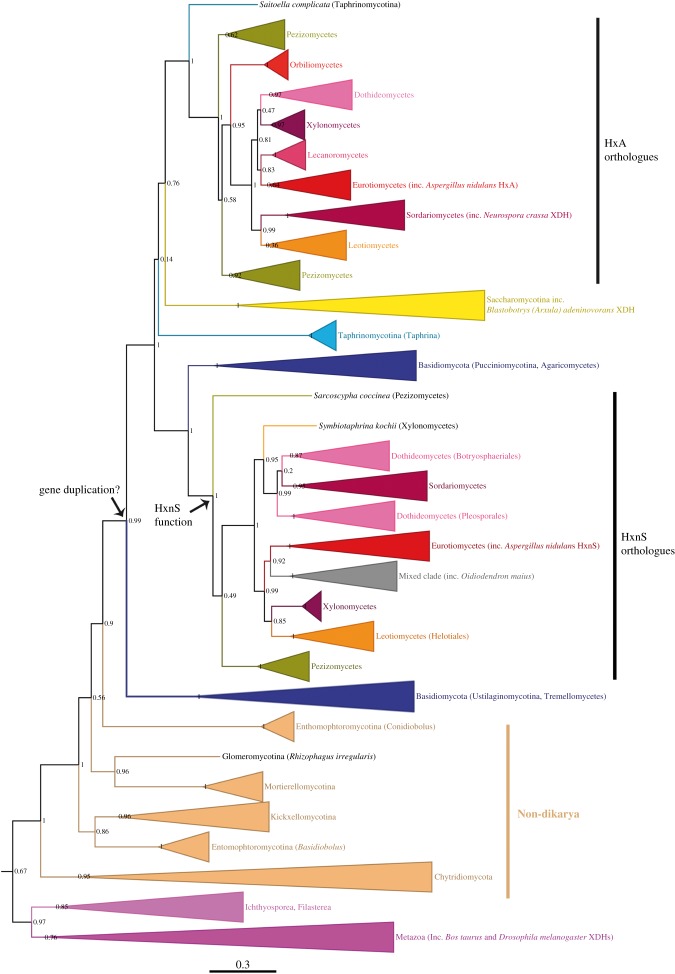
A simplified phylogeny of the fungal purine hydroxylases, HxA (PHI)-like and HxnS (PHII)-like. This tree in cartoon form was extracted from the more complete tree shown in the electronic supplementary material, figure S3, where all species used are indicated. Outgroups are the nearest non-fungal taxa of the Opisthokonta. Values at nodes are aLRTs (approximate likelihood ratio tests). The arrows indicate the putative nodes where the gene duplication and the PHII neo-functionalization occurred.

The *hxnS* gene probably resulted from duplication and divergence of an ancestral *hxA* gene [7]. We searched all available fungal genomes for homologues of XDH (see electronic supplementary material, figure S3 and table S1). Enzymes of this group are absent from *Rozella allomyci*s (Cryptomycota), the Microsporidia, the Neocallimastigomycota and the Mucoromycotina. Figure 4 and the electronic supplementary material, figure S3 show the distribution of XDH-like enzymes among all fungal taxa. XDH-like enzymes are present in all classes of the Pezizomycotina, basal species of the Taphrinomycotina and Saccharomycotina, and some members of the Basidiomycota (see below). The peptidic sequence of the outgroups strongly suggests that the basal enzyme was a typical XDH.

No *hxnS*-like gene is present outside the Pezizomycotina. Both *hxA* and *hxnS* orthologous genes are present in the basal class Pezizomycetes, while *hxnS* orthologues are absent from the sequenced species of Orbiliomycetes and Lecanoromycetes. With the exceptions of *Oidiodendron maius* and *Rhytidhysteron rufulum* (see electronic supplementary material, figure S3 legend), all species of the Pezizomycotina, where a putative orthologue of HxnS is present, also carry an orthologue of HxA. Loss of *hxnS* orthologues has occurred within the Eurotiomycetes: orthologues of HxA are present in all species available, but the presence of HxnS is patchy, i.e. present in the *nidulantes* group and the black aspergilli, but not, for example, in *A. flavus.* With the exception of *Penicillium paxilli* and *P. citrinum*, which contain *hxnS* orthologues (unlinked to *hxnR;* see below), the *hxnS* orthologues are missing from species of *Penicillium*. Within the Sordariomycetes, a similar pattern of loss occurs, with *hxnS* orthologues present in the Nectriaceae (order Hypocreales), but not in the Sordariales (such as *Neurospora crassa*, *Sordaria macrospora* and *Podospora anserina*)*.* The only PH-like enzyme present in *O. maius* (Leotiomycetes) could represent a second neo-functionalization, in which an enzyme phylogenetically related to HxnS would have reacquired HxA substrate specificity (see comments to electronic supplementary material, figure S3). The phylogeny (figure 4; electronic supplementary material, figure S3) strongly suggests a duplication of an HxA ancestral gene occurring at the root of the Dikarya. This duplication would have been followed by either neo-functionalization, leading to HxnS (in the Pezizomycotina) or loss of one of the two ancestral paralogues with HxA function (elsewhere in Dikarya). This discrepancy between the timing of duplication and neo-functionalization would account for the two separated clades of the XDHs of the Basidiomycota (one of them clustering with HxnS orthologues), the divergence of *Saitoella complicata* and the *Taphrina* spp., and the position of both the Saccharomycotina and Taphrinomycotina as outgroups of HxA orthologues rather than as an outgroup of all the Pezizomycotina PHs (see electronic supplementary material, figure S3 legend).

The *hxnS* orthologues, which have been included in figure 4 and the electronic supplementary material, figure S3, show a highly variable exon/intron structure, as discussed in the supplementary material (comments on the exon–intron structure of *hxnS* orthologues).

### 2.4. Identification and characterization of the *hxnR/aplA* gene

**Figure 5.**
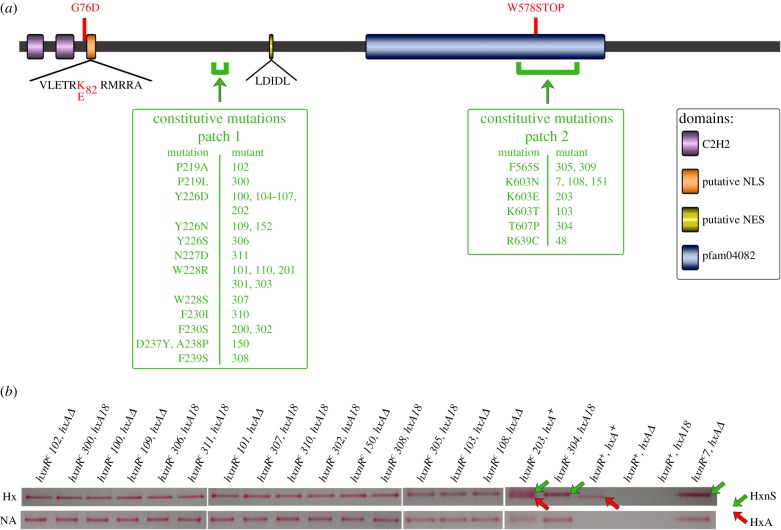
A schematic representation of the HxnR transcription factor and verification of constitutivity of *hxnR^c^* mutants. (*a*) A schematic of the HxnR transcription factor is shown, indicating the two Cys2His2 Zn-finger domains (C2H2, in purple), the putative nuclear localization signal (NLS, in orange), the putative nuclear export signal (NES, in yellow), the fungal transcription factor domain (pfam04082, in blue) and the two regions where the constitutive mutations occur (in green). The three extant loss-of-function mutations are indicated in red letters in the scheme. All the amino acid changes leading to constitutivity are indicated, together with the cognate allele number (in green). (*b*) Enzyme activity staining for hypoxanthine hydroxylase (Hx) and nicotinate hydroxylase (NA) of the constitutive mutants is shown. Only HxnS (PHII), which has a lower mobility than HxA (PHI), stains with nicotinate as the substrate. Note its complete absence in the wt strain *hxnR^+^ hxA^+^* grown under non-inducing conditions, while HxA shows substantial basal levels as reported previously [1,38]. As the constitutive mutations were isolated in different *hxA* backgrounds (*hxA18*, *hxAΔ*, *hxA^+^*; see ‘Material and methods’ section and electronic supplementary material, table S2), this is also indicated. All mycelia were grown in non-inducing conditions (for either HxA or HxnS) on 1 mM acetamide as the nitrogen source for 15 h at 37°C.

Closely linked to, but separated by locus AN9177 (to be called *hxnT*; see below), there is an open reading frame of 2673 nt (interrupted by a single 75 nt intron) encoding a protein of 865 residues comprising two typical Cys2His2 Zn fingers near its amino terminus (AN11197). We have re-sequenced this region (GenBank accession number KX669266). In the *A. nidulans* open reading frame, there are two possible in-phase initiation codons separated by three residues (MKAKM; electronic supplementary material, figure S4). In other aspergilli available in the databases, only the second Met codon is present. As the first codon is within the transcribed sequences (RNAseq data, J-Browse module at http://www.aspgd.org/), we have assumed that this is the genuine start codon in *A. nidulans* (in accordance with Kozak [37]). Between residues 394 and 668, a PFAM domain ‘Fungal transcription specific domain’ PF04082 was detected (figure 5*a*). A nuclear localization signal from residue 77 to 87 (NLS, VLETRKRMRRA) downstream from the Zn fingers is strongly predicted by cNLS mapper, while a nuclear export signal (NES, LDIDL) is predicted for residues 285–289 by NetNES (figure 5*a*).

We deleted the whole AN11197 coding region. The resulting phenotype is identical to that reported previously for *hxnR* loss-of-function mutations [1,3,22] (figure 3; electronic supplementary material, figure S1 and transcriptional phenotypes in section ‘A tightly co-regulated gene cluster in chromosome VI’): inability to use nicotinate and 6-OH nicotinic acid as sole nitrogen sources, to which we can add now the inability to use 2,5-dihydroxypyridine, an intermediate in the catabolism of nicotinate in bacterial species [39,40]. Figure 3 confirms that 2,5-dihydroxypyridine is an inducing intermediate in *A. nidulans* as this metabolite allows strong growth on hypoxanthine in the presence of allopurinol, which necessitates induction of *hxnS*.

Extant loss of function, as well as constitutive mutations (*alpA^c^* mutations; see ‘Introduction’ section) map within the *hxnR* open reading frame (figure 5*a*). We have thus renamed the constitutive regulatory mutations, *hxnR*^c^. We attempted to define the domain(s) comprising residues mutable to constitutivity by selecting and sequencing additional *hxnR^c^* mutations (see ‘Material and methods’ section). All sequenced mutations are shown schematically in figure 5, while the mutational changes are detailed in the electronic supplementary material, table S2. As some mutational changes were detected several times, in separate mutation runs, we have probably near-saturated the *hxnR* gene with constitutive mutations.

We constructed a CONSURF profile of the HxnR protein, using putative orthologues from 123 species of the Pezizomycotina subphylum (electronic supplementary material, figure S4 and table S3). All missense mutations, either constitutive or loss-of-function, map in highly conserved regions (figure 5). Constitutive mutations map in two patches, one well-defined patch between residues 219 and 239, the other, a larger domain between residues 565 and 639. For a number of residues we have obtained several different amino acid changes. Accessible aromatic residues at positions 226 and 228 and a basic residue at position 605 seem necessary for HxnR to be in its default, inactive state, in the absence of its physiological inducer.

We detected putative HxnR orthologues only among the Pezizomycotina (electronic supplementary material, table S3). We would expect a strong correlation between the presence of *hxnR* and *hxnS* orthologues. Out of 139 species of the Pezizomycotina screened, 40 have only *hxnR* and 14 only *hxnS* (electronic supplementary material, table S4). Among the 85 species where both genes are extant, tight clustering is evident in most of them (see the section ‘Conservation of the hxn gene cluster in the Aspergillaceae’). The absence of clustering is common among the Sordariomycetes, with the exception of the Xylariales order where the clustering is maintained. These 85 species include all classes of the Pezizomycotina subphylum with the exception of the Orbiliomycetes and the Lecanoromycetes.

### 2.5. A tightly co-regulated gene cluster in chromosome VI

**Figure 6.**
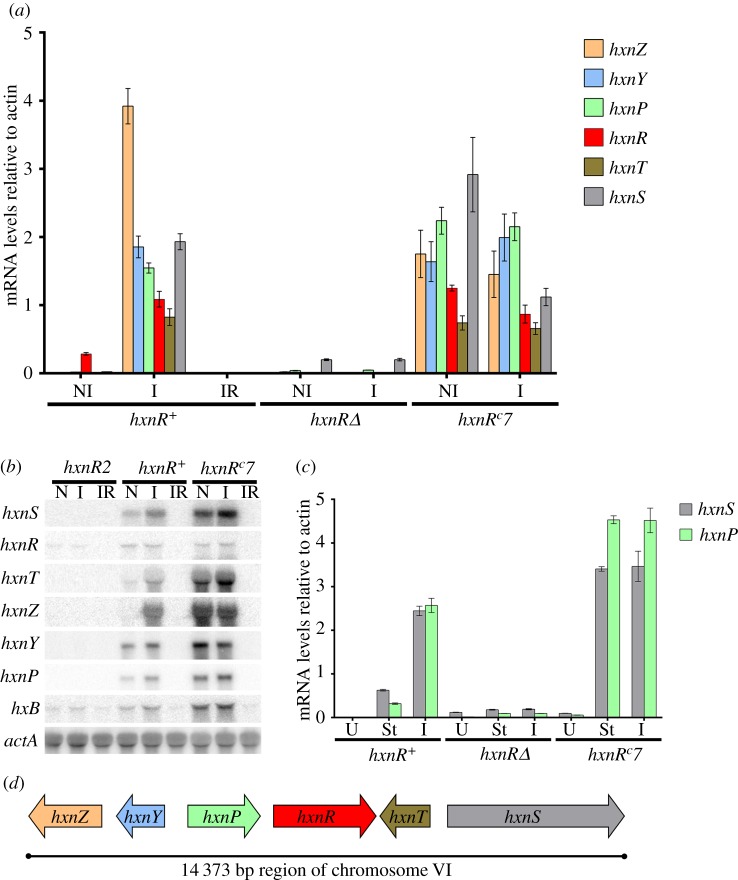
Co-regulation of the genes in the *hxn* cluster. (*a*) mRNA levels measured by qRT-PCR for all the genes in the *hxn* cluster. Mycelia were grown on 1 mM acetamide as the sole nitrogen source for 8 h at 37°C. They were either maintained on the same medium for a further 2 h (non-induced, NI) or induced with 1 mM nicotinic acid (as the sodium salt, I) or induced as above together with 5 mM of l-(+)diammonium-tartrate (induced repressed, IR). Strains used: *hxnR^+^* (FGSC A26), *hxnRΔ* (HZS.136) and *hxnR^c^7* (FGSC A872). (*b*) Northern blot showing qualitatively the co-regulation of all the genes in the cluster under different growth conditions. Mycelia were grown on 500 µM urea for 8 h, and then transferred to 1 mM acetamide for an additional 2 h (non-induced, NI) or to the same plus 1 mM nicotinic acid (as above, I) or to the latter together with 5 mM l-(+)diammonium-tartrate (induced repressed, IR). Together with *hxnS*, *hxnR*, *hxnT*, *hxnP, hxnY* and *hxnZ* transcripts we also monitored the expression of *hxB*, an unlinked gene, which was previously shown to be under the control of HxnR [41]. As a loading control, the expression of *acnA* (actin) was monitored. Strains used are indicated by the relevant mutation: *hxnR^+^* (FGSC A26), *hxnR2* (CS302), a missense unleaky mutation (Gly76Asp) and *hxnR^c^7* (FGSC A872), our standard constitutive mutation (figure 5; electronic supplementary material figure S4 and table S2). (*c*) Expression of *hxnS* and *hxnP* under conditions of nitrogen starvation. Mycelia were grown on 5 mM urea as the sole nitrogen source for 8 h, and then transferred to the same medium for two additional hours (U, which is non-inducing and actually partially repressed conditions; see text) or to a medium without any nitrogen source (starvation media, St) or to a medium with 10 mM nicotinic acid as the nitrogen source (inducing media, I). Strains as in panel (*a*). In all qRT-PCR experiments, data were processed according to the standard curve method with *acnA* as the control mRNA. Standard errors of three independent experiments are shown in all qRT-PCR. Gene probe primers are detailed in the electronic supplementary material, table S6. (*d*) Cluster arrangement of the *hxn* genes on chromosome VI.

**Figure 7.**
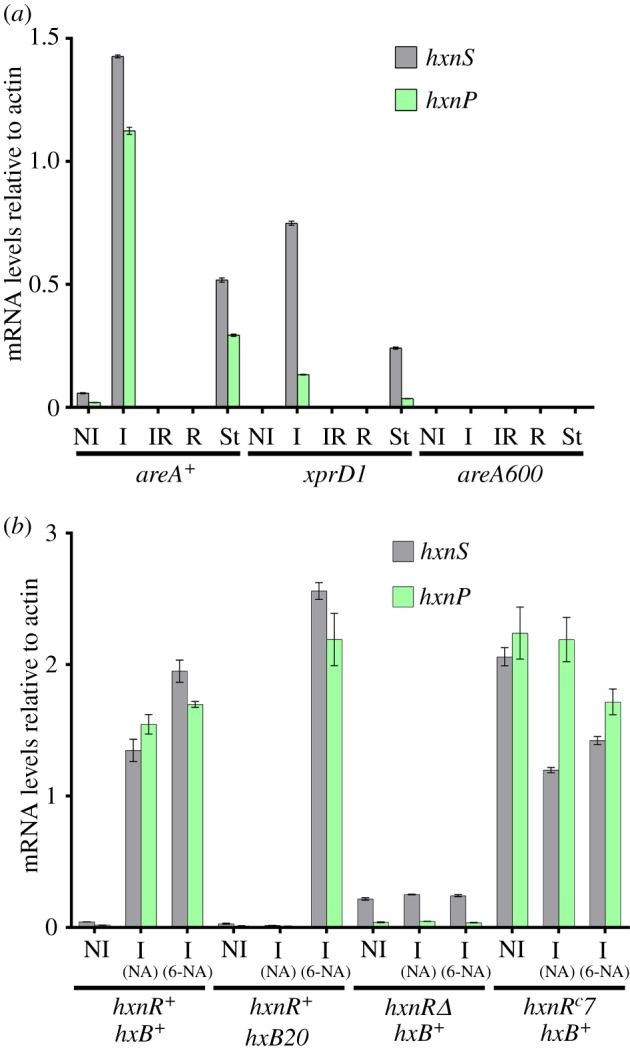
Induction depends on the AreA GATA factor and on the metabolism of nicotinic acid. (*a*) The GATA factor AreA is essential for *hxnP* and *hxnS* expression. *hxnP* and *hxnS* mRNA levels in *areA^+^* (FGSC A26) and an *areA* supposedly derepressed mutant (*xprD1*, HZS.216) and *areA* null mutant (*areA600*, CS3095) strains. Non-induced conditions (NI): Strains were grown on MM media with 5 mM l-(+)diammonium-tartrate as the sole N-source for 8 h, and then the mycelia were transferred to MM with 1 mM acetamide for further 2 h. Induced conditions (I): as above but transferred to 10 mM nicotinic acid as the sole N-source. Induced repressed conditions (IR): transferred to 10 mM nicotinic acid and 5 mM diammonium-tartrate. N-starvation conditions (St): transferred to nitrogen-free medium. (*b*) Induction depends on metabolism of nicotinic acid via HxnS activity. mRNA levels of *hxnP* and *hxnS* in *hxnR^+^ hxB^+^* (FGSC A26), *hxnR^+^ hxB20* (HZS.135), *hxnRΔ hxB^+^* (HZS.136) and *hxnR* constitutive, *hxnR^c^7 hxB^+^* (FGSC A872) strains are shown. Non-induced (NI) and induced growth conditions were the same as detailed in (*a*). (NA): induced with 1 mM nicotinic acid; (6-NA): induced with 1 mM 6-OH nicotinic acid. The *hxB20* mutation abolishes completely HxnS activity without affecting its expression as judged by measuring its CRM [12]. qRT-PCR data in both panels were processed according to the standard curve method; the housekeeping control transcript was actin (*acnA*). Standard deviations based on three biological replicates are shown.

In *A. nidulans*, the *hxnR* and *hxnS* genes are within a cluster of co-regulated genes. This is shown in figures 6 and 7. Six neighbouring genes, inducible by nicotinate and 6-OH nicotinate, are non-inducible in strains carrying either the *hxnR2* or *hxnRΔ* mutations and show strong constitutive expression in the *hxnR^c^7* background. The genes in the cluster are: *hxnS* (AN9178), *hxnT* (AN9177), *hxnR* (AN11197), *hxnP* (AN11189*), hxnY* (AN11188) and *hxnZ* (AN11196) (figure 6*d*). The flanking genes AN9179 (adjacent to *hxnS*) and AN9174 (adjacent to *hxnZ* and transcribed convergently) are not induced by nicotinate and they are not affected by *hxnR* constitutive or loss-of-function mutations (not shown). The *hxnP* and *hxnZ* genes encode transmembrane proteins of the Major Facilitator superfamily (PF07690.13). *hxnT* encodes a flavin oxidoreductase (Oxidored_FMN, PF00724), while *hxnY* encodes a typical α-ketoglutarate-dependent dioxygenase (PF14226.5 and PF03171.19). The role of each gene in nicotinate utilization and their phylogenetic relationships will be discussed elsewhere, HxnP and HxnZ being involved in the uptake of nicotinate-derived metabolites, and HxnT and HxnY in the further metabolism of 6-OH nicotinic acid (E Bokor, M Flipphi, J Ámon, C Scazzocchio and Z Hamari, unpublished results). We can however state that, for each of these genes, the nearest homologue is a fungal and not a bacterial gene (not shown). *hxnR* is itself an inducible gene (figure 6*a,b*). There is a clearly detectable level of *hxnR* transcript under non-induced conditions, at variance with the other genes of the cluster. RNAseq data [23,42], available in J Browse (http://www.aspgd.org/), confirm the co-regulation of the cluster, where all genes in this cluster are non-expressed in conditions of nitrogen sufficiency and derepressed by nitrogen starvation. Under our experimental conditions, with the exception of *hxnR,* genes in the cluster are virtually non-expressed in media that contain good nitrogen sources but are expressed under nitrogen-starved conditions (figure 6*c*). All genes in the cluster are drastically repressed by ammonium (figures 6*b* and 7*a*). HxnR is necessary for expression under nitrogen-starved conditions (figure 6*c*). The strong constitutivity of *hxnR^c^7* strains is clear in the presence of a non-repressive nitrogen source (acetamide) or under conditions of nitrogen starvation. The transcript of *hxB*, which had previously been found to be independently regulated by HxnR and UaY (and thus independently induced by nicotinate and uric acid [41]) behaves qualitatively as the five structural genes in the cluster (figure 6*b*).

The role of AreA, the GATA factor mediating nitrogen metabolite derepression [43–45], is shown for *hxnS* and *hxnP* in figure 7*a*. Transcription of both *hxnS* and *hxnP* is abolished in a strain carrying a null *areA* mutation (*areA600*) under all conditions, including nitrogen starvation. Surprisingly, the transcription of both *hxnS* and *hxnP* is diminished in a strain carrying an *xprD1* mutation (considered to be the most extremely derepressed allele of *areA*, ([46] and references therein)); the allele is called *xprD1* for historical reasons [43,47]. By contrast with the genes of the nitrate and purine assimilation pathways [48–50], the glutamate–aspartate transporter gene *agtA* [51] and also *hxB* [41], the *hxnS* and *hxnP* genes are fully repressed by 10 mM ammonium in an *xprD1* strain. A similar atypical effect has been reported for the main urea transporter *ureA* gene [52].

A downstream metabolite of nicotinate is the physiological inducer of the HxnS protein [12]. Figure 7*b* shows this to be the case at the level of mRNA steady-state levels for both *hxnS* and *hxnP*. In an *hxB* null mutant lacking HxnS activity, nicotinate does not behave as an inducer but 6-OH nicotinate does. Thus the effector of HxnR is not nicotinate but 6-OH nicotinate or a metabolite further downstream the nicotinate utilization pathway. The *in vivo* test shown in figure 3, where 2,5-dihydroxypyridine acts as inducer, suggests the latter to be the case.

### 2.6. Conservation of the *hxn* gene cluster in the Aspergillaceae

**Figure 8.**
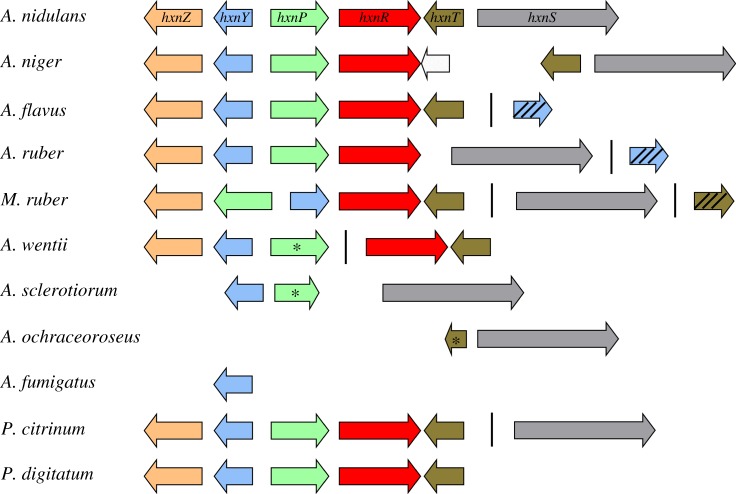
Hxn cluster organization in the Aspergillaceae family. Boxes indicate genes; arrowheads indicate orientation. Colour stands for the orthologues found in different species (*Aspergillus nidulans*, *A. niger*, *A. flavus*, *A. ruber*, *Monascus ruber*, *A. wentii*, *A. sclerotiorum*, *A. ochraceoroseus*, *A. fumigatus*, *Penicillium citrinum*, *P. digitatum*). Stars indicate putative pseudogenes (putative non-functional alleles); hatched boxes indicate duplicated paralogues. Vertical lines symbolize physical unlinkage of genes on the same chromosomes. The blank box in *A. niger* stands for the orthologue of the *A. nidulans* gene at locus AN8360 (encoding a nitroreductase of bacterial origin), which is unlinked to the cluster in the latter fungus, while its expression is not regulated by nicotinate or the transcription factor HxnR.

The evolution of the whole nicotinate utilization pathway in fungi will be dealt with in another publication (E Bokor, M Flipphi, J Ámon, C Scazzocchio and Z Hamari, unpublished results), but we discuss here the conservation of the *hxn* cluster in the Aspergillaceae family. Examples of the organization of the cluster are shown in figure 8. Episodes of gene gain and loss are shown, including the duplication of *hxnY* or *hxnT* as well as the loss of *hxnT* and *hxnS*. In *A. ochraceoroseus*, only *hxnS* is present, a mirror image of the situation in *A. flavus* (and other species in section Flavi) and *P. digitatum* (and all other *Penicillium* species but two), where the genome includes all *hxn* genes with the exception of *hxnS*. The absence of *hxnS* may imply that, in these species, the cluster deals with the utilization of nicotinate derivatives (such as 6-OH nicotinic acid) rather than that of nicotinate *per se*.

The situation in *P. citrinum* (and *P. paxilli*) implies a secondary reconstitution of the pathway by horizontal transmission, as an unlinked orthologue of *hxnS* is present, seemingly reacquired from a member of the Hypocreales (order of Sordariomycetes; see Phylogeny section, electronic supplementary material, figure S3). In *A. niger*, and related black aspergilli albeit not in *A. carbonarius* or *A. aculeatus*, a new, intronless gene, encoding a putative nitroreductase is inserted in the cluster, between *hxnR* and *hxnT* (see ‘Discussion’ section).

## 3. Discussion

### 3.1. A nicotinate-inducible eukaryotic cluster

With the exception of some of our own old work (see ‘Introduction’ section) no genes or enzymes involved in the degradation of nicotinate have been described in any eukaryote. Degradation of nicotinic acid has been studied in plant cell cultures and tea plant material fed with carboxyl-C^14^-nicotinic acid and C^14^-6-nicotinic acid, monitoring the formation of ^14^CO_2_ [53–55]. No enzymes involved in these processes were identified and inspection of relevant genomes only revealed one typical XDH. Thus, the *A. nidulans* HxnS is the hitherto only eukaryotic nicotinate hydroxylase studied, and the *hxn* gene cluster we have identified is the first co-regulated eukaryotic gene cluster involved in the utilization of nicotinate ever described.

### 3.2. The *hxA/hxnS* duplication compared to other eukaryotic MOCO-enzyme duplications

Neo-functionalization of enzymes of the XDH group arising from ascertained or presumed gene duplications occur in both prokaryotes and eukaryotes. Fetzner and co-workers have described the diversity of bacterial MOCO enzymes of the XDH group, even if the phylogeny of these enzymes with different specificities remains unstudied [39,56–59]. Duplication and neo-functionalization of genes encoding XDH-like enzymes are widespread in Metazoa, studied mainly in insects and vertebrates. In Metazoa, a close linkage of the neo-functionalized genes with strict conservation of intron/exon structure is the rule [30,60–63]. The implication is that XDH gene duplication has occurred by different mechanisms in metazoans and fungi. In metazoans, duplication seems to occur at the DNA level by unequal crossover. In the fungi, the striking amino acid sequence conservation among the HxA/HxnS paralogues together with the variability of intron positions suggests that the duplication of an HxA ancestral gene occurred via retroposition, followed by a re-intronization either after or concurrent with re-functionalization of the duplicated gene. Notwithstanding the mechanism underlying this gene duplication, the *hxA/hxnS* duplication is quite ancient, occurring before the divergence of the Taphrinomycotina from other Ascomycota (greater than 400 Ma [64,65]), which allows the possibility of intron loss and reinsertion. The variation of intron–exon organization in both the *hxA* and *hxnS* clades (not shown) is also consistent with this possibility (figure 4 and electronic supplementary material, figure S3, for the relevant positions in the phylogenetic tree).

### 3.3. Convergent evolution of bacterial and fungal nicotinate hydroxylases

MOCO enzymes able to catalyse the hydroxylation of nicotinate to 6-OH nicotinate have been described in a variety of bacterial species [40,66–68]. However, it can be excluded that the HxnS proteins have originated by horizontal transmission from bacteria. In all eukaryotes, XDH-like enzymes are dimers of chains of approximately 1500 amino acid residues comprising three discrete domains (figure 2). In bacteria, these domains are encoded by at least two genes, one specifying a small subunit carrying the 2Fe/2S centres and the FAD-binding sequences and a large subunit carrying the MOCO and substrate-binding centres. These genes, included in an operon, reflect the amino- to carboxy-terminus order of the domains in the eukaryotic XDH-like enzymes. A similar structure occurs in bacterial nicotinate hydroxylases [40,67], which makes improbable a direct bacterial origin of the fungal nicotinate hydroxylases. Figure 4 and the electronic supplementary material, figure S3 show that HxnS orthologues have originated by gene duplication within the fungal kingdom, possibly at the root of the Dikarya, with a neo-functionalization process occurring within the Pezizomycotina subphylum. BlastP screening with the MOCO/substrate-binding domains of both HxA and HxnS (figure 2) against all bacterial sequences available in the NCBI non-redundant protein (nr/nt) database yielded homologues of the MOCO-binding subunits of putative bacterial XDHs, but in no case (among the first 100 sequences) were MOCO subunits of known bacterial nicotinate hydroxylases found (not shown). Thus, fungal nicotinate dehydrogenases show more similarity to bacterial XDHs (and as a matter of course, to all genuine eukaryotic XDHs) than to bacterial nicotinate dehydrogenases.

A comparison of the sequences of the MOCO and substrate-binding subunits of bacteria suggests that there are at least three classes of MOCO nicotinate hydroxylases, exemplified by *Pseudomonas putida*, *Eubacterium barkeri* and *Bacillus niacini,* respectively [40,66,67]. We have stated that the insertion of an Ala residue (HxnS Ala1065) between the conserved Phe and Thr in the active site is a signature of fungal HxnS orthologues (see §§2 and 3 of Results). Genuine bacterial XDHs also carry an FT motif in the cognate place in the structure [32,34]; however, the divergence of bacterial nicotinate hydroxylases from bacterial XDHs is such that neither sequence alignments nor structural modelling (not shown) gave a clear inkling of which modifications resulted in, or at least correlate with the shift in substrate specificity. It seems that not only has there been convergent evolution of fungal and bacterial nicotinate hydroxylases, but that nicotinate hydroxylases evolved several times independently within bacteria. A hint to the shift in specificity towards hydroxylation of nicotinic acid is provided by the molecular structure of the nicotinate hydroxylase of *E. barkeri* [68,69]. In this enzyme, the substrate/MOCO-binding domain is split into two independent peptides (L and M). Strikingly, it carries a selenium rather than a sulfur atom as the terminal ligand to the Mo(VI). Selenium also occurs in the XDH of this organism [68,69], which is consistent with an independent evolution of the *E. barkeri* nicotinate hydroxylase from that of other bacterial enzymes of similar specificity (see above). Most active-site residues are conserved, with an interesting exception. Tyr13 of subunit M (Tyr13_M_) is modelled to hydrogen-bind the heterocyclic N atom of nicotinate by its hydroxyl group [68]. The corresponding residue in the *B. taurus* XDH is Phe1005, which has not been proposed to interact with the substrate [24,27]. This Phe residue, four residues upstream of Phe1009 (of *B. taurus*), is conserved in both HxA and HxnS (figure 2) and indeed in all HxA and HxnS-like fungal enzymes included in the electronic supplementary material, figure S3, with the exception of the putative XDH of the four divergent *Taphrina* species, where it is substituted by a His. Tyr13_M_ is not conserved in several other characterized or putative nicotinic acid hydrolases such as those of *Ps. putida* and its putative orthologues, where the corresponding residue is an Arg or a His. No sequence similar to FTAL or FATAL is present in the enzyme of *E. barkeri* and its putative orthologues (see fig. S1 of [68]). It is tempting to speculate that the change in orientation of Thr1066 in HxnS allows an interaction with nicotinate by its hydroxyl group similar to that seen for modelled Tyr13_M_ in *E. barkeri.* Biochemical evidence indicates that the carboxyl group of nicotinate is essential for substrate binding of HxnS [11]; the hydroxyl of Thr1006 could potentially hydrogen-bind the carboxyl group of nicotinic acid. Differently from HxnS, the enzyme of *E. barkeri* does not accept hypoxanthine as substrate [68]. It can be proposed that the bacterial enzymes (at least the *E. barkeri* one) have fully evolved into dedicated nicotinate hydroxylases, while the HxnS orthologues conserve properties of XDH. The specific situation discussed for the homologue of *O. maius,* which can be proposed to have reverted to a typical XDH activity from an HxnS-like enzyme (see electronic supplementary material, figure S3 legend), would be in line with this speculation.

### 3.4. An unusual specific transcription factor

Fungal transcription factors regulating specific metabolic primary or secondary pathways are generally of the Zn2Cys6 (zinc cluster) class, while Cys2His2 (zinc finger) factors are usually, with very few recorded possible exceptions [70,71], broad domain regulators of either metabolism and/or morphology. The closest characterized transcription factor that shares architecture and has sequence similarity with HxnR is Klf1p of *Schizosaccharomyces pombe*. This factor is necessary for maintenance of long-term quiescence and its absence results in abnormal cell morphology in the quiescent state [72]. The nearest homologue and possible orthologue of Klf1p in *A. nidulans* is the protein of unknown function encoded by AN6733. The latter is strictly conserved in a syntenic position in all aspergilli included in the AspGD database and putative orthologues are present in all sequenced members of the Pezizomycotina (not shown). As *hxnR* is only present in the Pezizomycotina, it is tempting to speculate that it originated from a duplication of the possibly essential ancestral orthologue of AN6733, the duplicated gene being then recruited into the nicotinate utilization pathway.

The apparent high frequency of constitutive, gain-of-function mutations, and their mapping indicate that the HxnR protein is, in the absence of inducer, in a default state non-competent to elicit transcription, and that the domains where constitutive mutations map are instrumental in maintaining HxnR in this ‘closed’, inactive state. The amino-terminal cluster of constitutive mutations maps outside the PF04082 domain, in sequences that are conserved only among HxnR orthologues. The carboxy-terminal mutations map within the PF04082 domain conserved in Klf1, AN6733 and NCU05242 (the *N. crassa* orthologue of AN6733). Note, for example, mutations affecting Lys603 in HxnR, a residue conserved in these four proteins (figure 5; electronic supplementary material, figure S4). The PF04082 domain of Gal4p (244–537) coincides with the central regulatory domain of similarity proposed by Poch [73]; see also Stone & Sadowski [74]. The cognate domain of the *A. nidulans* NirA (pathway-specific regulation of nitrate assimilation) spans residues 230–487 [75]. Within this region maps a cryo-sensitive, non-inducible mutation (Arg347Ser) as well as its intragenic suppressors, some of which result in constitutivity. This domain possibly interacts with both the NES and the C-terminal transcription activation domain [75]. The evidence from different systems indicates that PF04082 is an intramolecular interaction domain. Thus, the proposed neo-functionalization of HxnR would have involved the modification of the sequence between residues 208 and 239 (electronic supplementary material, figure S4), as a module interacting with PF04082.

### 3.5. The evolution of clustering

Old genetic and newly acquired data, which will be reported elsewhere (E Bokor, M Flipphi, J Ámon, C Scazzocchio and Z Hamari, unpublished data), established that not all the genes involved in nicotinate catabolism are within the *hxnZ–hxnS* gene cluster. We have described the conservation of this cluster within the Aspergillaceae. We discussed the origin of both *hxnS* and *hxnR* within the Pezizomycotina subphylum. While the selective pressures that led to the conservation of clustering of genes of a specific metabolic pathway have been the subject of animated discussion [76–78], we have no inkling of the recombination processes that led to clustering of the *hxn* genes in the first place. A model of recent local gene duplication can be excluded for the origin of all genes in the cluster, each nearest paralogue in the same organism being in every case unlinked and actually on a different chromosome (not shown). Within the Aspergillaceae, *A. nidulans* represents the possible primeval situation, with a pattern of both loss and duplication for other members of this family (figure 8). Recent duplication has occurred for some of the genes in the cluster. In *Monascus* sp*.* (exemplified by *M. ruber* in figure 8) an unlinked paralogue of *hxnT* is extant, showing 58% amino acid identity with the copy within the cluster and a strict conservation of intron positions. Duplicated paralogues of *hxnY* occur in the *flavii/nomius* group and in species of the section Aspergillus. The fact that these duplicated genes are unlinked to the cluster excludes a model of duplication by unequal crossover.

It is noteworthy that instances of duplications are coupled with instances of loss. Duplication of *hxnY* in the section Aspergillus (exemplified by *A. ruber*, figure 8) is coupled with the loss of *hxnT*, while that of *hxnT* in section Flavi (exemplified by *A. flavus*, figure 8) is coupled with the loss of *hxnS*. This coupling may result from just one single recombination event. Note that in *M. ruber*, where there is no gene loss, duplication of *hxnT* coincides with the separation of *hxnS* from the cluster. The duplication of *hxnY*, with conservation of (some) intron positions, seems to have occurred before the divergence of the *flavi* and the *fumigati* groups. Remarkably, only the duplicated *hxnY* paralogue is retained in *A. fumigatus* and *Neosartorya fischeri*.

Horizontal transmission from pre-existent clusters has been established for both primary and secondary metabolism pathways. It has been proposed that nitrate assimilation gene cluster of fungi was horizontally transmitted from oomycetes [79]. We can exclude such horizontal transmission as the origin of the *hxn* cluster. The nearest paralogue of all the genes comprising the cluster is another fungal gene, usually in the same organism (data to be presented elsewhere, E Bokor, M Flipphi, J Ámon, C Scazzocchio and Z Hamari, unpublished results).

One exception to this is the incorporation of an intronless nitroreductase gene into the *hxn* cluster of most aspergilli of the section *nigri* and its presence outside the cluster in four other aspergilli including *A. nidulans*. A phylogenetic analysis (not shown) establishes that this gene originates from a horizontal transfer from a cyanobacterium to an ancestral member of the Leotiomyceta (42% and 41% identity shared by the enzymes from *A. niger* and *A. nidulans*, respectively, with *nfsA* product from *Anabaena variabilis*; see [80] for a comparison of fungal and bacterial nitroreductases). Its incorporation within the *hxn* cluster of some aspergilli is quite intriguing. It may be relevant that many nitroreductases are involved in the degradation of *N*-heterocyclic compounds [81].

We have only presented a detailed phylogenetic analysis for HxA/HxnS, but work to be detailed elsewhere (E Bokor, M Flipphi, J Ámon, C Scazzocchio and Z Hamari, unpublished results) suggests that, with the one exception mentioned, all genes in the *hxn* cluster have originated from duplications within the Pezizomycotina, and that clustering followed or was synchronous with duplication. Similar evolutionary patterns for the clusters were described in fungi. A pattern of gene duplication and clustering underlies the origin and variable arrangement of the *alc* (ethanol utilization) gene cluster in the aspergilli [82]. These patterns of gene clustering resemble those described in plants, where genes organized in clusters involved in secondary metabolism originate from duplication of non-clustered genes of primary metabolism ([83–85] and references therein).

## 4. Material and methods

### 4.1. Strains, media and growth conditions

The *A. nidulans* strains used and/or constructed in this work are listed in the electronic supplementary material, table S5. Standard genetic markers are described in http://www.fgsc.net/Aspergillus/gene_list/. Complete (CM) and minimal media (MM) contained glucose as the carbon source; MMs supplemented with different N-sources were used [13,86]. The media were supplemented according to the requirements of each auxotrophic strain (www.fgsc.net). Nitrogen sources, inducers, repressors and inhibitors were used at the following concentrations: 10 mM sodium nitrate, 10 mM nicotinate (1 : 100 dilution from 1 M nicotinic acid dissolved in 1 M sodium hydroxide), 10 mM 6-OH nicotinic acid (1 : 100 dilution from 1 M 6-OH nicotinic acid dissolved in 1 M sodium hydroxide), 10 mM 2,5-dihydroxypyridine, 1 mM hypoxanthine, 5 mM l-(+)diammonium-tartrate, 5 mM urea, 1 mM acetamide as sole N-sources; 1 mM or 100 µM nicotinic acid sodium salt, 1 mM or 100 µM 6-OH nicotinic acid, 100 µM 2,5-dihydroxypyridine and 0.6 mM uric acid as inducers; 5 mM l-(+)diammonium-tartrate as repressor; 5.5 µM allopurinol as inhibitor of purine hydroxylase I (encoded by *hxA*) enzyme activity. Cesium chloride at a 12.5 mM final concentration was used in mutagenesis experiments to reduce the background growth of the nitrogen-source non-utilizer strains (http://www.fgsc.net/Aspergillus/gene_list/supplement.html#other). The strains were maintained on CM; otherwise MM with various N-sources were used in the experiments supplemented with the required vitamins. The mycelia for protein extraction were grown for 14 h at 37°C shaken at 150 r.p.m. in MM with acetamide or urea as nitrogen sources and induced when appropriate after 12 h of growth with 6-OH nicotinate. For mRNA extraction, mycelia was grown on acetamide, or urea N-sources were used for growth for 10 h at 37°C with 150 r.p.m. and after 8 h of growth, nicotinic acid, 6-OH nicotinic acid or uric acid was added to the medium as inducer and ammonium as repressor. For total DNA extraction, mycelia were grown in MM with nitrate as a N-source.

### 4.2. Mutagenesis

For UV mutagenesis, 10^9^ conidia of *A. nidulans* strains HZS.98, HZS.248 and HZS.418 in 20 ml 0.01% Tween (in a Petri dish with a 14.5 cm diameter) were exposed to UV light (Philips TUV15 W 9L1, 254 nm) with gentle shaking (50 r.p.m.) for 20 min, resulting in 95% kill. For 4-nitroquinoline 1-oxide (4-NQO) mutagenesis, conidia of HZS.248 were mutagenized as previously described [87]. Spores were plated on MM with hypoxanthine as the sole nitrogen source supplemented with 5.5 µM allopurinol and 12.5 mM cesium chloride. Strains able to grow on this medium were expected to be *hxnR* constitutive (*hxnR^c^*) mutants. The presence of allopurinol resulted in the complete inhibition of purine hydroxylase I (encoded by *hxA*) in a recipient *hxA^+^* strain (HZS.98), therefore the hypoxanthine utilization must result from the activity of purine hydroxylase II (encoded by *hxnS*), which requires either induction by nicotinate or 6-OH nicotinate or the presence of a constitutive mutation in the *hxnR* gene. In the *hxA^+^* strain HZS.98, gain-of-function allopurinol-resistant mutations at the *hxA* locus also may occur. The *hxnR^+^ hxA*-linked allopurinol-resistant mutants, however, show reduced growth on hypoxanthine compared to *hxA^+^ hxnR^c^* mutants [1,29].

### 4.3. Staining for enzyme activity in gels

Crude protein samples of mycelia were obtained from 300 ml liquid cultures incubated at 37°C with 180 r.p.m. agitation for 20 h, and induced after 15 h of growth with inducers where appropriate. Protein extraction was carried out as previously described [88]. The concentrations of crude protein samples were determined by the Bradford assay [89]. Native 10% PAGE using 0.025 M Tris, 0.19 M glycine cathode buffer (pH 8.3) according to Laemmli [90] was used to fractionate the crude extracts, containing 50 µg of protein/well. HxA- and HxnS-specific activities were detected by staining with hypoxanthine-tetrazolium [1], nicotinate-tetrazolium (100 mM pyrophosphate (pH 9.4), 1.27 mg ml^−1^ iodonitrotetrazolium chloride and 0.5 mg ml^−1^ nicotinic acid), while the diaphorase activity was detected with NADH-tetrazolium [16,91].

### 4.4. DNA and RNA manipulations

Total DNA was prepared from *A. nidulans* as described by Specht *et al*. [92]. For Southern blots [93] hybond-N membranes (Amersham/GE Healthcare) were used and hybridizations were done by DIG DNA Labeling and Detection Kit (Roche) according to the manufacturer's instructions. Transformations of *A. nidulans* protoplasts were done as described by Karacsony *et al*. [88] using a 4% solution of Glucanex (Novozymes, Switzerland) in 0.7 M KCl. For cloning procedures, *Escherichia coli* JM109 [94] and KS272 [95] were used and transformation of *Es. coli* was performed according to Hanahan [96]. Plasmid extraction from *Es. coli* and other DNA manipulations were done as described by Sambrook *et al*. [93]. Total RNA was isolated using a NucleoSpin RNA Plant Kit (Macherey-Nagel) and RNase-Free DNase (Qiagen) according to the manufacturer's instructions. cDNA synthesis was carried out with a mixture of oligo-dT and random primers using a RevertAid First Strand cDNA Synthesis Kit (Fermentas). Quantitative PCR (qPCR) and quantitative RT-PCR (qRT-PCR) were carried out in a CFX96 Real Time PCR System (BioRad) with SYBR Green/Fluorescein qPCR Master Mix (Fermentas) reaction mixture (94°C 3 min followed by 40 cycles of 94°C 15 s and 60°C 1 min). Specific primers are listed in the electronic supplementary material, table S6. Data processing was done by the standard curve method [97]. Northern blot analysis was performed using the glyoxal method [93]. In northern blots, equal RNA loading was calculated by optical density measurements (260/280 nm). [32P]-dCTP labelled gene-specific DNA molecules were used as gene probes using the random hexanucleotide-primer kit following the supplier's instructions (Roche Applied Science). DNA sequencing was done by the Sanger sequencing service of LGC (http://www.lgcgroup.com). Primers used are listed in the electronic supplementary material, table S6.

### 4.5. Gene deletions

Deletion of *hxnR* and *hxnS* was obtained by Chaveroce's method [95], which uses phage *λ* Red expressing *Es. coli* strain KS272 for obtaining the gene replacement by introducing a plasmid carrying the candidate gene and a PCR product of a transformation marker gene flanked with 50 bp regions of homology with the target DNA into the *Es. coli* strain (for details see the electronic supplementary material, Supplementary materials and methods). The obtained recombinant plasmid is then used for *A. nidulans* transformation in order to obtain an allelic exchange between the mutant allele on the plasmid and the wild-type locus. The detailed procedure is written in the electronic supplementary material, Supplementary materials and methods. The first available *hxnSΔ* strain (HZS.106 and its progeny HZS.254 used in the enzyme assays) was unfortunately found to carry additional ectopic copies of the recombinant plasmid, therefore a new *hxnSΔ* strain (HZS.599) was obtained by the gene substitution method using the double-joint PCR [98] for constructing the gene substitution cassette (see electronic supplementary material, Supplementary materials and methods). All the genetic work and growth tests were done with the new *hxnSΔ* strain.

### 4.6. *In silico* analysis

Sequence searches were carried out in both general (http://blast.ncbi.nlm.nih.gov/Blast.cgi) and specialized databases (http://www.aspgd.org/, http://genome.jgi-psf.org/programs/fungi/index.jsf). We used (with permission) 59 unpublished DNA sequences from the JGI databases; the species involved are tagged with ‘*’ in the electronic supplementary material, tables S1 and S3 (see the electronic supplementary material, table S1 footnote for further details). In every case, the gene models were manually derived by ourselves. Alignments were carried out with MAFFT (MAFFT E-INS-i and MAFFT G-INS-i); colour labelling of alignments was done with BoxShade (http://www.ch.embnet.org/software/BOX_form.html). Alignment curation for phylogeny was carried out with BMGE 1.0 (http://mobyle.pasteur.fr/cgi-bin/portal.py#forms::BMGE) [99] and maximum-likelihood phylogeny with PhyML 3.0 with automatic model selection (LG substitution model selected) [100,101] indicating approximate likelihood ratio tests [102]. Tree drawing was done with Figtree (http://tree.bio.ed.ac.uk/software/figtree/, http://mafft.cbrc.jp/alignment/server/) and localization signals were searched for at http://www.cbs.dtu.dk/services/TargetP/ [103], http://www.peroxisomedb.org/ [104], http://nls-mapper.iab.keio.ac.jp/cgi-bin/NLS_Mapper_form.cgi [105], http://wolfpsort.org/ [106], http://genome.unmc.edu/ngLOC/cite.html [107]. Structural analysis and modelling was carried out with Swiss-PdbViewer [108] and I-Tasser, (http://zhanglab.ccmb.med.umich.edu/I-TASSER/ [109,110], and model rendering with VMD 1.9. (http://www.ks.uiuc.edu/Research/vmd/) [111]. Structure superposition was done with the MultiSeq version integrated in VMD [112].

### 4.7. Statistical analysis

The significance of differences between datasets was determined by an unpaired *t*-test using the GraphPad Prism 6 software.

## Supplementary Material

Supplementary Figures

## Supplementary Material

Supplementary Figure S3 high resolution and comments

## Supplementary Material

Supplementary Tables 1-6

## Supplementary Material

Supplementary Materials and Methods

## Data accessibility

The datasets supporting this article are included in the paper and detailed in the electronic supplementary material tables. Sequences determined by us are available on GenBank (KY962010, KX585438, KX669266).
