# Supplementary material for: A eukaryotic nicotinate-inducible gene cluster: convergent evolution in fungi and bacteria
Source: Open Biol. 2017 Dec 6;7(12):170199. doi: 10.1098/rsob.170199 (PMC5746545; doi:10.1098/rsob.170199)
Supplement: Supplementary Figure S3 high resolution and comments [file rsob170199supp2.pdf]

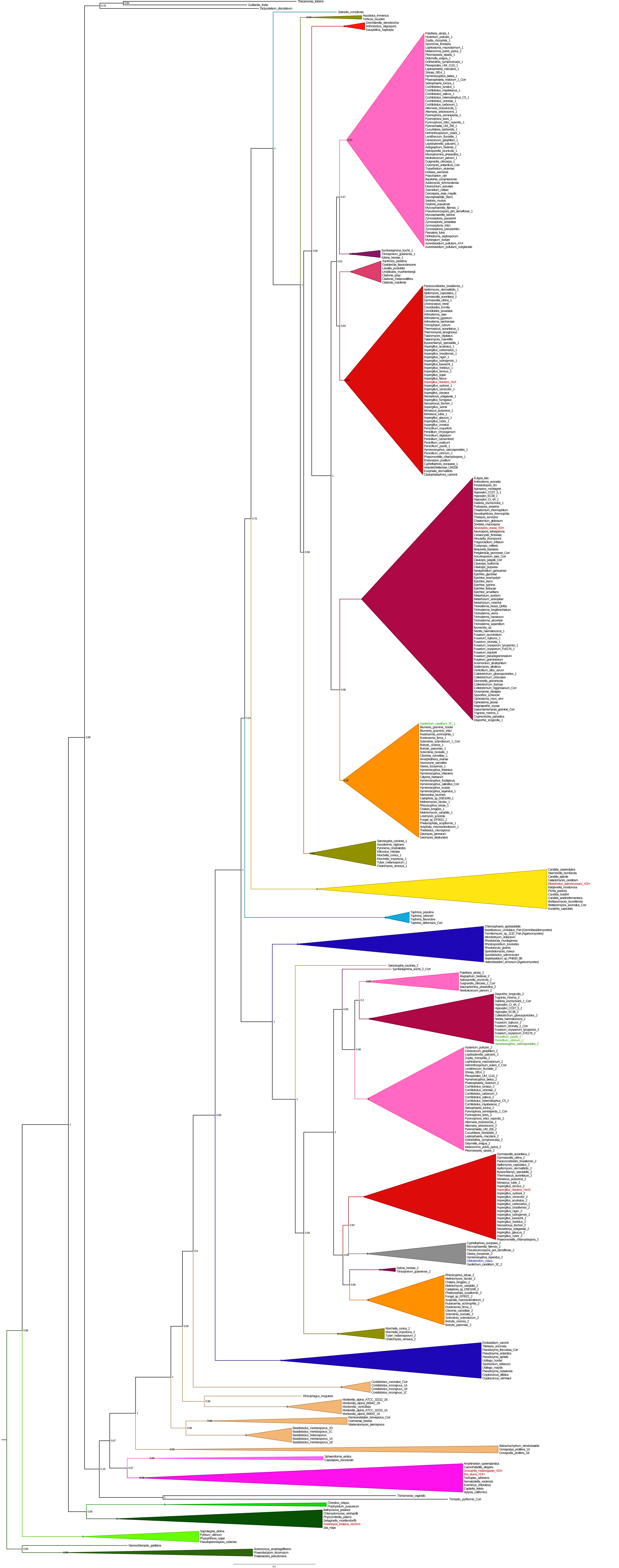

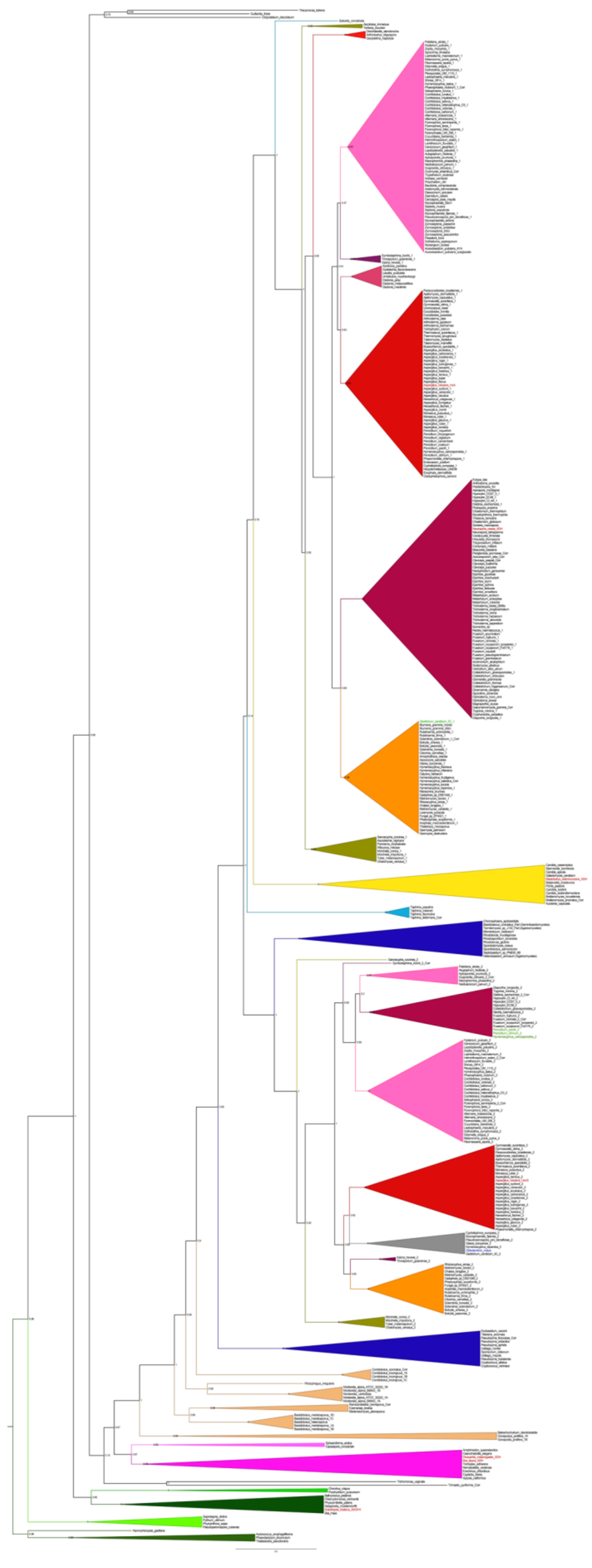

Figure S3. A Maximum Likelihood rooted tree of all available fungal XDH-like enzymes with Eukaryotic out-groups.
High quality readable figure is available as a separate supplementary file uploaded in PDF format.
Colour keys for fungal clades, metazoans and Ichthyosporea (Opisthokonta) are those given in figure 4. Archaeplastida and Stramenopiles, including green algae and plants (Viridiplantae), red algae (Rhodophyta), Oomycetes and Diatoms are at the bottom of the tree, indicated with different shades of green. In black with no additional colouring, representative species from other taxa: *Thecamonas trahens* (Apusozoa, Bikonta), *Guillardia theta* (Cryptophyta, Chromista), *Dictyostelium discoideum* (Amoebozoa, Unikonta), *Trichomonas vaginalis* (Parabasalia, Excavata), *Trimastix pyriformis* (Metamonada, Excavata). In red lettering we indicate proteins where biochemical work is extant (comprising 3D structural work for *Bos taurus* XDH). In green lettering we indicate proteins that are in positions that do not correspond to the taxonomically appropriate position of the organism in question. Searches for genes encoding XDH-like proteins were carried out with both HxA and HxnS of *A. nidulans* as *in silico* probes. All fungal gene models and proteins were deduced manually. The auto-annotated accession numbers were curated as indicated in Supplementary Table S1 (a considerable number of them are miscalled). The proteins were aligned with MAFFT E-INS-i (for sequences with multiple conserved domains and long gaps) with the default parameters. The alignment was curated with BMGE with a Blosum 30 similarity matrix. The Maximum Likelihood phylogeny was carried out with PhyML 3.0 with automatic model selection (LG substitution model selected). Numbers at nodes indicate aLRTs (approximate Likelihood Ratio test values).

Comments on the phylogeny of XDH-like enzymes in the fungi.

The putative XDH from *Galactomyces* (*Geotrichum*) *candidum* (http://www.ncbi.nlm.nih.gov/bioproject/247755) clusters, as expected, with other Saccharomycotina. However there is another sequence at the NCBI database of a genome from a different strain of an identically named organism (http://www.ncbi.nlm.nih.gov/bioproject/243259). This strain shows an HxA orthologue clustering with the HxA orthologues of the Leotiomycetes, and an HxnS orthologue in a mixed clade, clustered (alTR 1.00) with the Leotiomycetes *Oidiodendron maius*, *Hymenoscyphus repandus* and *Glarea lozoyensis* (shown in grey). There seem to be no question that these sequences correspond to two different organisms, the former a genuine member of the Saccharomycotina, the latter of the Leotiomycetes. A few other Purine hydroxylase proteins have an unexpected position: three members of the Eurotiales (in green lettering) which include the only Penicillia to have an HxnS orthologue, cluster with the Hypocreales. The proteins of the basal members of the Taphrinomycotina, *Saitoella complicata* and species of genus *Taphrina* do not cluster together as would be expected. The Basidiomycota are separated into two discrete clades, one (comprising *Ustilago maydis*), which appear as an out-group of all the ascomycete sequences and a second, which clusters with the putative orthologues of HxnS. An interesting positioning is that of the *Oidiodendron maius* (Leotiomycetes, Helotiales) putative XDH. The cognate protein maps within the Pezizomycotina HxnS-like clade. It is one of the two species among all the sequenced Pezizomycotina to have a putative orthologue of HxnS, in the (apparent) absence of an orthologue of HxA. It shows both characteristic sequence insertions in the 2Fe/2S cluster and between the 2Fe/2S domain and the FAD/NAD binding domain. It carries a hydrophobic residue (Val533) where we have Tyr454 and Ile478 in HxA and HxnS, respectively. It has a His (His1124) where HxA has Phe1044 and HxnS has His1069. The ORF is interrupted by four introns, of which the first two are widely conserved among HxnS orthologues, the fourth is conserved in several other HxnS orthologues of the Helotiales, while the third is - within the limits of the genomes available - unique of *O. maius*. There is no question this protein is phylogenetically related to HxnS rather than to HxA. However, uniquely among all putative HxnS structural orthologues, it does not have the sequence FATALH (HxnS: 1064-1069) in its substrate binding site nor does it have FTALF, near universal among HxA Pezizomycotina orthologues (HxA: 1040-1044) and all biochemically characterised XDHs (with one exception, see below); instead it specifies FGALH, (1120-1124). This sequence change is identical to one occurring in the putative XDH of the four *Taphrina* species, while the characterised XDH of *Blastobotrys (Arxula) adeninivorans* [1] has FGATF. Indeed, a Gly residue replaces the Thr in all putative Saccharomycotina HxAs.

While no biochemical work is extant in *O. maius*, we propose that this enzyme is not a Nicotinate hydroxylase but rather a XDH. *O. maius* has all the enzymes of purine breakdown (summarised in [2]), including an orthologue of UaY, the pathway specific regulatory gene characterised in *A. nidulans* [2, 3] and *N. crassa* [4]. On the contrary, it has none of the nicotinate specific clustered genes to be described below. There is no other *hxA/hxnS* paralogue present in the *O. maius* genome. A second species has in its genome an HxnS orthologue in the absence of an HxA orthologue. This is *Rhytidhysteron rufulum* (Dothideomycetes, Hysteriales). At variance with the situation in *O. maius*, the *R. rufulum* gene encodes a typical HxnS enzyme, included in a conserved *hxn* gene cluster.

Other independent, probably genus or even species-specific duplications of XDH-like enzymes occurred in non-dikarya (*Mortierella alpina*, *Conidiobolus incongruus*, *Basidiobolus*

meristosporus, *Gonapodya prolifera*). None of these paralogues carry the diagnostic HxnS sequence FATAL(H). No biochemical work is extant in these fungal species.

Comments on the exon-intron structure of *hxnS* orthologues.

The intron-exon organization is broadly class specific. Eurotiales and Onygenales (Eurotiomycetes) share multiple intron positions with Helotiales (Leotiomycetes) and Xylonomycetaceae (Xylonomycetes), including those of all three *A. nidulans* introns. Most Eurotiales (see below for outstanding exceptions in the *Penicillum* genus) have four conserved introns (in *A. nidulans* the second intron is absent) but only the most 5' intron is present in species of the early divergent class of Pezizomycetes, whose *hxnS* genes usually have five introns. Potentially, this is the only intron that survived the re-functionalisation as it is present in the *hxA* orthologue genes of three of the dozen species of Basidiomycota (*Heterobasidion annosum*, *Microbotryum violaceum*, *Septobasidium sp.* strain PNB30-8B) that occur at the basis of the HxnS branch in the PHI/PHII phylogeny (figure 4 and figure S3). Nevertheless, none of the four *hxnS* introns conserved in the Eurotiales are present in Pleosporales or Botryosphaeriales (usually two introns, one conserved across these orders of Dothideomycetes), Hypocreales and Glomerellales (both Sordariomycetes, 8 and 9 introns, respectively) and Symbiotaphrina (recently assigned to Xylonomycetes, 3 introns, none corresponding to the 5 occupied intron positions found in Xylonomycetaceae).

Intriguing is the exon-intron structure of the *hxnS* gene in some early divergent species in Penicillium, the sister genus of Aspergillus in the family of the Aspergillaceae. The genome sequences of *P. paxilli*, *P. citrinum* and the misnamed species *Hymenoscyphus varicosporoides* all specify 8 introns at exactly the same positions as those in *hxnS* genes of the Nectriaceae family, and share none with Aspergillus *hxnS* (the other sequenced Penicillium species have no *hxnS* orthologue). This exon-intron structure is completely coherent with the phylogeny of fungal Purine Hydroxylase paralogues (figure S4) that shows that the HxnS orthologues in these Penicillium are directly related to those of Hypocreales (Sordariomycetes) rather than to HxnS proteins from other Eurotiales, while the situation for the HxA orthologues is taxonomically completely orthodox: all *Penicillium* species (including *P. citrinum*, *P. paxilli*) clustered with Eurotiales and none with Sordariomycetes. These circumstantial evidence strongly suggests that these exceptional species of *Penicillium* have (re-)acquired an *hxnS* gene from a species of Hypocreales by horizontal gene transfer.

References:

1 Jankowska, D. A., Trautwein-Schult, A., Cordes, A., Hoferichter, P., Klein, C., Bode, R., Baronian, K., Kunze, G. 2013 *Arxula adeninivorans* xanthine oxidoreductase and its application in the production of food with low purine content. *J Appl Microbiol.* **115**, 796-807. (10.1111/jam.12284)
2 Galanopoulou, K., Scazzocchio, C., Galinou, M. E., Liu, W., Borbolis, F., Karachaliou, M., Oestreicher, N., Hatzinikolaou, D. G., Diallinas, G., Amillis, S. 2014 Purine utilization proteins in the Eurotiales: cellular compartmentalization, phylogenetic conservation and divergence. *Fungal genetics and biology : FG & B.* **69**, 96-108. (10.1016/j.fgb.2014.06.005 S1087-1845(14)00108-X [pii])
3 Suarez, T., de Queiroz, M. V., Oestreicher, N., Scazzocchio, C. 1995 The sequence and binding specificity of UaY, the specific regulator of the purine utilization pathway in

Aspergillus nidulans, suggest an evolutionary relationship with the PPR1 protein of *Saccharomyces cerevisiae*. *The EMBO journal*. **14**, 1453-1467.
4 Liu, T. D., Marzluf, G. A. 2004 Characterization of *pco-1*, a newly identified gene which regulates purine catabolism in *Neurospora*. *Current genetics*. **46**, 213-227. (10.1007/s00294-004-0530-8)
