# Supplementary material for: A eukaryotic nicotinate-inducible gene cluster: convergent evolution in fungi and bacteria
Source: Open Biol. 2017 Dec 6;7(12):170199. doi: 10.1098/rsob.170199 (PMC5746545; doi:10.1098/rsob.170199)
Supplement: Supplementary Tables 1-6 [file rsob170199supp3.pdf]

Table S1. Purine- and nicotinate dehydrogenases in the Eukaryota.
These only concern the proteins used for the phylogeny of the purine hydrolases and nicotinate dehydrogenases (PH), figure 4 and figure S3. Corresponding HxA- and HxnS orthologue accession numbers, accessible at NCBI, are listed.

Species<br>(428 strains)	PH-encoding locus	Assigned Locus number (NCBI)	Protein Accession number
**FUNGI – Non-Dikarya**			
Mortierella verticillata	AEVJ01000235.1|:516-5033	MVEG_04668	KFH69864
Mortierella alpina<br>ATCC 32222	ADAG01001033.1|:98453-102900 (gene A)<br>ADAG01001070.1|:218014-222784 (gene B)	NA	
Mortierella alpina<br>B6842	AZCI01001013.1|:76357-80760 (gene A)<br>AZCI01001070.1|:c75761-71087 (gene B)	NA	
Rhizophagus irregularis<br>DAOM 197198w	JARB01005427.1|:15002-20660	GLOINDRAFT_11827	ERZ97180 (partial sequence)
(JGI) Conidiobolus coronatus (also at NCBI)	scaffold_97|78523|83036 (AP) & EST Cluster : Locus7237v1rpkm3.27	(JGI)	
Conidiobolus incongruus	JNEM01008748.1|:9830-14313 (gene A)<br>JNEM01009973.1|:23855-28363 (gene B)<br>JNEM01007870.1|:c12822-8314 (gene C)	NA	
Basidiobolus heterosporus	JNET01030457.1|:c28299-24166	NA	
Basidiobolus meristosporus B9252	JNEO01016617.1|:15438-19574 (gene A)<br>JNEO01012274.1|:c23462-19323 (gene B)<br>JNEO01006932.1|:c54627-50478 (gene C)<br>JNEO01008178.1|:c33456-29330 (gene D)	NA	
(JGI) Coemansia reversa (also at NCBI)	scaffold_25|45856|50052	(JGI)	
(JGI) Martensiomyces pterosporus *	scaffold_37|34086|38369	(JGI) *	
(JGI) Ramicandelaber brevisporus *	scaffold_476|11934|17180<br>includes a 999-nt insert between large direct repeats	(JGI) *	
Batrachochytrium dendrobatidis JAM81	ADAR01000130.1|:40260-46195	BATDEDRAFT_25113	XP_006679022
Gonapodya prolifera	LSZK01000210.1|:c40218-34336 (gene A)<br>LSZK01000087.1|:c48526-42863 (gene B)	M427DRAFT_131958 (gene A)<br>M427DRAFT_51789 (gene B)	KXS19430 (PH1-A)<br><br>KXS20831 (PH1-B)
**FUNGI – Basidiomycota**			
**Ustilaginomycotina**			
Tilletiaria anomala	JMSN01000028.1|:c110580-105791	K437DRAFT_245899	XP_013243943
(JGI) Exobasidium vaccinii *	scaffold_9|220135|224743	(JGI) *	
Ustilago maydis 521	AACP01000110.1|:30755-	UMAG_03264	XP_011389680

	35137		
Sporisorium reilianum	FQ311473.1|388459-392832	sr14258	CBQ73663
Ustilago hordei	CAGI01000169.1|125594-129982	UHOR_05076	CCF51968
Pseudozyma hubeiensis	BAOW01000133.1|c74141-69762	PHSY_006691	XP_012192681
Pseudozyma antarctica	BAFG01000453.1|25766-30136	PAN0_006c295 0	XP_014657078
Pseudozyma aphidis	AWNI01000039.1|:410636-415006	PaG_05999	ETS60008
Pseudozyma flocculos	AOUS01001022.1|:c89545-84928 Includes frameshift	PFL1_06549	XP_007882283 (the C-terminus is not homologous)
**Pucciniomycotina**			
Microbotryum violaceum p1A1 Lamole aka Microbotryum lychnidis-dioicae	AEIJ01000674.1|:21607-26584 CDLV01000009.1|783490-788467	MVLG_06038	KDE03476
(JGI) Sporobolomyces roseus *	scaffold_13|262236|267856 (AP)	(JGI) *	
Sporidiobolus salmonicolor	CENE01000030.1|64145-69992	SPOSA6832_04 433	CEQ42602 (introns were overlooked)
Rhodotorula toruloides	ALAU01000211.1|:63806-68989	RHTO_05260	EMS19097
Rhodotorula glutinis	AEVR01000059.1|:c21640-16463 AEVR02000023.1|:625189-630366	NA	
Rhodotorula mucilaginosa	JWTJ01000147.1|:16672-22067	NA	
(JGI) Chionosphaera apobasidialis *	scaffold_7|364969|369822	(JGI) *	
(JGI) Septobasidium sp. PNB30-8B *	scaffold_20|365285|370933	(JGI) *	
**Agaricomycotina**			
Cryptococcus albidus	LKPZ01000016.1|:c143905-139270	NA	
(JGI) Cryptococcus vishniacii *	scaffold_4|747537|752201	(JGI) *	
Heterobasidion irregulare aka (JGI) Heterobasidion annosum s.l	AEOJ01000007.1|:868231-873947 scaffold_06|868231|873947	HETIRDRAFT _154846	XP_009547290 (exons were overlooked)
Termitomyces sp. J132	JDCH01000751.1|:c472-1 (N-terminus) JDCH01000750.1|:c3691-1 (central) JDCH01000749.1|:c65535-64024 (C-terminus)	J132_08758	KNZ79755 (introns/exons were overlooked)
Basidioascus undulatus	JTLS01000972.1|:c2395-3 (N-terminus) JTLS01000452.1|:c18684-15179 (C-terminus)	NA	
**FUNGI – Ascomycota**			
**Taphrinomycotina**			
(JGI) Saitoella complicata (also at NCBI)	scaffold_1|879434|883597	G7K_4935-t1 (NCBI)	GAO50814 (NCBI)
Taphrina deformans PYCC 5710	CAHR02000232.1|26550-29069 (N-terminus) CAHR02000189.1| c41891-39766 (C-terminus)	TAPDE_00487 0 (N-terminus) TAPDE_00427 9 (C-terminus)	CCG84408 (N-terminus) CCG83939 (C-terminus)

Taphrina deformans JCM 22205	BAVV01000037.1|188095-192270	NA	
Taphrina wiesneri	BAVU01000030.1|203950-208124	NA	
Taphrina flavorubra	BAVW01000029.1|149235-153416	NA	
Taphrina populina	BAVX01000002.1|c50789-46651	NA	
**Saccharomycotina**			
Pichia pastoris DSMZ 70382	CABH01000313.1|c8897-4749	NA	
Pichia pastoris GS115 aka Komagataella phaffii	NC_012964.1|2170100-2174248	PAS_chr2-2_0112	XP_002492094
Kuraishia capsulata	CBUD020000029.1| c49716-45463	KUCA_T00003149001	CDK27172
(JGI) Babjeviella inositovora (also at NCBI)	scaffold_1|977443|981603	(JGI)	
(JGI) Candida arabinofermentans (also at NCBI)	scaffold_1|335985|340238	(JGI)	
(JGI) Candida caseinolytica (also at NCBI)	scaffold_3|1563019|1567173	(JGI)	
Candida boidinii	LMZO01000014.1|:c125385-121141	NA	
Brettanomyces bruxellensis	AZMW01000189.1|:c23017-18752	NA	
Brettanomyces anomalus	LCTY01000001.1|:c483801-479516<br>Including 13 bp duplication	NA	
Blastobotrys adeninivorans	CBZY010000003.1|1014680-1018897	GNLVRS02_ARAD1D12518g	R4ZGN4
Galactomyces candidum CLIB 918	CCBN010000014.1|c400911-396719	BN980_GECA14s01737g	CDO56254
Starmerella bombicola	BBSW01000010.1|c23314-19328	NA	
Candida apicola	LBNK01000001.1|:c1424999-1420899	NA	
**PEZIZOMYCOTINA**			
**Orbiliomycetes**			
Arthrobotrys oligospora	ADOT01000147.1|:c483350-478933	AOL_s00081g325	XP_011123444
Dactylellina haptotyla aka Monacrosporium haptotylum	AQGS01000024.1|:c50805-46433	H072_948	XP_011106940
Drechslerella stenobrocha	ASQI01000063.1|:81295-85680	DRE_00023	EWC48718
**Pezizomycetes**			
Tuber melanosporum	CABJ01001597.1|c75591-71270<br>CABJ01000281.1|c139110-134465 (2)	Locus not annotated<br>Locus not annotated	absent<br>absent (2)
Pyronema omphalodes aka (JGI) Pyronema confluens	CATG01001584.1|c56300-51686	PCON_12424	CCX32154
(JGI) Ascobolus immersus *	scaffold_3|149466|153732 (AP)	(JGI) *	
(JGI) Choiromyces venosus *	scaffold_3|911684|916004<br>scaffold_106|11655|16299 (2)	(JGI) *	
(JGI) Terfezia boudieri *	scaffold_4|1579577|1583929	(JGI) *	

(JGI) Wilcoxina mikolae *	scaffold_8|788009|792435 (AP)	(JGI) *	
(JGI) Ascodesmis nigricans *	scaffold_18|302469|306786 (AP)	(JGI) *	
(JGI) Morchella conica *	scaffold_18|121185|125555 scaffold_64|55926|60640 (AP) (2)	(JGI) *	
(JGI) Morchella importuna *	scaffold_2|586250|590620 (AP) scaffold_44|111510|116194 (2)	(JGI) *	
(JGI) Sarcoscypha coccinea *	scaffold_5|354994|359193 (AP) scaffold_101|113340|117827 (AP) (2)	(JGI) *	
**Lecanoromycetes**			
Cladonia metacorallifera	AXCT02000543.1|:13830-17990	NA	
Cladonia macilenta	AUPP01000438.1|:14544-18704	NA	
(JGI) Cladonia grayi *	scaffold_9|30295|34455	(JGI) *	
Gyalolechia flavorubescens	AUPK01000016.1|:c39184-34925	NA	
(JGI) Xanthoria parietina *	scaffold_7|601906|606148 (AP)	(JGI) *	
Umbilicaria muehlenbergii	JFDN01000109.1|:11228-15506	NA	
Lasallia pustulata	JYIL01001320.1|:11352-15627	NA	
**Xylonomycetes**			
(JGI) Xylona heveae (also at NCBI)	scaffold_11|282831|287152 scaffold_2|3048379|3053309 (AP) (2)	(JGI)	
(JGI) Trinosporium guianense *	scaffold_4|65962|70268 scaffold_77|80761|85664 (AP) (2)	(JGI) *	
(JGI) Symbiotaphrina kochii *	scaffold_6|1001298|1005531 (AP) scaffold_6|1194725|1199457 (AP) (2)	(JGI) *	
**Leotiomycetes**			
Blumeria graminis f. sp. hordei A6	AOLT01012859.1|:c10311-6208	BGHDH14_bgh 06183	CCU77189
Blumeria graminis f. sp. tritici 96224	ANZE01012231.1|:10116-14219	BGT96224_345	EPQ62890
Pseudogymnoascus pannorum var. pannorum M1372	AYKR01000965.1|:3503-7964	V493_06059	KFY23144 (the protein is too long at the N-terminus)
Pseudogymnoascus destructans 20631-21	AEFC01000491.1|:c14789-10399	GMDG_02965	XP_012741374
(JGI) Thelebolus microsporus *	scaffold_5|823033|827281 (AP)	(JGI) *	
Botrytis cinerea T4	ALOC01000611.1|:c46498-42158<br><br>ALOC01000541.1|:c223002-218501 (2)	BofuT4_P06499 0.1 (N-term) BofuT4_P06500 0.1 (C-term) BofuT4_P08161 0.1	CCD43657<br>CCD43658<br>CCD52002 (2)
Sclerotinia sclerotiorum	AAGT01000668.1|:8386-11897 (N-terminus) AAGT01000669.1|1-507 (C-terminus) & (Broad EST) G787P5105FA9.T0 (AP)	SS1G_14350 (N-terminus) SS1G_14351 (C-terminus)	XP_001584737<br>XP_001584738

	(http://www.broadinstitute.org/) AAGT01000385.1|:c12900-8393 (2)	SS1G_08428	XP_001590688 (2) (protein is too short at the N-terminus)
Sclerotinia borealis	AYSA01000353.1|:c11466-7130<br>AYSA01000181.1|:c45080-40566 (2)	SBOR_6728<br><br>SBOR_4076	ESZ92865<br><br>ESZ95547 (intron is wrongly annotated) (2)
Botrytis paeoniae	LBGX01000395.1|:c43557-39218<br>LBGX01000778.1|:c36079-31579 (2)	NA	
Glarea lozoyensis	ALVE01000039.1|:c272536-268251<br>ALVE01000002.1|:421387-426045 (2)	GLAREA_09632<br>GLAREA_08868	XP_008084419<br><br>XP_008076020 (2)
Fungal sp. EF0021	AIET01001355.1|:c278408-274110<br>AIET01001355.1|:849201-853859 (2)	NA	
Marssonina brunnea f. sp. 'multigermtubi' MB_m1	AFXC01000120.1|:33127-37362	MBM_00517	XP_007288406
Oidiodendron maius	JMDP01000167.1|:89042-93667	OIDMADRAFT_171175	KIM95720 (an exon is overlooked)
Geotrichum candidum strain 3C	JMRO01000046.1|:540372-544557<br>JMRO01000106.1|:c212221-207543 (2)	NA	
Amorphotheca resinae	JZSE01000420.1|:c34181-30000<br>JZSE01000223.1|:112121-116302<br>(duplication)	NA	
Ascocoryne sarcoides	AIAA01000122.1|:c142082-137920	NA	
Ciborinia camelliae	LGKQ01001362.1|:c6015-1677<br>LGKQ01000402.1|:25893-30367 (2)	NA	
Rutstroemia echinophila	JWJA01005169.1|:c11070-6763<br>JWJA01000773.1|:2101-6670 (2)	NA	
Poculum sydowianum	JWJB01010317.1|:44342-46812 (N-terminus)<br>JWJB01010820.1|:26-1896 (C-terminus)<br>JWJB01002008.1|:24-4425 (Partial) (2)	NA	
Calycina herbarum	LLEY01000015.1|:c615772-611608	NA	
Hymenoscyphus salicellus	LLCD01003750.1|:1861-5275 (N-terminus)<br>LLCD01004351.1|:c4410-3655 (C-terminus)	NA	
Hymenoscyphus scutula	LKTO01000760.1|:7934-12095	NA	
Hymenoscyphus infarciens	LLCB01000058.1|:c123716-119552	NA	
Hymenoscyphus fructigenus	LKUV01000003.1|:c240395-236231	NA	
Hymenoscyphus fraxineus	LLCC01000020.1|:c20565-16317	NA	

Hymenoscyphus repandus	LLCE01000003.1|:c491479-487313<br>LLCE01000019.1|:c158554-153654 (2)	NA	
Hymenoscyphus laetus CBS 340.76 (Sordariomycetes?)	LLCA01000031.1|:c122585-118392<br>LLCA01000013.1|:c246712-242191 (2)	NA	
Hymenoscyphus varicosporoides CBS 651.66 (Penicillium?)	LLCF01000048.1|:8492-12784<br>LLCF01000007.1|:115792-120768 (2)	NA	
(JGI) Acephala macrosclerotiorum *	scaffold_15|243721|247968<br>scaffold_262|18216|22843 (2)	(JGI) *	
(JGI) Cadophora sp. DSE1049 *	scaffold_37|303465|307676<br>scaffold_2|309814|314467 (2)	(JGI) *	
(JGI) Chalara longipes *	scaffold_6|1017180|1021346 (AP)<br>scaffold_6|178635|183273 (AP) (2)	(JGI) *	
(JGI) Loramyces juncicola *	scaffold_7|1417797|1422435	(JGI) *	
(JGI) Meliniomyces bicolor *	scaffold_15|432795|436964<br>scaffold_15|1921817|1926458 (2)	(JGI) *	
(JGI) Meliniomyces variabilis *	scaffold_4|714981|719148 (AP)<br>scaffold_32|313469|318096 (2)	(JGI) *	
(JGI) Phialocephala scopiformis<br>(also at NCBI)	scaffold_10|952743|956928<br><br>scaffold_10|168639|173443 (AP) (2)	LY89DRAFT_781300<br>LY89DRAFT_706582	KUJ18220<br><br>KUJ17908 (2)
(JGI) Rhizoscyphus ericae *	scaffold_11|722225|726391 (AP)<br>scaffold_49|244301|248951 (2)	(JGI) *	
(JGI) Rutstroemia firma *	scaffold_1|1475225|1479532 (AP)<br>scaffold_2|508085|512630 (2)	(JGI) *	
**Eurotiomycetes**			
Aspergillus nidulans biA1 (reference protein)	X82827.1|585-4817 (Glatigny & Scazzocchio, 1995)	hxA gene (XDH_EMENI) (Glatigny & Scazzocchio, 1995)	CAA58034 / Q12553 xanthine dehydrogenase (purine hydroxylase I)
Aspergillus nidulans FGSC A4	AACD01000098.1|:16884-21116<br>AACD01000170.1|:27760-32118 (2)	AN5613<br><br>AN9178<br>NB. Locus and predicted protein are correct at AspGD	XP_663217<br><br>XP_682447 NCBI WRONG (2)<br>AspGD does not provide accession numbers
Aspergillus terreus	AAJN01000109.1|:112077-116331<br>AAJN01000215.1|:c54600-50204 (2)	ATEG_03959<br><br>ATEG_08616	XP_001213137<br><br>XP_001217202 (2) (homology is lost at the C-terminus)
Aspergillus niger CBS 513.88	NT_166539.1|1331844-1336073<br>NT_166520.1|:290799-295216 (2)	ANI_1_902184<br><br>ANI_1_1056034	XP_001401908<br><br>XP_001390055 (2)
Aspergillus kawachii	BACL01000033.1|114869-	AKAW_01061	GAA82946

	119096<br>BACL01000152.1|:c78579-74163 (2)	AKAW_05047	GAA86933 (2)
Aspergillus sojae	BACA01000963.1|:c204078-199835	NA	
Aspergillus flavus	AAIH02000056.1|:c114946-110692	AFLA_027200	XP_002374230
Aspergillus oryzae RIB40	AP007155.1|:2989828-2994081	AOR_1_195815 4	XP_001820196
Aspergillus fumigatus Af293	AAHF01000005.1|:992326-996586	AFUA_4G1122 0	XP_751707
Neosartorya fischeri	AAKE03000005.1|:c1085018-1080758<br>AAKE03000012.1|:159786-164138 (2)	NFIA_105140<br><br>NFIA_094210	XP_001266923<br><br>XP_001261698 (2)
Neosartorya udagawae	BBXM01000038.1|:471112-475371<br>BBXM01000084.1|:615272-619624 (2)	AUD_2410<br><br>AUD_5206	GAO83450<br><br>GAO86246 (2)
Aspergillus clavatus	AAKD03000029.1|:c247358-243109	ACLA_050310	XP_001271985
(JGI) Aspergillus aculeatus (also at NCBI)	scaffold_2|2697492|2701741 (AP)<br>scaffold_11|1089314|1093677 (2)	(JGI)	
(JGI) Aspergillus carbonarius (also at NCBI)	scaffold_4|1529953|1534188<br>scaffold_11|980305|984734 (AP) (2)	(JGI)	
(JGI) Aspergillus acidus aka A. foetidus aka A. luchuensis (also at NCBI)	scaffold_5|1354508|1358735<br>scaffold_3|3367804|3372220 (AP) (2)	(JGI)	
(JGI) Aspergillus brasiliensis (also at NCBI)	scaffold_7|557633|561862 (AP)<br>scaffold_12|159753|164160 (2)	(JGI)	
(JGI) Aspergillus glaucus CBS 516.65 (also at NCBI)	scaffold_34|67065|71293<br>scaffold_3|1126936|1131415 (2)	(JGI)	
(JGI) Aspergillus sydowii (also at NCBI)	scaffold_4|540227|544475 (AP)<br>scaffold_14|384592|388975 (2)	(JGI)	
(JGI) Aspergillus tubingensis (also at NCBI)	scaffold_7|1026071|1030298 (AP)<br>scaffold_3|303186|307597 (2)	(JGI)	
(JGI) Aspergillus versicolor (also at NCBI)	scaffold_5|1947216|1951462<br>scaffold_16|318700|322980 (AP) (2)	(JGI)	
(JGI) Aspergillus wentii (also at NCBI)	scaffold_1|1624834|1629079	(JGI)	
(JGI) Aspergillus zonatus (also at NCBI)	scaffold_3|1862091|1866377	(JGI)	
(JGI) Eurotium rubrum aka Aspergillus ruber (at NCBI)	scaffold_28|163924|168147<br>scaffold_2|20378|24860 (AP) (2)	(JGI)	
Penicillium chrysogenum Wisconsin 54-1255 aka Penicillium rubens	NW_003020081.2:1498003-1502273	Pc22g06330	XP_002564663
Penicillium oxalicum 114-2	AGIH01000294.1|:c115845-111536	PDE_08434	EPS33472
Penicillium digitatum Pd1	AKCU01000234.1|:c30025-25754	PDIP_33700	XP_014535974
Penicillium roqueforti	CBMR010000309.1:|c28267-	PROQFM164_S	CDM36668

	23997	05g000501	
Penicillium camemberti	CBVV010000316.1|:110005-114280	PCAMFM013_S010g000114	CRL23676
Penicillium paxilli	AOTG01000382.1|:c213578-209295<br>AOTG01000229.1|:c62294-57402 (2)	NA	
Penicillium citrinum	LKUP01000466.1|:8487-12779<br>LKUP01000591.1|:17538-22514 (2)	NA	
Talaromyces stipitatus	ABAS01000006.1|:c1093509-1089259	TSTA_077920	XP_002341817
Talaromyces marneffei	ABAR01000009.1|:530801-535066	PMAA_040820	XP_002151227
Thermomyces lanuginosus	ANHP01000232.1|:96119-100361	NA	
Byssochlamys spectabilis	BAUL01000093.1:c4852-555<br>BAUL01000291.1|16075-20649 (2)	PVAR5_3105<br>PVAR5_8010	GAD94479<br>GAD99299 (2) (exon is overlooked)
(JGI) Thermoascus aurantiacus *	scaffold_12|678656|682937 (AP)<br>scaffold_9|337380|342034 (AP) (2)	(JGI) *	
(JGI) Monascus purpureus *	scaffold_63|30587|34870<br>scaffold_44|18569|23142 (2)	(JGI) *	
(JGI) Monascus ruber (also at NCBI)	scaffold_120|40906|45189<br>scaffold_22|26888|31451 (2)	(JGI)	
Arthroderma benhamiae	ABSU01000026.1|:84719-88976	ARB_02280	XP_003011430
Arthroderma gypseum	ABQE01000233.1|:c92681-88423	MGYG_06870	XP_003170879
Arthroderma otae	ABVF01000212.1|:c147188-142926	MCYG_06415	XP_002844451
Trichophyton rubrum	ACPH01000467.1|:104107-108360	TERG_07188	XP_003232340
Trichophyton verrucosum	ACYE01000253.1|:c29980-25726<br>(Pseudo gene: 2 premature stop codons)	TRV_04973	XP_003020897
Trichophyton equinum	ABWI01000691.1|:c29280-25035	TEQG_03787	EGE04942
Trichophyton tonsurans	ACPI01000629.1|:c32432-28187	TESG_05242	EGD97942
Coccidioides immitis RS	AAEC03000001.1|:c4540799-4536543	CIMG_01495	XP_001247724
Coccidioides posadasii C735 delta SOWgp	ACFW01000049.1|:c1631184-1626931	CPC735_049500	XP_003065725
Uncinocarpus reesii	AAIW01000349.1|:c7717-3483	UREG_05104	XP_002584415
Blastomyces gilchristii SLH14081<br>aka Ajellomyces dermatitidis	ACBU01000519.1|:c192911-188556<br>ACBU01001509.1|:c38550-33943 (2)	BDBG_03499<br><br>BDBG_09398	XP_002626335<br><br>XP_002620218 (2) (inextant introns were annotated)
Histoplasma capsulatum G186AR<br>aka Ajellomyces capsulatus	ABBS02000087.1|:c22632-18273<br>ABBS02000082.1|:c236537-232178<br>(Duplication)<br>ABBS02000272.1|:17083-21682 (2)	HCBG_03511<br><br><br><br><br>HCBG_09167	EEH08222<br><br><br><br><br>EEH02602 (2)

Paracoccidioides brasiliensis Pb03	ABHV02000165.1|:127963-132258<br>ABHV01000435.1|:c128734-124085 (2)	PABG_03125<br><br>PABG_06956	EEH20894<br><br>EEH16869 (2)
(JGI) Gymnascella aurantiaca *	scaffold_19|217052|221418 (AP)<br>scaffold_35|10540|15109 (AP) (2)	(JGI) *	
(JGI) Gymnascella citrina *	scaffold_28|62781|67135<br>scaffold_29|233058|237606 (2)	(JGI) *	
Ascosphaera apis	AARE01006949.1|:528-2520 (N-terminus)<br>AARE01002943.1|:1-2496 (C-terminus)	NA	
Exophiala dermatitidis	AFPA01000145.1|:270526-274608	HMPREF1120_06110	XP_009158553
Herpotrichiellaceae sp. UM238	AMYF01000005.1|:c74264-70140	NA	
Cladophialophora carrionii	AOFF01000008.1|:c658316-654231	G647_01148	XP_008722771
Cyphellophora europaea	AOBU01000050.1|:c187792-183489<br>AOBU01000059.1|:c129366-124948 (2)	HMPREF1541_07421<br>HMPREF1541_08505	XP_008719967<br><br>XP_008721046 (2) (the protein is shorter with 12 AAs at the N-terminus)
Coniosporium apollinis (Dothideomycetes ?)	AJKL01000087.1|:277638-281803	W97_07210	XP_007783379
Endocarpon pusillum Z07020	APWS01000105.1|:37519-41844	EPUS_03718	XP_007805892
Phaeomoniella chlamydospora RR-HG1	JACF01000342.1|:c17229-13019<br>JACF01000099.1|:51840-56436 (2)	NA	
Phaeomoniella chlamydospora UCRPC4	LCWF01000007.1|:c18247-14037<br>LCWF01000106.1|:51742-56338 (2)	UCRPC4_g00293<br>UCRPC4_g04416	KKY28869<br><br>KKY19715 (2)
**Dothideomycetes**			
Leptosphaeria maculans	FP929105.1|c1782909-1778547<br>FP929127.1|:1901889-1906466 (2)	LEMA_P044430.1<br>LEMA_P029440.1	XP_003836907<br><br>XP_003839271 (2)
Pyrenophora teres f. teres	AEEY01000672.1|:c8757-4539<br>AEEY01001409.1|:c56626-52046 (2)	PTT_04424<br>PTT_07497	XP_003296021<br>XP_003297181 (2)
Pyrenophora tritici-repentis	AAXI01000283.1|:336049-340262<br>AAXI01000364.1|:6843-11423 (2)	PTRG_06036<br><br>PTRG_07698	XP_001936369<br><br>XP_001938030 (2)
Pyrenophora seminiperda	ATLS01000374.1|:20973-25206<br>ATLS01000051.1|:c54532-49953 (2)	NA	
Alternaria brassicicola	ACIW01002083.1|:c6103-1869<br>ACIW01000963.1|:46191-50768 (2)	NA	
Alternaria arborescens	AIIC01000053.1|:90370-94580<br>AIIC01000105.1|:c41447-36871 (2)	NA	
Phaeosphaeria nodorum	AAGI01000164.1|:318514-	SNOG_07509	XP_001797843

aka Parastagonospora nodorum	322705<br>Includes frameshift<br>AAGI01000083.1|:96257-100778 (2)	SNOG_04191	XP_001794615 (2)
(JGI) Setosphaeria turcica (also at NCBI)	scaffold_10|771173|775391 (AP)<br>scaffold_14|193421|197997 (2)	SETTUDRAFT _166689<br>SETTUDRAFT _176195	XP_008021540<br><br>XP_008023425 (2)
(JGI) Cochliobolus heterostrophus C5<br>aka (NCBI) Bipolaris maydis	scaffold_2|894394|898657<br><br>scaffold_24|84847|89437 (2)	COCC4DRAFT _183960<br>COCC4DRAFT _45341	XP_014084733<br><br>XP_014073253 (2)
(JGI) Cochliobolus sativus aka (NCBI) Bipolaris sorokiniana	scaffold_1|969116|973381<br><br>scaffold_9|283965|288571 (AP) (2)	COCSADRAFT _105648<br>COCSADRAFT _172265	XP_007694664<br><br>XP_007701174 (2)
Cochliobolus miyabeanus aka (NCBI) Bipolaris oryzae	AMCO01000008.1|:c172179-167915<br>AMCO01000106.1|:63011-67648 (2)	COCMIDRAFT _82012<br>COCMIDRAFT _6414	XP_007683075<br><br>XP_007689217 (2)
Cochliobolus victoriae aka (NCBI) Bipolaris victoriae	AMCY01000020.1|:c224063-219798<br>AMCY01000206.1|:c17540-12922 (2)	COCVIDRAFT _23627<br>COCVIDRAFT _31607	XP_014560158<br>XP_014550728 (2)
Cochliobolus carbonum aka (NCBI) Bipolaris zeicola	AMCN01000037.1|:16058-20323<br>AMCN01000091.1|:c16972-12354 (2)	COCCADRAF T_89627<br>COCCADRAF T_5375	XP_007709785<br><br>XP_007712661 (2)
Pyrenochaeta sp. UM 256	AOUM01000047.1|:c263276-259002<br>AOUM01000070.1|:28064-32595 (2)	NA	
Pleosporales_UM_1110	AJMS01009549.1|:62-4302<br>AJMS01010346.1|:19750-24271 (2)	NA	
Shiraia sp. Slf14	AXZN01000041.1|:63716-67944<br>AXZN01000045.1|:c360961-356434 (2)	NA	
Corynespora cassiicola	JAQF01000469.1|:71108-75395<br>JAQF01000510.1|:88857-93446 (2)	NA	
Helminthosporium solani	AWWW01000929.1|:6138-10368<br>AWWW01001360.1|:10687-15355 (2)	NA	
Hortaea werneckii	AIJO01010339.1|:c12476-8376	NA	
Hysterium pulicare	AJFK01001090.1|:384817-389059<br>AJFK01000232.1|:c34840-30331 (2)	NA	
Rhytidhysteron rufulum	PHI gene is not covered<br>AJFL01001926.1|:c8719-4214 (2)	NA	
Zymoseptoria tritici IPO323<br>aka Mycosphaerella graminicola	ACPE01000001.1|:4432317-4436405	MYCGRDRAF T_53902	XP_003856586
Zymoseptoria passerinii	AFIY01000542.1|:c85462-81374	NA	

Zymoseptoria ardabiliae STIR04_3.3.2	AFIX01000226.1|:c84441-80353	NA	
Zymoseptoria pseudotritici STIR04_5.9.1	AFIT01000355.1|:77694-81782	NA	
(JGI) Baudoinia compniacensis aka (NCBI) Baudoinia panamericana	scaffold_8|382255|386331	BAUCODRAFT_577650	XP_007677710
(JGI) Dothistroma septosporum (also at NCBI)	scaffold_1|703612|707688	DOTSEDRAFT_39924	EME48616
(JGI) Mycosphaerella fijiensis aka (NCBI) Pseudocercospora fijiensis	scaffold_1|3134033|3138388 (AP) AIHZ01000030.1|:c66488-62133 scaffold_2|4952439|4956861 (AP) (2) AIHZ01000140.1|:c185686-181267 (2)	MYCFIDRAFT_48128 MYCFIDRAFT_45300	XP_007920014 XP_007922515 (2)
Pseudocercospora pini-densiflorae	AWYD01001424.1|:12647-16837 AWYD01001792.1|:c21845-17419 (2)	NA	
(JGI) Septoria musiva aka (NCBI) Sphaerulina musiva	scaffold_1|3651001|3655086	SEPMUDRAFT_122866	EMF17508
(JGI) Septoria populicola aka (NCBI) Sphaerulina populicola	scaffold_134|46961|51046 (AP)	NA	
Passalora fulva aka (JGI) Cladosporium fulvum	AMRR01004224.1|:c157026-152902	NA	
Cercospora canescens	ANSM01007968.1|:1-4077 (partial) Lacks 5 residues at the N-terminus	NA	
Mycosphaerella sp. Ston1	AWYF01003274.1|:c74442-70357	NA	
Mycosphaerella laricina	AWYE01001183.1|:c5116-703	NA	
Cladosporium sphaerospermum	PHI gene is found on 4 non-overlapping contigs		
Aureobasidium pullulans AY4	AMCU01000220.1|:c27495-23314	NA	
Guignardia citricarpa aka Phyllosticta citricarpa	AOTE01004287.1|:1006-5157 AOTE01002293.1|:c3937-1 (N-terminus) (2) AOTE01002292.1|:c6905-6298 (C-terminus) (2)	NA	
Neofusicoccum parvum	AORE01001606.1|:75035-79314 AORE01000907.1|:c9850-5340 (2)	UCRNP2_9149 UCRNP2_5272	XP_007588384 XP_007584554 (2)
Macrophomina phaseolina MS6	AHHD01000035.1|:583432-587727 AHHD01000030.1|:93303-97853 (2)	MPH_00876 MPH_00564	EKG21955 EKG22109 (2)
Cryomyces antarcticus	AYQD01001144.1|:c4428-270 Includes 2 frameshifts	NA	
(JGI) Acidomyces	scaffold_74|33407|37489 (AP)	FE78DRAFT_8	KXL48883

richmondensis BFW (also at NCBI)		6749	
(JGI) Aplosporella prunicola *	scaffold_14|248651|252841 scaffold_2|619672|624199 (2)	(JGI) *	
(JGI) Aulographum hederae *	scaffold_10|172303|176508 (AP) scaffold_44|147458|152399 (2)	(JGI) *	
(JGI) Aureobasidium subglaciale EXF-2481 (also at NCBI)	scaffold_4|251391|255572 (AP)	AUEXF2481DRAFT_2656	XP_013346121
(JGI) Botryosphaeria dothidea (also at NCBI)	scaffold_831|195815|200087 (AP) scaffold_402|28207|32803 (2)	(JGI)	
(JGI) Cenococcum geophilum (also at NCBI)	scaffold_23|1399280|1403600 (AP) scaffold_11|224057|228687 (2)	(JGI)	
(JGI) Cercospora zeae-maydis *	scaffold_6|223433|227515	(JGI) *	
(JGI) Cochliobolus lunatus aka Curvularia lunata (at NCBI)	scaffold_18|216235|220480 (AP) scaffold_5|265003|269585 (2)	(JGI)	
(JGI) Cucurbitaria berberidis *	scaffold_1|7139902|7144108 (AP) scaffold_4|485744|490273 (2)	(JGI) *	
(JGI) Didymella exigua *	scaffold_36|111624|115879 (AP) scaffold_30|135581|140123 (2)	(JGI) *	
(JGI) Dissoconium aciculare *	scaffold_2|1439149|1443341	(JGI) *	
(JGI) Dothidotthia symphoricarpi *	scaffold_7|619692|623895 (AP) scaffold_4|1530881|1535403 (AP) (2)	(JGI) *	
(JGI) Lentithecium fluviatile *	scaffold_8|1540548|1544781 scaffold_19|61487|66021 (2)	(JGI) *	
(JGI) Lepidopterella palustris (also at NCBI)	scaffold_10|177880|182186 scaffold_855|602|5299 (AP) (2)	(JGI)	
(JGI) Lophiostoma macrostomum *	scaffold_20|188081|192362 (AP) scaffold_181|9679|14226 (2)	(JGI) *	
(JGI) Melanomma pulvis-pyrius *	scaffold_6|162110|166473 (AP) scaffold_214|51631|56175 (2)	(JGI) *	
(JGI) Myriangium duriaei *	scaffold_4|2011896|2016077 (AP)	(JGI) *	
(JGI) Patellaria atrata *	scaffold_6|123881|128097 scaffold_16|50809|55340 (AP) (2)	(JGI) *	
(JGI) Pleomassaria siparia *	scaffold_3|659655|664055 scaffold_17|636855|641532 (AP) (2)	(JGI) *	
(JGI) Polychaeton citri *	scaffold_2|809474|813577 (AP)	(JGI) *	
(JGI) Sporormia fimetaria *	scaffold_20|305289|309531	(JGI) *	
(JGI) Trypethelium eluteriae *	scaffold_14|396201|400387 (AP)	(JGI) *	
(JGI) Zasmidium cellare *	scaffold_3|1218345|1222508 (AP)	(JGI) *	
(JGI) Zopfia rhizophila *	scaffold_9|2758278|2762514 scaffold_19|1835902|1840427 (2)	(JGI) *	

**Sordariomycetes**			
Thielavia terrestris	CP003014.1|:c1004377-1000144	THITE_2123410	XP_003657567
Myceliophthora thermophila	CP003008.1|:1153538-1157789	MYCTH_2311689	XP_003666737
Podospora anserina	CU638743.1|c1524003-1519799	PODANSg6463	XP_001909427
(JGI) Neurospora discreta (also at NCBI)	scaffold_21|103224|107425	(JGI)	
Neurospora crassa OR74A	AABX02000008.1|:93933-98138	NCU03350	XP_956459
Neurospora tetrasperma FGSC 2508	AFBT01000264.1|:84373-88570	NEUTE1DRAFT_130703	XP_009852458
Sordaria macrospora	CABT02000015.1|:62348-66556	SMAC_04084	XP_003346651
Chaetomium globosum	AAFU01000747.1|:c22381-18151	CHGG_08076	XP_001225732
Chaetomium thermophilum var. thermophilum	ADUW01000094.1|:373236-377660	CTHT_0001520	XP_006690701
Magnaporthe oryzae 70-15	AACU03000115.1|:517654-522108	MGG_12738	XP_003716708
Gaeumannomyces graminis var. tritici	ADBI01000921.1|:12296-16828 Includes a frameshift	GGTG_11192	XP_009227337
Magnaporthe poae	ADBL01000174.1|:c3438-3 (N-terminus) ADBL01000173.1|:c12920-12060 (C-terminus)	MAPG_00752 Locus not annotated	KLU81667 absent
Grosmannia clavigera	ACXQ02000012.1|:c346505-342072	CMQ_6586	XP_014175747 (the N-terminus of the protein is too long)
Verticillium alfalfae aka V. albo-atrum	ABPE01002434.1|:c15152-10989	VDBG_05801	XP_003004688
Verticillium dahliae	ABJE01001128.1|:c1071-4 (N-terminus) Includes 3 frameshifts ABJE01001127.1|:c31897-28874 (C-terminus)	VDAG_07735	XP_009654935
Glomerella graminicola aka Colletotrichum graminicola	ACOD01000254.1|:c63810-59587	GLRG_09943	XP_008098819
(JGI) Acremonium alcalophilum *	scaffold_4|77418|81642	(JGI) *	
(JGI) Cryphonectria parasitica *	scaffold_4|1002846|1007253	(JGI) *	
Trichoderma atroviride	ABDG01000131.1|:5459-9732	TRIATDRAFT_141294	XP_013944701
Trichoderma virens	ABDF01000171.1|:c97927-93693	TRIVIDRAFT_40214	XP_013950236
Trichoderma reesei QM6a	AAIL02000034.1|:869355-873599	TRIREDRAFT_78797	XP_006966038
Cordyceps militaris	AEVU01000504.1|:c126382-122136	CCM_08980	XP_006674178
Fusarium verticillioides aka Gibberella moniliformis	AAIM02000119.1|:588578-592791 Includes a frameshift AAIM02000167.1|:c357-1 (N-terminus) (2) AAIM02000166.1|:c282447-278039 (C-term) (2)	FVEG_09642 Locus not annotated FVEG_12680	EWG50413 (the C-terminus is not homologous) Absent (2) EWG54466 (2)

Fusarium oxysporum f. sp. lycopersici 4287	AAXH01000665.1|:c71151-66938<br><br>AAXH01000944.1|:c28122-23329 (2)	FOXG_11147<br><br>FOXG_15354	KNB11127<br>KNB11128 (identical)<br><br>KNB17256 (2)
Fusarium oxysporum Fo5176	AFQF01002556.1|:c105639-101426<br>AFQF01002934.1|:c47549-42757 (2)	FOXB_09368<br><br>FOXB_11888	EGU80093<br><br>EGU77600 (2)
Fusarium graminearum PH-1<br>aka Gibberella zeae	AACM02000060.1|:45565-49773	FGSG_01561	XP_011317373
Nectria haematococca	ACJF01000006.1|:c756119-751917<br>ACJF01000017.1|:589089-593861 (2)	NECHADRAFT_74201<br>NECHADRAFT_93123	XP_003049835<br><br>XP_003045535 (2)
Metarhizium acridum	ADNI01000757.1|:c12246-7852	MAC_05768	XP_007812108
Metarhizium robertsii<br>aka M. anisopliae ARSEF 23	ADNJ02000001.1|:6191960-6196376	MAA_01730	XP_007817919
Epichloe festucae Fl1	AFRX01000127.1|:46342-50808	NA	
Epichloe festucae E2368	ADFL01000166.1|:c57802-53336	NA	
Epichloe brachyelytri	AFRB01000152.1|:c25173-20683	NA	
Epichloe typhina	AFSE01000297.1|:c6416-1926	NA	
Epichloe amarillans	AFRF01000168.1|:c53010-48544	NA	
Epichloe glyceriae	AFRG01000045.1|:75942-80442	NA	
Neotyphodium gansuense	AFRE01000097.1|:32346-36817	NA	
Periglandula ipomoeae	AFRD01000341.1|:3388-7525<br>Includes two frameshifts	NA	
Claviceps fusiformis	AFRA01000505.1|:2871-7449	NA	
Claviceps paspali	AFRC01001511.1|:c242-1 (N-terminus)<br>AFRC01000532.1|:c18333-14163 (C-terminus)	NA	
Aciculosporium take	AFQZ01002722.1|:646-875 (N-terminus)<br>AFQZ01002052.1|:c4430-74 (C-terminus)	NA	
Claviceps purpurea	CAGA01000046.1|c124305-119726	CPUR_06642	CCE32778
Epichloe elymi	AMDJ01000066.1|:c26941-22451	NA	
Beauveria bassiana	ADAH01000750.1|:c20099-15820	BBA_09514	XP_008602833
Trichoderma longibrachiatum	ANBJ01000092.1|:c177346-173130	NA	
Trichoderma hamatum	ANCB01006209.1|:c3137-4 (N-terminus)<br>ANCB01006208.1|:c2459-1350 (C-terminus)	NA	
Fusarium acuminatum	CBMG010001156.1|:c18880-14674	NA	
Fusarium	CBMF010000113.1|22770-	BN848_001989	CEG02643 (other strain)

pseudograminearum CS5834	26978	0 (other strain)	
Fusarium equiseti	CBMI010004690.1|c8772-4560	NA	
Fusarium fujikuroi B14	ANFV01000109.1|:212834-217048<br>ANFV01000154.1|:258353-263142 (2)	NA	
Fusarium circinata	AYJV01000213.1|:c14123-9906<br>AYJV01002387.1|:c16917-12133 (2)<br>Includes a frameshift	NA	
Hirsutella thompsonii MTCC6686	APKU01000014.1|:82793-87152	NA	
Hirsutella thompsonii MTCC3556	APKB01000178.1|:634-4993	NA	
Tolypocladium inflatum	AOHE01000160.1|:c138459-134125	NA	
Ophiostoma novo-ulmi subsp. novo-ulmi	AMZD01000013.1|:171575-175819	NA	
Ophiostoma piceae	AQHS01000110.1|:c658820-654545	F503_00401	EPE07679
Sporothrix schenckii	AWEQ01000108.1|:20716-25070	HMPREF1624_08655	ERS94944
Daldinia eschscholzii UM1020	AIID01010287.1|:1310-5540<br>AIID01010551.1|:4919-8737 (N-terminus) (2)<br>AIID01010204.1|:1-953 (C-terminus) (2)	NA	
Pestalotiopsis fici	ARNU01000008.1|:180313-184562	PFICI_08825	XP_007835597
Eutypa lata	AORF01002279.1|:7649-11889	UCREL1_7929	XP_007795805
Colletotrichum higginsianum	CACQ02000903.1|4227-7826 (N-terminus)<br>CACQ02001357.1|86-1198 (C-terminus)	CH063_05924 (N-term)<br>CH063_06870 (C-term)	CCF33816 (N-term)<br><br>CCF34999 (C-term)
Colletotrichum gloeosporioides Nara gc5	ANPB01001438.1|:558-4769<br>ANPB01003480.1|:c31721-26700 (2)	CGGC5_4347<br>CGGC5_10775 (N-term)<br>CGGC5_10774 (C-term)	XP_007274869<br>XP_007282293 (N-term) (2)<br><br>XP_007282292 (C-term) (2)
Colletotrichum gloeosporioides Cg-14	AMYD01003168.1|:c568-1 (N-terminus)<br>AMYD01002424.1|:1-3577 (C-terminus)<br>AMYD01001480.1|:10148-15155 (2)	Locus not annotated<br>CGLO_11610<br><br>CGLO_07347	absent<br><br>EQB49081<br><br>EQB52978 (almost OK) (2)
Colletotrichum orbiculare	AMCV01007890.1|:c6433-2232	Cob_13466	ENH77218
Diaporthe longicolla	AYRD01005743.1|:c46504-42236<br>AYRD01007500.1|:16503-21162 (2)	NA	
Togninia minima aka (JGI) Phaeoacremonium aleophilum	AORD01000096.1|:c28643-24415<br>AORD01000675.1|:c22549-18042 (2)	UCRPA7_883<br><br>UCRPA7_4126	XP_007911665<br><br>XP_007914875 (2)
Ceratocystis fimbriata	APWK02000097.1|:34115-38646	NA	
Pochonia chlamydosporia aka Metacordyceps	AOSW01007804.1|:c960-8 (N-terminus)	NA	

chlamydosporia	AOSW01007803.1|:c1163-3 (centre) AOSW01007802.1|:c11319-8973 (C-terminus)		
(JGI) Anthostoma avocetta *	scaffold_26|513374|517604	(JGI) *	
(JGI) Apiospora montagnei *	scaffold_128|73959|78209 (AP)	(JGI) *	
(JGI) Glomerella acutata aka Colletotrichum fiorinae (also at NCBI)	scaffold_27|90095|94323 (AP)	(JGI)	
(JGI) Glomerella cingulata aka Colletotrichum gloeosporoides	scaffold_9|1386510|1390721 (AP) scaffold_7|512563|517601 (AP) (2)	(JGI)	
(JGI) Hypoxylon sp. CI-4A (also at NCBI)	scaffold_36|159626|163847 scaffold_65|18438|23298 (AP) (2)	(JGI)	
(JGI) Hypoxylon sp. CO27-5 (also at NCBI)	scaffold_120|76383|80602 (AP) scaffold_173|28821|33659 (AP) (2)	(JGI)	
(JGI) Hypoxylon sp. EC38 (also at NCBI)	scaffold_112|119391|123610 scaffold_19|20052|24890 (AP) (2)	(JGI)	
(JGI) Ilyonectria sp. *	scaffold_8|1633339|1637549	(JGI) *	
(JGI) Sodiomyces alkalinus *	scaffold_12|1114514|1118775 (AP)	(JGI) *	
(JGI) Trichoderma asperellum *	scaffold_8|868478|872740	(JGI) *	
(JGI) Trichoderma harzianum *	scaffold_14|680395|684644	(JGI) *	
**Ichthyosporea (Opisthokonta)**			
Sphaeroforma arctica	AEOD01005612.1|:c21794-11053	SARC_06399	XP_014155170 (the C-terminus shows no holology)
Capsaspora owczarzaki	ACFS02000276.1|:7908-13454	CAOG_005326	KJE94733 (the C-terminus shows no homology)
**Metazoa (Opisthokonta)**			
Amphimedon queenslandica	ND	LOC100631741	XP_011403627
Caenorhabditis elegans	ND	CELE_F55B11	CAB05902
Drosophila melanogaster	ND	Dmel_CG7642	AAF54895 (rosy)
Bos taurus	ND	XDH_BOVIN	P80457
Trichoplax adhaerens	ND	TRIADDRAFT _27111	EDV24053
Nematostella vectensis	ND	NEMVEDRAF T_v1g192610	XP_001625891
Evechinus chloroticus	GAPB01058245.1|:117-4058 (TSA: transcribed RNA sequence)	NA	
Capitella teleta	ND	CAPTEDRAFT _198744	ELU12941
Aplysia californica	ND	LOC101862427	XP_012939897
**Parabasalia, Excavata**			
Trichomonas vaginalis G3	AAHC01000999.1|:11860-	TVAG_433470	XP_001307869

	15783		
**Metamonada, Excavata**			
Trimastix pyriformis (RCP-MX)	GAFH01000714.1|:c4117-36 (TSA: transcribed RNA sequence) Includes frameshift	NA	
**Cryptophyta, Chromista**			
Guillardia theta	AEIE01002225.1|:c130846-124920	GUITHDRAFT_164776	XP_005827081
**Amoebozoa, Unikonta**			
Dictyostelium discoideum AX4	AAFI02000174.1|:22223-26488	DDB_G0291047	XP_635420
**Apusozoa, Bikonta**			
Thecamonas trahens	ADVD01001535.1|:c28003-23797	AMSG_11410	XP_013752713 (the protein is too short at the N-terminus)
**Rhodophyta, Archaeplastida**			
Chondrus crispus	CAKH01001095.1|:21661-25845	CHC_T00006216001	XP_005718689
Porphyridium purpureum	AROW01002701.1|:2482-6783	NA	
**Viridiplantae, Archaeplastida**			
Bathycoccus prasinos	ND	Bathy11g02970	XP_007510146
Chlamydomonas reinhardtii	ND	CHLREDRAFT_117669	EDP03026
Physcomitrella patens	ND	PHYPADRAFT_162514	EDQ74505
Selaginella moellendorffii	ND	SELMODRAFT_83922	EFJ33495
Arabidopsis thaliana	ND	AT4G34890	AEE86434
Zea mays	ND	LOC100384368	XP_008657455
**Oomycetes, Stramenopiles (Heterokonta)**			
Saprolegnia diclina	AIJL01000187.1|:10838-15096	SDRG_01208	XP_008604947
Pythium ultimum	ADOS01000675.1|:c179221-174896	NA	
Phytophthora sojae	AAQY02000076.1|:329675-334024	PHYSODRAFT_295142	XP_009517573
Pseudoperonospora cubensis	AHJF01000177.1|:c11719-7361	NA	
**Ochrophyta, Stramenopiles (Heterokonta)**			
Nannochloropsis gaditana	GAGR01000097.1|:177-4547 (TSA: transcribed RNA sequence)	Naga_100208g3	EWM22353
Aureococcus anophagefferens	ACJI01000839.1|:10653-14825	AURANDRAFT_36810	XP_009034984
Phaeodactylum tricornutum	ABQD01000070.1|:467845-472860	PHATRDRAFT_15968	XP_002184143 (inextant intron was annotated)

Thalassiosira pseudonana	AAFD02000038.1|:335161-340359	THAPSDRAFT_270071	XP_002294840 (inextant introns were annotated)

NB: PH encoding loci were manually deduced for the fungal kingdom
Letters in bold indicate groups of Eukaryotes
Green coloured text: *hxnS* orthologues tagged (2)
Red coloured text: genes are either wrongly annotated or the gene-products are supposedly non-functional. In parenthesis, some explanation thereto is given.
Grey highlighted text: taxonomical classification is dubious
ND: not deduced by us
NA: genome not annotated
The blue star (*****) indicates each of the fungal species for which the use of the JGI genome sequences was explicitly permitted with a view to manually deduce HxA, HxnS and HxnR proteins for comparative purposes (PH phylogeny and Consurf analysis) prior to publication of these genome sequences. For contact details of the Principal Investigators of the genome projects involved, we refer to the Info pages of the corresponding JGI genome portals.

NOTES:
Leotiomycetes (Erysiphales): *Erysiphe necator* (5 strains) and *Erysiphe pisi* have no PH in their genome.
Piedraia hortae (Dothideomycetes; Capnodiales; Piedraiaceae) does not appear to have PHI (HxA) nor PHII (HxnS).

References: **Glatigny, A. & Scazzocchio, C. (1995).** Cloning and molecular characterization of *hxA*, the gene coding for the xanthine dehydrogenase (purine hydroxylase I) of *Aspergillus nidulans*. *J Biol Chem* **270**, 3534-3550.

Table S2. Gain- and loss-of-function alleles of *hxnR* and *hxnS*

hxnR or hxnS alleles	strain name	parental strain	changed nucleotide	changed amino acid
hxnR^{c}100	HZS.354	HZS.248	T751G	Y226D
hxnR^{c}101	HZS.355		T757C	W228R
hxnR^{c}102	HZS.452		C730G	P219A
hxnR^{c}103	HZS.453		A1883C	K603T
hxnR^{c}104	HZS.454		T751G	Y226D
hxnR^{c}105	HZS.455		T751G	Y226D
hxnR^{c}106	HZS.456		T751G	Y226D
hxnR^{c}107	HZS.457		T751G	Y226D
hxnR^{c}108	HZS.458		G1884T	K603N
hxnR^{c}109	HZS.459		T751A	Y226N
hxnR^{c}110	HZS.460		T757C	W228R
*hxnR^{c}150**	HZS.461		G784T, G787C	D237Y,A238P
*hxnR^{c}151**	HZS.462		G1884T	K603N
*hxnR^{c}152**	HZS.463		T751A	Y226N
hxnR^{c}200	HZS.464	HZS.98	T764C	F230S
hxnR^{c}201	HZS.465		T757C	W228R
hxnR^{c}202	HZS.466		T751G	Y226D
hxnR^{c}203	HZS.467		A1882G	K603E
hxnR^{c}300	HZS.468	HZS.418	C731T	P219L
hxnR^{c}301	HZS.469		T757C	W228R
hxnR^{c}302	HZS.470		T764C	F230S
hxnR^{c}303	HZS.471		T757C	W228R
hxnR^{c}304	HZS.472		A1894C	T607P
hxnR^{c}305	HZS.473		T1769C	F565S
hxnR^{c}306	HZS.474		A752C	Y226S
hxnR^{c}307	HZS.475		G758C	W228S
hxnR^{c}308	HZS.476		T791C	F239S
hxnR^{c}309	HZS.477		T1769C	F565S
hxnR^{c}310	HZS.478		T763A	F230I
hxnR^{c}311	HZS.479		A754G	N227D
*hxnR^{c}7***	FGSCA872	FGSC A26	G1884T	K603N
*hxnR^{c}48***	CS51.2		C1990T	R639C
*hxnR2***	CS302	A148	G227A	G76D
*hxnR3***	CS302.2		G1833A	W578STOP
*hxnR80****	HZS.220	TN0 A21	A244G	K82E
*hxnS29***	HZS.113	CS51.10.1	T3668C	F1213S
*hxnS35***	HZS.110	FGSC A872	(182-185)Δ	98STOP
*hxnS41***	HZS.109		G3064T; 3066Δ	1162STOP

Asterisks indicate the applied mutagenesis. * 4-Nitroquinoline N-oxide mutagenesis, ** Methylnitronitrosoguanidine mutagenesis, *** PCR-based mutagenesis. All other alleles were obtained by UV mutagenesis.

Highlighted amino acid changes in the same background color mark missense mutations in one and the same codon.

Table S3. Orthologue *hxnR* loci and accession numbers of corresponding HxnR orthologues.
These only concern the proteins used for the Consurf analysis of the zinc-finger regulator HxnR (figure S4) and the data given are thus not all inclusive.

Species (126 Pezizomycotina)	hxnR gene locus (manually deduced)	Assigned locus number (NCBI)	Assigned Protein Accession number (NCBI)
**Eurotiomycetes**			
Aspergillus nidulans	AACD01000170.1|:23303-25963	AN11197	CBF82382 (the N-terminus is longer with 6 AAs)
(JGI) Aspergillus sydowii (also at NCBI)	scaffold_14|380488|382993	(JGI)	
(JGI) Aspergillus versicolor (also at NCBI)	scaffold_16|324591|327084 (AP)	(JGI)	
Aspergillus oryzae RIB40	AP007164.1|:c2325614-2323089	AOR_1_1570114	XP_003190358
Aspergillus flavus	AAIH02000271.1|:c19461-16936	AFLA_063610	XP_002379105 (at the N-terminus the protein is shorter with 50 AAs)
Aspergillus sojae	BACA01000149.1|:14054-16582	NA	
Aspergillus terreus	AAJN01000215.1|:c59094-56517	ATEG_08618	XP_001217204 (the N-terminus is too long and carries deleterious gaps)
(JGI) Aspergillus aculeatus (also at NCBI)	scaffold_11|1086464|1088927	(JGI)	
(JGI) Aspergillus carbonarius (also at NCBI)	scaffold_11|986537|989113 (AP)	(JGI)	
Aspergillus niger CBS 513.88	NT_166520.1|:282679-285269	ANI_1_1050034	XP_001390050
(JGI) Aspergillus brasiliensis (also at NCBI)	scaffold_12|149966|152584		
Aspergillus kawachii	BACL01000152.1|:c87133-84533	AKAW_05051	GAA86937 (the N-terminus is too short and one intron is overlooked in the coding sequence)
(JGI) Aspergillus acidicus aka A. foetidus aka A. luchuensis (also at NCBI)	scaffold_3|3378193|3380793 (AP)	(JGI)	
(JGI) Aspergillus tubingensis (also at NCBI)	scaffold_3|293413|296013	(JGI)	
(JGI) Aspergillus wentii (also at NCBI)	scaffold_10|94813|97253	(JGI)	
(JGI) Aspergillus glaucus (also at NCBI)	scaffold_3|1123372|1126027	(JGI)	
JGI) Eurotium rubrum aka Aspergillus ruber (at NCBI)	scaffold_2|25866|28521 (AP)	(JGI)	
Penicillium carneum	CBXS010002886.1|4010-6563	NA	
Penicillium roqueforti	CBMR010000287.1|11236-13789	PROQFM164_S04g001025	CDM36144 (the N-terminus is longer with 5 AAs)
Penicillium camemberti	CBVV010000512.1|c46348-43798	PCAMFM013_S022g000208	CRL27528 (the N-terminus is longer with 5 AAs)
Penicillium aurantiogriseum	ALJY01002280.1|:c4202-1667	NA	
Penicillium biforme	CBXO010000164.1|34125-36675	NA	
Penicillium paneum	CBXN010000112.1|184473-187026	NA	
Penicillium fuscoglaucum	CBXP010000195.1|3	NA	

	5162-37712		
Penicillium digitatum Pd1	AKCU01000378.1|:c 73339-70788	PDIP_55780	XP_014532804 (too short at the N-terminus)
Penicillium paxilli	AOTG01000616.1|:3 1558-34092	NA	
Penicillium citrinum	LKUP01000591.1|:17 538-22514	NA	
(JGI) Monascus purpureus *	scaffold_46|125844|1 28757 (AP)	(JGI) *	
(JGI) Monascus ruber (also at NCBI)	scaffold_149|16408|1 9320	(JGI)	
Talaromyces stipitatus ATCC 10500	ABAS01000027.1|:c1 49321-146803	TSTA_050470	XM_002485516
Talaromyces marneffei ATCC 18224	ABAR01000002.1|:1 362859-1365445	PMAA_031780	XP_002144843 (too long at the N-terminus)
(JGI) Talaromyces aculeatus *	scaffold_1|4922711|4 925290	(JGI) *	
Byssochlamys spectabilis	BAUL01000291.1|98 40-12835	PVAR5_8008	GAD99297
(JGI) Thermoascus aurantiacus *	scaffold_9|344642|34 7679 (AP)	(JGI) *	
Blastomyces gilchristii SLH14081 aka Ajellomyces dermatitidis	ACBU01001509.1|:c 45470-42265	BDBG_09400	XP_002620220 (carries a deletion, which results in frameshift)
Histoplasma capsulatum G186AR aka Ajellomyces capsulatus	ABBS02000272.1|:1 0427-13670	HCBG_09164	EEH02599 (carries a large deleterious gap)
Paracoccidioides brasiliensis Pb03	ABHV01000435.1|:c 135719-132432	PABG_12465	KGY14693
(JGI) Gymnascella aurantiaca *	scaffold_35|17908|20 763 (AP) (Pseudo gene)	(JGI) *	
Cyphellophora europaea	AOBU01000059.1|:1 31907-134781	HMPREF1541_0 8507	XP_008722826 (the N-terminus is 30 AA shorter)
Capronia coronata	AMWN01000007.1|: 33338-36527	A1O1_07578	XP_007728445 (at the N-terminus the protein is shorter with 60 AAs)
Capronia epimyces	AMGY01000008.1|:2 0872-24090	A1O3_08403	XP_007738501 (too short at the N-terminus and carries deleterious gap)
Cladophialophora yegresii	AMGW01000004.1|: 1026744-1029591	A1O7_05689	XP_007757887 (at the N-terminus the protein is shorter with 15 AAs)
Phaeomoniella chlamydospora RR-HG1	JACF01000099.1|:c4 8660-45889	NA	
Phaeomoniella chlamydospora UCRPC4	LCWF01000106.1|:c 48562-45791	UCRPC4_g0441 4	KKY19713 (wrongly annotated, not recognizable sequence)
**Dothideomycetes**			
Phaeosphaeria nodorum	AAGI01000083.1|:10 1735-105051	SNOG_04192	XP_001794616 (at the N-terminus the protein is shorter with 100 AAs)
Pyrenochaeta sp. UM 256	AOUM01000070.1|:3 3523-36976	NA	
Pyrenochaeta lycopersici	GAJI01016548.1|:c29 08-83 (TSA: transcribed RNA sequence)	NA	
Pleosporales sp. UM 1110	AJMS01010346.1|:25 001-28253 NB. Partial at edge of	NA	

	contig		
Leptosphaeria maculans	FP929127.1|:1907810-1911512	LEMA_P029460	XP_003839273 (carries deleterious gaps and the protein is shorter with 70 AAs)
Pyrenophora tritici-repentis	AAXI01000364.1|:12305-15633	PTRG_07699	XP_001938031 (carries deleterious gaps and the protein is too short)
Pyrenophora teres f. teres	AEEY01001409.1|:c51169-47837	PTT_07496	XP_003297180 (protein is shorter with 80 AAs)
Pyrenophora seminiperda	ATLS01000051.1|:c49074-45770	NA	
Alternaria brassicicola	ACIW01000963.1|:56805-59911	NA	
Alternaria arborescens	AIIC01000105.1|:c36091-33125	NA	
(JGI) Setosphaeria turcica (also at NCBI)	scaffold_14|199110|202534	(JGI)	
Shiraia sp. Slf14	AXZN01000045.1|:c355891-352787	NA	
Corynespora cassiicola	JAQF01000510.1|:94995-98309	NA	
(JGI) Cucurbitaria berberidis *	scaffold_4|491158|494514	(JGI) *	
(JGI) Didymella exigua *	scaffold_30|129433|132777 (AP)	(JGI) *	
(JGI) Dothidotthia symphoricarpi *	scaffold_4|1553600|1556996 (AP)	(JGI) *	
(JGI) Lentithecium fluviatile *	scaffold_19|66693|69719	(JGI) *	
(JGI) Lophiostoma macrostomum *	scaffold_181|15049|18274	(JGI) *	
(JGI) Melanomma pulvis-pyrius *	scaffold_214|56963|60202	(JGI) *	
(JGI) Pleomassaria siparia *	scaffold_17|632646|636107 (AP)	(JGI) *	
Helminthosporium solani	AWWW01001360.1|:16328-19540<br>NB. Partial at edge of contig	NA	
Hysterium pulicare	AJFK01000232.1|:c29646-26356	NA	
Rhytidhysteron rufulum	AJFL01001926.1|:c3544-206	NA	
Macrophomina phaseolina MS6	AHHD01000030.1|:100300-104036	MPH_00565	EKG22110 85 (the N-terminus is shorter with 85 AAs)
Guignardia citricarpa aka Phyllosticta citricarpa	AOTE01002292.1|:c4704-713	NA	
Neofusicoccum parvum	AORE01000907.1|:c3154-1 (N-terminus)<br>AORE01001369.1|:7-579 (C-terminus)	UCRNP2_5271 (N-terminus)	XP_007584552 (N-terminus)
(JGI) Botryosphaeria dothidea (also at NCBI)	scaffold_402|35000|38696	(JGI)	
(JGI) Aplosporella prunicola *	scaffold_2|624735|628493<br>NB. Partial at edge of contig	(JGI) *	
(JGI) Mycosphaerella fijiensis aka Pseudocercospora fijiensis	scaffold_5|809879|812505	(JGI)	

(at NCBI)			
Pseudocercospora pini-densiflorae	AWYD01000740.1|:c 516816-514199	NA	
Mycosphaerella laricina	AWYE01001109.1|:c 24071-21180	NA	
Mycosphaerella sp. Ston1	AWYF01000450.1|:1 0814-14321	NA	
Cladosporium sphaerospermum	AIIA01013032.1|:c36 14-1084<br>NB. Partial at edge of contig	NA	
(JGI) Dothistroma septosporum<br>(also at NCBI)	scaffold_6|602123|60 4975	(JGI)	
(JGI) Septoria musiva aka Sphaerulina musiva<br>(also at NCBI)	scaffold_6|979027|98 2647 (AP)	(JGI)	
(JGI) Septoria populicola aka Sphaerulina populicola<br>(also at NCBI)	scaffold_10|119993|1 23959 (AP)<br>NB. Undefined areas in penultimate intron	(JGI)	
(JGI) Zasmidium cellare *	scaffold_6|549912|55 2542 (AP)	(JGI) *	
Aureobasidium pullulans AY4	AMCU01000011.1|:c 35282-32442	NA	
(JGI) Aureobasidium pullulans var. subglaciale EXF-2481 (also at NCBI)	scaffold_10|149795|1 52616 (AP)	(JGI)	
(JGI) Cenococcum geophilum (also at NCBI)	scaffold_11|229142|2 32863	(JGI)	
(JGI) Lepidopterella palustris (also at NCBI)	scaffold_77|61204|64 603 (AP)	(JGI)	
(JGI) Myriangium duriaei *	scaffold_14|693037|6 95801 (hxnR1)<br>scaffold_8|1253906|1 256729 (AP) (hxnR2)	(JGI) *	
(JGI) Patellaria atrata *	scaffold_16|57894|61 339	(JGI) *	
(JGI) Zopfia rhizophila *	scaffold_19|1841613| 1844800	(JGI) *	
**Sordariomycetes**			
Fusarium oxysporum Fo5176	AFQF01002414.1|:17 394-20421	FOXB_07972	EGU81522
Fusarium oxysporum f. sp. lycopersici 4287	AAXH01001227.1|:c 8745-5697	FOXG_17380	KNB20316
Fusarium fujikuroi B14	ANFV01000166.1|:c 39590-36504	NA	
Fusarium verticillioides aka Gibberella moniliformis	AAIM02000142.1|:1 28314-131338 (hxnR1)<br>AAIM02000047.1|:c2 11441-208608 (hxnR2)	FVEG_11007<br>FVEG_03388	EWG52212<br>EWG41242 (unhomologous region in the middle of the protein)
Fusarium circinata	AYJV01000420.1|:c9 1047-87962<br>NB. Partial at edge of contig	NA	
Fusarium acuminatum	CBMG010000685.1| 53703-56749	NA	

Nectria haematococca	ACJF01000056.1|:c115648-112600	NECHADRAFT_102108	XP_003040920 (N-terminus sequence is longer with 16 AAs)
Fusarium virguliforme	AEYB01000915.1|:47734-50770	NA	
(JGI) Ilyonectria sp. *	scaffold_4|3041407|3044563	(JGI) *	
Glomerella graminicola aka Colletotrichum graminicola	ACOD01000324.1|:c55773-52579	GLRG_11296	XP_008100172
Colletotrichum orbiculare	AMCV01004979.1|:c255501-252355	Cob_08884	ENH82459 (an intron is overlooked)
Colletotrichum fioriniae	JARH01000269.1|:c346795-343566	CFIO01_01344	XP_007593481
(JGI) Glomerella acutata aka Colletotrichum fiorinae MH 18 (at NCBI)	scaffold_49|221146|224379 (AP)	(JGI)	
Colletotrichum gloeosporioides Cg-14	AMYD01000831.1|:c17493-14325	CGLO_04093	EQB55919 (gene carries long deletion)
(JGI) Glomerella cingulata aka Colletotrichum gloeosporoides	scaffold_2|843861|847029 (AP)	(JGI)	
Grosmannia clavigera	ACXQ02000048.1|:376723-380065	CMQ_5378	XP_014174598 (an annotated intron is inexistent)
Ophiostoma novo-ulmi subsp. novo-ulmi	AMZD01000132.1|:73343-76666	NA	
Ophiostoma piceae	AQHS01000229.1|:128284-131592	F503_04661	EPE04146
Eutypa lata	AORF01001865.1|:c26181-23062	UCREL1_6605	XP_007794499 (only C-terminus is found)
Pestalotiopsis fici	ARNU01000067.1|:c206030-203109	PFICI_03297	XP_007830069
Daldinia eschscholzii UM1020	AIID01007324.1|:c3008-69	NA	
(JGI) Anthostoma avocetta *	scaffold_33|540390|543402 (AP)	(JGI) *	
(JGI) Hypoxylon sp. CI-4A (also at NCBI)	scaffold_65|28475|31363	(JGI)	
(JGI) Hypoxylon sp. CO27-5 (also at NCBI)	scaffold_173|36751|39802	(JGI)	
(JGI) Hypoxylon sp. EC38 (also at NCBI)	scaffold_19|28113|31172	(JGI)	
Diaporthe longicolla MSPL 10-6	AYRD01007761.1|:c16270-13226	NA	
Togninia minima aka Phaeoacremonium aleophilum (JGI)	AORD01000689.1|:c71201-68278	Locus is not recognised	
**Leotiomycetes**			
Glarea lozoyensis	ALVE01000002.1|:428703-431536	GLAREA_08870	XP_008076022
(JGI) Amorphotheca resinae (also at NCBI)	scaffold_11|1036280|1039464 (AP)	(JGI)	
(JGI) Chalara longipes *	scaffold_6|1389484|1392661	(JGI) *	
(JGI) Meliniomyces bicolor *	scaffold_15|875918|879209	(JGI) *	
(JGI) Meliniomyces variabilis *	scaffold_4|1069211|1072404	(JGI) *	
Fungal sp. EF0021	AIET01001355.1|:932028-935093	NA	

**Xylonomycetes**			
(JGI) Xylona heveae (also at NCBI)	scaffold_2|3056269|3059646	(JGI)	
(JGI) Trinosporium guianense *	scaffold_77|88733|92038	(JGI) *	
(JGI) Symbiotaphrina kochii *	scaffold_6|1202438|1205513	(JGI) *	
**Pezizomycetes**			
Tuber melanosporum	CABJ01000281.1|c145729-143254)	GSTUM_00007914001	XP_002839756 (C-terminus is not correct)
(JGI) Choiromyces venosus *	scaffold_106|6103|9595	(JGI) *	
(JGI) Morchella conica *	scaffold_64|63084|65030 (AP)	(JGI) *	
(JGI) Morchella importuna *	scaffold_44|107091|109037	(JGI) *	

NA: genome not annotated
NB: All gene models were manually deduced
Red coloured text: genes are either wrongly annotated or the gene-products are supposedly non-functional. Between parenthesis, some explanation thereto is given.
AA: amino acids
The blue star (*) indicates each of the fungal species for which the use of the JGI genome sequences was explicitly permitted with a view to manually deduce HxA, HxnS and HxnR proteins for comparative purposes (PH phylogeny and Consurf analysis) prior to publication of these genome sequences. For contact details of the Principal Investigators of the genome projects involved, we refer to the Info pages of the corresponding JGI genome portals.

Table S4. The loci of *hxnS* and/or *hxnR* genes in species in six classes of Pezizomycotina. Note that these two genes do not occur in currently genome-sequenced species in the Orbiliomycetes and Lecanoromycetes classes. This table is not all inclusive.

Code	species	*hxnS* gene locus	*hxnR* gene locus
	**Eurotiomycetes**		
L	Aspergillus nidulans	AACD01000170.1|:27760-32118	AACD01000170.1|:23303-25963
L	Aspergillus terreus	AAJN01000215.1|:c54600-50204	AAJN01000215.1|:c59094-56517
L	Aspergillus kawachii	BACL01000152.1|:c78579-74163	BACL01000152.1|:c87133-84533
L	Aspergillus niger CBS 513.88	NT_166520.1|:290799-295216	NT_166520.1|:282679-285269
R	Aspergillus flavus	-	AAIH02000271.1|:c19461-16936
R	Aspergillus oryzae RIB40	-	AP007164.1|:c2325614-2323089
R	Aspergillus sojae	-	BACA01000149.1|:14054-16582
S	Neosartorya udagawae	BBXM01000084.1|:615272-619624	-
S	Neosartorya fischeri	AAKE03000012.1|:159786-164138	-
L	(JGI) Aspergillus carbonarius	scaffold_11|980305|984734 (AP)	scaffold_11|986537|989113 (AP)
L	(JGI) Aspergillus aculeatus	scaffold_11|1089314|1093677	scaffold_11|1086464|1088927
L	(JGI) Aspergillus acidicus aka Aspergillus foetidus aka Aspergillus luchuensis	scaffold_3|3367804|3372220 (AP)	scaffold_3|3378193|3380793 (AP)
L	(JGI) Aspergillus brasiliensis	scaffold_12|159753|164160	scaffold_12|149966|152584
L	(JGI) Aspergillus glaucus	scaffold_3|1126936|1131415	scaffold_3|1123372|1126027
L	(JGI) Aspergillus sydowii	scaffold_14|384592|388975	scaffold_14|380488|382993
L	(JGI) Aspergillus tubingensis	scaffold_3|303186|307597	scaffold_3|293413|296013
L	(JGI) Aspergillus versicolor	scaffold_16|318700|322980 (AP)	scaffold_16|324591|327084 (AP)
R	(JGI) Aspergillus wentii	-	scaffold_10|94813|97253
L	(JGI) Eurotium rubrum aka Aspergillus ruber	scaffold_2|20378|24860 (AP)	scaffold_2|25866|28521 (AP)
R	Penicillium carneum	-	CBXS010002886.1|4010-6563
R	Penicillium digitatum Pd1	-	AKCU01000378.1|:c73339-70788
R	Penicillium roqueforti	-	CBMR010000287.1|11236-13789
R	Penicillium aurantiogriseum	-	ALJY01002280.1|:c4202-1667
R	Penicillium biforme	-	CBXO010000164.1|34125-36675
R	Penicillium paneum	-	CBXN010000112.1|184473-187026
R	Penicillium camemberti	-	CBVV010000512.1|c46348-43798
R	Penicillium fuscoglaucum	-	CBXP010000195.1|35162-37712
U	Penicillium paxilli	AOTG01000229.1|:c62294-57402	AOTG01000616.1|:31558-34092
U	Penicillium citrinum	LKUP01000591.1|:17538-	LKUP01000289.1|:123220-

		22514	125746
L	Byssochlamys spectabilis	BAUL01000291.1|16075-20649	BAUL01000291.1|9840-12835
L	(JGI) Thermoascus aurantiacus	scaffold_9|337380|342034 (AP)	scaffold_9|344642|347679 (AP)
U	(JGI) Monascus purpureus	scaffold_44|18569|23142	scaffold_46|125844|128757 (AP)
U	(JGI) Monascus ruber	scaffold_22|26888|31451	scaffold_149|16408|19320
R	Talaromyces marneffei	-	ABAR01000002.1|:1362859-1365445
R	Talaromyces stipitatus	-	ABAS01000027.1|:c149321-146803
R	(JGI) Talaromyces aculeatus	-	scaffold_1|4922711|4925290
L	Blastomyces gilchristii SLH14081 aka Ajellomyces dermatitidis	ACBU01001509.1|:c38550-33943	ACBU01001509.1|:c45470-42265
L	Histoplasma capsulatum G186AR aka Ajellomyces capsulatus	ABBS02000272.1|:17083-21682	ABBS02000272.1|:10427-13670
L	Paracoccidioides brasiliensis Pb03	ABHV01000435.1|:c128734-124085	ABHV01000435.1|:c135719-132432
L	(JGI) Gymnascella aurantiaca	scaffold_35|10540|15109 (AP)	scaffold_35|17908|20763 (AP) (Pseudo gene)
S	(JGI) Gymnascella citrina	scaffold_29|233058|237606	-
L	Cyphellophora europaea	AOBU01000059.1|:c129366-124948	AOBU01000059.1|:131907-134781
R	Capronia coronata	-	AMWN01000007.1|:33338-36527
R	Capronia epimyces	-	AMGY01000008.1|:20872-24090
R	Cladophialophora yegresii	-	AMGW01000004.1|:1026744-1029591
L	Phaeomoniella chlamydospora RR-HG1	JACF01000099.1|:51840-56436	JACF01000099.1|:c48660-45889
	**Leotiomycetes**		
L	Glarea lozoyensis	ALVE01000002.1|:421387-426045	ALVE01000002.1|:428703-431536
S	Botrytis cinerea B05.10	AAID01000672.1|:c5950-1449	-
S	Sclerotinia sclerotiorum	AAGT01000385.1|:c12900-8393	-
S	Sclerotinia borealis	AYSA01000181.1|:c45080-40566	-
L	Fungal sp. EF0021	AIET01001355.1|:849201-853859	AIET01001355.1|:932028-935093
R	(JGI) Amorphotheca resinae	-	scaffold_11|1036280|1039464 (AP)
U	(JGI) Chalara longipes	scaffold_6|178635|183273 (AP)	scaffold_6|1389484|1392661
U	(JGI) Meliniomyces bicolor	scaffold_15|1921817|1926458	scaffold_15|875918|879209
U	(JGI) Meliniomyces variabilis	scaffold_32|313469|318096	scaffold_4|1069211|1072404
	**Xylonomycetes**		
L	(JGI) Xylona heveae	scaffold_2|3048379|3053309 (AP)	scaffold_2|3056269|3059646
L	(JGI) Trinosporium guianense	scaffold_77|80761|85664 (AP)	scaffold_77|88733|92038
L	(JGI) Symbiotaphrina kochii	scaffold_6|1194725|1199337 (AP)	scaffold_6|1202438|1205513
	**Pezizomycetes**		
L	Tuber melanosporum	CABJ01000281.1|c139110-134465	CABJ01000281.1|c145729-143254
L	(JGI) Choiromyces venosus	scaffold_106|11655|16299	scaffold_106|6103|9595
L	(JGI) Morchella conica	scaffold_64|55926|60640 (AP)	scaffold_64|63084|65030 (AP)
L	(JGI) Morchella importuna	scaffold_44|111510|116194	scaffold_44|107091|109037

S	(JGI) Sarcoscypha coccinea	scaffold_101|113340|117827 (AP)	scaffold_101|118102|119164 (AP)
	**Sordariomycetes**		
U	Fusarium oxysporum Fo5176	AFQF01002934.1|:c47549-42757	AFQF01002414.1|:17394-20421
U	Fusarium oxysporum f. sp. lycopersici 4287	AAXH01000944.1|:c28122-23329	AAXH01001227.1|:c8745-5697
U	Fusarium fujikuroi B14	ANFV01000154.1|:258353-263142	ANFV01000166.1|:c39590-36504
U	Fusarium verticillioides<br>aka Gibberella moniliformis	gb|AAIM02000166.1|:c282447-278039<br>NB. Partial at edge of contig	AAIM02000142.1|:128314-131338
U	Nectria haematococca	ACJF01000017.1|:589089-593861	ACJF01000056.1|:c115648-112600
U	Fusarium virguliforme	AEYB01001709.1|:4637-9519	AEYB01000915.1|:47734-50770
R	Fusarium acuminatum	-	CBMG010000685.1|53703-56749
U	Fusarium circinata	AYJV01002387.1|:c16917-12133	AYJV01000420.1|:c91047-87962<br>NB. Partial at edge of contig
R	(JGI) Ilyonectria sp.	-	scaffold_4|3041407|3044563
R	Glomerella graminicola<br>aka Colletotrichum graminicola	-	ACOD01000324.1|:c55773-52579
R	Colletotrichum orbiculare	-	AMCV01004979.1|:c255501-252355
R	Colletotrichum fioriniae	-	JARH01000269.1|:c346795-343566
U	Colletotrichum gloeosporioides Cg-14	AMYD01001480.1|:10148-15155	AMYD01000831.1|:c17493-14325
U	(JGI) Glomerella cingulata<br>aka Colletotrichum gloeosporoides	scaffold_7|512563|517601 (AP)	scaffold_2|843861|847029 (AP)
R	Grosmannia clavigera	-	ACXQ02000048.1|:376723-380065
R	Ophiostoma novo-ulmi subsp. novo-ulmi	-	AMZD01000132.1|:73343-76666
R	Ophiostoma piceae	-	AQHS01000229.1|:128284-131592
U	Diaporthe longicolla	AYRD01007500.1|:16503-21162	AYRD01007761.1|:c16270-13226
U	Togninia minima<br>aka Phaeoacremonium aleophilum	AORD01000675.1|:c22549-18042	AORD01000689.1|:c71201-68278
R	Eutypa lata	-	AORF01001865.1|:c26181-23062
R	Pestalotiopsis fici	-	ARNU01000067.1|:c206030-203109
U	Daldinia eschscholzii UM1020	AIID01010551.1|:4919-8737 (N-terminus)<br>AIID01010204.1|:1-953 (C-terminus)	AIID01007324.1|:c3008-69
R	(JGI) Anthostoma avocetta	-	scaffold_33|540390|543402 (AP)
L	(JGI) Hypoxylon sp. CI-4A	scaffold_65|18438|23298 (AP)	scaffold_65|28475|31363
L	(JGI) Hypoxylon sp. CO27-5	scaffold_173|28821|33659 (AP)	scaffold_173|36751|39802
L	(JGI) Hypoxylon sp. EC38	scaffold_19|20052|24890 (AP)	scaffold_19|28113|31172
	**Dothideomycetes**		
L	Phaeosphaeria nodorum	AAGI01000083.1|:96257-100778	AAGI01000083.1|:101735-105051
L	Pyrenochaeta sp. UM 256	AOUM01000070.1|:28064-	AOUM01000070.1|:33523-

		32595	36976
L	Pleosporales sp. UM 1110	AJMS01010346.1|:19750-24271	AJMS01010346.1|:25001-28253<br>NB. Partial at edge of contig
L	Pyrenophora tritici-repentis	AAXI01000364.1|:6843-11423	AAXI01000364.1|:12305-15633
L	Pyrenophora teres f. teres	AEEY01001409.1|:c56626-52046	AEEY01001409.1|:c51169-47837<br>NB. Frameshift in coding seq
L	Pyrenophora seminiperda	ATLS01000051.1|:c54532-49953	ATLS01000051.1|:c49074-45770
L	Alternaria brassicicola	ACIW01000963.1|:46191-50768	ACIW01000963.1|:56805-59911
L	Alternaria arborescens	AIIC01000105.1|:c41447-36871	AIIC01000105.1|:c36091-33125
L	Leptosphaeria maculans	FP929127.1|:1901889-1906466	FP929127.1|:1907810-1911512
L	Shiraia sp. Slf14	AXZN01000045.1|:c360961-356434	AXZN01000045.1|:c355891-352787
L	Corynespora cassiicola	JAQF01000510.1|:88857-93446	JAQF01000510.1|:94995-98309
L	(JGI) Setosphaeria turcica	scaffold_14|193421|197997	scaffold_14|199110|202534
L	(JGI) Cucurbitaria berberidis	scaffold_4|485744|490273	scaffold_4|491158|494514
L	(JGI) Didymella exigua	scaffold_30|135581|140123	scaffold_30|129433|132777 (AP)
L	(JGI) Dothidotthia symphoricarpi	scaffold_4|1530881|1535403 (AP)	scaffold_4|1553600|1556996 (AP)
L	(JGI) Lentithecium fluviatile	scaffold_19|61487|66021	scaffold_19|66693|69719
L	(JGI) Lophiostoma macrostomum	scaffold_181|9679|14226	scaffold_181|15049|18274
L	(JGI) Melanomma pulvis-pyrius	scaffold_214|51631|56175	scaffold_214|56963|60202
L	(JGI) Pleomassaria siparia	scaffold_17|636855|641532 (AP)	scaffold_17|632646|636107 (AP)
L	Helminthosporium solani	AWWW01001360.1|:10687-15355	AWWW01001360.1|:16328-19540<br>NB. Partial at edge of contig
S	(JGI) Cochliobolus heterostrophus C5 aka Bipolaris maydis	scaffold_24|84847|89437	-
S	(JGI) Cochliobolus sativus<br>aka Bipolaris sorokiniana	scaffold_9|283965|288571 (AP)	-
S	Bipolaris oryzae<br>aka Cochliobolus miyabeanus	AMCO01000106.1|:63011-67648	-
S	Bipolaris victoriae<br>aka Cochliobolus victoriae	AMCY01000206.1|:c17540-12922	-
S	Bipolaris zeicola<br>aka Cochliobolus carbonum	AMCN01000091.1|:c16972-12354	-
S	(JGI) Cochliobolus lunatus<br>aka Curvularia lunata	scaffold_5|265003|269585	-
L	Hysterium pulicare	AJFK01000232.1|:c34840-30331	AJFK01000232.1|:c29646-26356
L	Rhytidhysteron rufulum	AJFL01001926.1|:c8719-4214	AJFL01001926.1|:c3544-206
L	Macrophomina phaseolina MS6	AHHD01000030.1|:93303-97853	AHHD01000030.1|:100300-104036
L	Guignardia citricarpa<br>aka Phyllosticta citricarpa	AOTE01002293.1|:c3937-1 (N-terminus)<br>AOTE01002292.1|:c6905-6298 (C-terminus)	AOTE01002292.1|:c4704-713
L	Neofusicoccum parvum	AORE01000907.1|:c9850-5340	AORE01000907.1|:c3154-1 (N-terminus)<br>AORE01001369.1|:7-579 (C-

			terminus)
L	(JGI) Botryosphaeria dothidea	scaffold_402|28207|32803	scaffold_402|35000|38696
L	(JGI) Aplosporella prunicola	scaffold_2|619672|624199	scaffold_2|624735|628493<br>NB. Partial at edge of contig
S	(JGI) Aulographum hederae	scaffold_44|147458|152399	-
U	(JGI) Mycosphaerella fijiensis<br>aka Pseudocercospora fijiensis	scaffold_2|4952439|4956861 (AP)	scaffold_5|809879|812505
U	Pseudocercospora pini-densiflorae<br>aka Mycosphaerella gibsonii	AWYD01001792.1|:c21845-17419	AWYD01000740.1|:c516816-514199
R	Mycosphaerella laricina	-	AWYE01001109.1|:c24071-21180
R	Mycosphaerella sp. Ston1	-	AWYF01000450.1|:10814-14321
R	Cladosporium sphaerospermum	-	AIIA01013032.1|:c3614-1084<br>NB. Partial at edge of contig
R	(JGI) Dothistroma septosporum	-	scaffold_6|602123|604975
R	(JGI) Septoria musiva<br>aka Sphaerulina musiva	-	scaffold_6|979027|982647 (AP)
R	(JGI) Septoria populicola<br>aka Sphaerulina populicola	-	scaffold_10|119993|123959 (AP)<br>NB. Undefined areas in penultimate intron
R	(JGI) Zasmidium cellare	-	scaffold_6|549912|552542 (AP)
R	Aureobasidium pullulans AY4	-	AMCU01000011.1|:c35282-32442
R	(JGI) Aureobasidium pullulans var. subglaciale EXF-2481	-	scaffold_10|149795|152616 (AP)
L	(JGI) Cenococcum geophilum	scaffold_11|224057|228687	scaffold_11|229142|232863
U	(JGI) Lepidopterella palustris	scaffold_855|602|5299 (AP)	scaffold_77|61204|64603 (AP)
R	(JGI) Myriangium duriaei	-	scaffold_14|693037|695801 (hxnR1)<br>scaffold_8|1253906|1256729 (AP) (hxnR2)
L	(JGI) Patellaria atrata	scaffold_16|50809|55340 (AP)	scaffold_16|57894|61339
L	(JGI) Zopfia rhizophila	scaffold_19|1835902|1840427	scaffold_19|1841613|1844800

Codes (left column): L = *hxnS* and *hxnR* genes are present and linked; S = *hxnS* is present-*hxnR* is absent; R = *hxnR* is present-*hxnS* is absent; U = *hxnS* and *hxnR* genes are present but unlinked.

For genomes accessible at the NCBI's WGS database, the accession number(s) of the relevant DNA contigs is (are) given. The genome sequences of *Aspergillus niger* CBS 513.88, *Aspergillus oryzae* RIB40 and *Leptosphaeria maculans* JN3 are available from the NCBI's nr/nt database.

(JGI): genome sequences available at JGI; scaffold data are provided for each gene locus. See also the footnotes under Tables S1 and S3.

(AP): the coding strand is antiparallel of the scaffold direction in the JGI genomes.

Notes: *Myriangium duriaei* appears to specify two paralog *hxnR* genes (but no *hxnS*).

Species that have neither *hxnR* nor *hxnS* in their genome sequences are not listed (including *Aspergillus fumigatus*, *Aspergillus clavatus*, *Aspergillus zonatus*, *Penicillium chrysogenum* and *Neurospora crassa* amongst many others).

Table S5. *A. nidulans* strains used in this work. (All strains are *veA1* mutant)

Strain	Genotype	Purpose	Reference
A148	*pabaA1 wA3*	growth test; UV mutagenesis;	provided by Herb Arst
CS51.2	*hxnR^{c}48 biA1*	sequencing	this work
CS51.10.1	*hxnR^{c}7 biA1 pyrG89 pantoB100*	UV mutagenesis	this work
CS1132	*hxA18 biA1*	parental strain in genetic crosses with HZS.98	(Cultrone, *et al.*, 2005)
CS2902	*pyrG89 riboB2pyroA4 biA1*	recipient strain in transformation experiment to obtain *hxnR* deletion	(Yu, *et al.*, 2004)
CS302	*wA4 pabaA1 hxnR2*	mRNA expression analysis; sequencing	this work
CS302.2	*wA4 pabaA1 hxnR3*	sequencing	this work
CS3095	*areA600 biA1 sb43*	RT-PCR	(Kudla, *et al.*, 1990)
FGSCA26	*biA1*	RT-PCR	(Kafer, 1965)
FGSCA872/ CS51	*hxnR^{c}7 biA1*	growth test; enzyme assay; mRNA expression analysis	(Scazzocchio, *et al.*, 1973)
NA0313	*pantoB100 yA2*	RT-PCR; parental strain in genetic crosses	(Robellet, *et al.*, 2010)
TN02 A21	*riboB2 pyroA4 nkuAΔ::argB^{+}*	growth test; recipient strain for transformation; parental strain of HZS.220	(Nayak, *et al.*, 2006)
HZS.98	*pantoB100 pabaA1*	parental strain in genetic crosses with CS1132; recipient strain for UV mutagenesis	this work
HZS.105	*hxAΔ::zeo pyrG89 pantoB100 biA1*	recipient strain in transformation experiment to obtain *hxnS* deletion	this work
HZS.106	*hxnSΔ::zeo hxAΔ::zeo pyrG89 pantoB100 biA1 pyr4 in trans*	protein assay; parental strain in genetic crosses with HZS.122	this work
HZS.109	*hxnS41 hxnR^{c}7 biA1 wA3*	growth test; protein assay	this work
HZS.110	*hxnS35 hxnR^{c}7 biA1 wA3*	growth test; protein	this work

		assay	
HZS.113	*hxnS29 pyrG89 hxnR*^{c}*7 pantoB100 biA1*	growth test; protein assay	this work
HZS.120	*riboB2 pabaA1*	recipient strain in transformation experiment to obtain *hxnS* deletion	(Hamari, *et al.*, 2009)
HZS.122	*riboB2 pabaA1 yA2*	parental strain in genetic crosses with HZS.106	this work
HZS.135	*hxB20 biA1*	growth test; Northern analysis; RT-PCR	provided by S. Amillis
HZS.136	*hxnRΔ::zeo pantoB100*	growth tests; Northern analysis; RT-PCR, protein assay	this work
HZS.145		growth tests; RT-PCR	this work
HZS.216	*xprD1 biA1 pabaA1*	RT-PCR	(Kudla, *et al.*, 1990) provided by Vicky Sophianopoulou
HZS.220	*hxnPΔ::riboB*$^{+}$ *riboB2 hxnR80 pyroA4 nkuAΔ::argB*$^{+}$	growth test; protein assay	this work (*hxnR80* mutation was introduced by transformation of the "uphxnP-riboB^{+}-downhxnP" *hxnP* substitution cassette carrying a PCR generated mutation in the *hxnP* flanking *hxnR* sequence (will be described elsewhere by Bokor, Flipphi, Ámon, Scazzocchio and Hamari) into TN02 A21)
HZS.245	*hxAΔ::zeo riboB2 pantoB100 biA1*	protein assay	this work
HZS.248	*hxAΔ::zeo pantoB100 pabaA1 yA2*	recipient strain for UV and 4-nitroquinoline 1-oxide mutagenesis	this work (obtained by cross of HZS.106 with HZS.122)
HZS.254	*hxnSΔ::zeo biA1 pyr4 in trans*	enzyme assay	this work (obtained by cross of HZS.106 with HZS.122)
HZS.354	*hxAΔ::zeo pantoB100 pabaA1 yA2 hxnR*^{c}*100*	growth test; enzyme assay	this work (obtained from HZS.248 by UV mutagenesis)
HZS.355	*hxAΔ::zeo pantoB100*	growth test; enzyme	this work (obtained from

	pabaA1 yA2 hxnR^{c}101	assay	HZS.248 by UV mutagenesis)
HZS.405	*hxAΔ::zeo hxnR^{c}7 pyroA4 pabaA1 yA2*	control strain in growth tests and protein assays	this work
HZS.418	*hxA18 pantoB100*	recipient strain for UV mutagenesis	this work (obtained by cross of HZS.98 with CS1132)
HZS.452	*hxAΔ::zeo pantoB100 pabaA1 yA2 hxnR^{c}102*	growth test; enzyme assay	this work (obtained from HZS.248 by UV mutagenesis)
HZS.453	*hxAΔ::zeo pantoB100 pabaA1 yA2 hxnR^{c}103*	growth test; enzyme assay	this work (obtained from HZS.248 by UV mutagenesis)
HZS.454	*hxAΔ::zeo pantoB100 pabaA1 yA2 hxnR^{c}104*	growth test; enzyme assay	this work (obtained from HZS.248 by UV mutagenesis)
HZS.455	*hxAΔ::zeo pantoB100 pabaA1 yA2 hxnR^{c}105*	growth test; enzyme assay	this work (obtained from HZS.248 by UV mutagenesis)
HZS.456	*hxAΔ::zeo pantoB100 pabaA1 yA2 hxnR^{c}106*	growth test; enzyme assay	this work (obtained from HZS.248 by UV mutagenesis)
HZS.457	*hxAΔ::zeo pantoB100 pabaA1 yA2 hxnR^{c}107*	growth test; enzyme assay	this work (obtained from HZS.248 by UV mutagenesis)
HZS.458	*hxAΔ::zeo pantoB100 pabaA1 yA2 hxnR^{c}108*	growth test; enzyme assay	this work (obtained from HZS.248 by UV mutagenesis)
HZS.459	*hxAΔ::zeo pantoB100 pabaA1 yA2 hxnR^{c}109*	growth test; enzyme assay	this work (obtained from HZS.248 by UV mutagenesis)
HZS.460	*hxAΔ::zeo pantoB100 pabaA1 yA2 hxnR^{c}110*	growth test; enzyme assay	this work (obtained from HZS.248 by UV mutagenesis)
HZS.461	*hxAΔ::zeo pantoB100 pabaA1 yA2 hxnR^{c}150*	growth test; enzyme assay	this work (obtained from HZS.248 by 4-nitroquinoline 1-oxide mutagenesis)
HZS.462	*hxAΔ::zeo pantoB100 pabaA1 yA2 hxnR^{c}151*	growth test; enzyme assay	this work (obtained from HZS.248 by 4-nitroquinoline 1-oxide mutagenesis)
HZS.463	*hxAΔ::zeo pantoB100 pabaA1 yA2 hxnR^{c}152*	growth test; enzyme assay	this work (obtained from HZS.248 by 4-nitroquinoline 1-oxide mutagenesis)
HZS.464	*pantoB100 pabaA1 hxnR^{c}200*	growth test; enzyme assay	this work (obtained from HZS.98 by UV mutagenesis)

HZS.465	*pantoB100 pabaA1 hxnR^{c}201*	growth test; enzyme assay	this work (obtained from HZS.98 by UV mutagenesis)
HZS.466	*pantoB100 pabaA1 hxnR^{c}202*	growth test; enzyme assay	this work (obtained from HZS.98 by UV mutagenesis)
HZS.467	*pantoB100 pabaA1 hxnR^{c}203*	growth test; enzyme assay	this work (obtained from HZS.98 by UV mutagenesis)
HZS.468	*hxA18 pantoB100 hxnR^{c}300*	growth test; enzyme assay	this work (obtained from HZS.418 by UV mutagenesis)
HZS.469	*hxA18 pantoB100 hxnR^{c}301*	growth test; enzyme assay	this work (obtained from HZS.418 by UV mutagenesis)
HZS.470	*hxA18 pantoB100 hxnR^{c}302*	growth test; enzyme assay	this work (obtained from HZS.418 by UV mutagenesis)
HZS.471	*hxA18 pantoB100 hxnR^{c}303*	growth test; enzyme assay	this work (obtained from HZS.418 by UV mutagenesis)
HZS.472	*hxA18 pantoB100 hxnR^{c}304*	growth test; enzyme assay	this work (obtained from HZS.418 by UV mutagenesis)
HZS.473	*hxA18 pantoB100 hxnR^{c}305*	growth test; enzyme assay	this work (obtained from HZS.418 by UV mutagenesis)
HZS.474	*hxA18 pantoB100 hxnR^{c}306*	growth test; enzyme assay	this work (obtained from HZS.418 by UV mutagenesis)
HZS.475	*hxA18 pantoB100 hxnR^{c}307*	growth test; enzyme assay	this work (obtained from HZS.418 by UV mutagenesis)
HZS.476	*hxA18 pantoB100 hxnR^{c}308*	growth test; enzyme assay	this work (obtained from HZS.418 by UV mutagenesis)
HZS.477	*hxA18 pantoB100 hxnR^{c}309*	growth test; enzyme assay	this work (obtained from HZS.418 by UV mutagenesis)
HZS.478	*hxA18 pantoB100 hxnR^{c}310*	growth test; enzyme assay	this work (obtained from HZS.418 by UV mutagenesis)
HZS.479	*hxA18 pantoB100 hxnR^{c}311*	growth test; enzyme assay	this work (obtained from HZS.418 by UV mutagenesis)
HZS.546	*alX4 biA1*	parental strain for *hxnR* deletion	(Scazzocchio & Darlington, 1968)
HZS.599	*hxnSΔ::pabaA$^+$ pabaA1 riboB2*	growth test; enzyme assay	this work (obtained by transformation of the "ruphxnS-pabaA$^+$-

			rdownhxnS" substitution cassette into HZS.120)

Explanation of mutant alleles, which are not described in the text: *hxA18* is a chain termination mutation at codon 148 of the *hxA* gene encoding Purine hydroxylase I (Glatigny & Scazzocchio, 1995), *hxAΔ::zeo* is a deletion of *hxA* made by gene substitution with *zeo* marker gene (Hamari and Scazzocchio unpublished), *nkuAΔ* is deletion of *nkuA* that is essential for nonhomologous end joining of DNA in double-strand break repair (Nayak, *et al.*, 2006), *sb43* is a non-functional allele of the sulphate transporter SB (Arst, 1968), *veA1* mutation in *velA* results in profuse conidiation regardless of the presence or absence of light (Kafer, 1965), *yA2* is mutation in *yA* resulting yellow conidia (Clutterbuck, 1972) and *pyr4* is gene for orotidine 5'-phosphate carboxylase in *N. crassa* (Buxton & Radford, 1983), which complements *pyrG89* allele of *A. nidulans*. Other gene symbols refer to auxotrophies: *argB2*, arginine; *biA1*, biotin; *pabaA1*, p-aminobenzoic acid; *pantoB100*, pantothenic acid; *pyroA4*, pyridoxine; *pyrG89* uracil or uridine and *riboB2*, riboflavin.

References:

Arst HN, Jr. (1968) Genetic analysis of the first steps of sulphate metabolism in *Aspergillus nidulans*. *Nature* **219**: 268-270.

Buxton FP & Radford A (1983) Cloning of the structural gene for orotidine 5'-phosphate carboxylase of *Neurospora crassa* by expression in *Escherichia coli*. *Mol Gen Genet* **190**: 403-405.

Clutterbuck AJ (1972) Absence of laccase from yellow-spored mutants of *Aspergillus nidulans*. *J Gen Microbiol* **70**: 423-435.

Cultrone A, Scazzocchio C, Rochet M, Montero-Moran G, Drevet C & Fernandez-Martin R (2005) Convergent evolution of hydroxylation mechanisms in the fungal kingdom: molybdenum cofactor-independent hydroxylation of xanthine via alpha-ketoglutarate-dependent dioxygenases. *Mol Microbiol* **57**: 276-290.

Glatigny A & Scazzocchio C (1995) Cloning and molecular characterization of *hxA*, the gene coding for the xanthine dehydrogenase (purine hydroxylase I) of *Aspergillus nidulans*. *J Biol Chem* **270**: 3534-3550.

Hamari Z, Amillis S, Drevet C, Apostolaki A, Vagvolgyi C, Diallinas G & Scazzocchio C (2009) Convergent evolution and orphan genes in the Fur4p-like family and characterization of a general nucleoside transporter in *Aspergillus nidulans*. *Mol Microbiol* **73**: 43-57.

Kafer E (1965) Origins of translocations in *Aspergillus nidulans*. *Genetics* **52**: 217-232.

Kudla B, Caddick MX, Langdon T*, et al.* (1990) The regulatory gene *areA* mediating nitrogen metabolite repression in *Aspergillus nidulans*. Mutations affecting specificity of gene activation alter a loop residue of a putative zinc finger. *EMBO J* **9**: 1355-1364.

Nayak T, Szewczyk E, Oakley CE*, et al.* (2006) A versatile and efficient gene-targeting system for *Aspergillus nidulans*. *Genetics* **172**: 1557-1566.

Robellet X, Oestreicher N, Guitton A & Velot C (2010) Gene silencing of transgenes inserted in the *Aspergillus nidulans alcM* and/or *alcS* loci. *Curr Genet* **56**: 341-348.

Scazzocchio C & Darlington AJ (1968) The induction and repression of the enzymes of purine breakdown in *Aspergillus nidulans*. *Biochim Biophys Acta* **166**: 557-568.

Scazzocchio C, Holl FB & Foguelman AI (1973) The genetic control of molybdoflavoproteins in *Aspergillus nidulans*. Allopurinol-resistant mutants constitutive for xanthine-dehydrogenase. *Eur J Biochem* **36**: 428-445.

Yu JH, Hamari Z, Han KH, Seo JA, Reyes-Dominguez Y & Scazzocchio C (2004) Double-joint PCR: a PCR-based molecular tool for gene manipulations in filamentous fungi. *Fungal Genet Biol* **41**: 973-981.

Table S6. Primers used in this study for qRT-PCR, sequencing, cloning, construction of gene replacement cassettes and obtaining of gene-specific probes.

**qRT-PCR**	
hxnP ReTi frw	5’- tgtacttctttgactgcatgg -3’
hxnP ReTi rev	5’- gagtattcgttgcccttgag -3’
hxnS ReTi frw	5’- gagcatttctatcttgagacga -3’
hxnS ReTi rev	5’- ccattgtgttctgggtactg -3’
hxnT ReTi frw	5’- ctcgaccagtttctacacgac -3’
hxnT ReTi rev	5’- ccgagatgatttcaaggacga -3’
hxnY ReTi frw	5’- gtcattcttcgatcctctcacc -3’
hxnY ReTi rev	5’- ggttgagtttctcgtcttcgt -3’
hxnZ ReTi frw	5’- cgctgtatttcaactttctccc -3’
hxnZ ReTi rev	5’- cagtagttgcggtaggtcag -3’
hxnR ReTi frw	5’- cggcttctgttctactacagg -3’
hxnR ReTi rev	5’- cagtctaggtctggaaagtctc -3’
actin ReTi frw	5’- ggtatcatgatcggtatggg -3’
actin ReTi rev	5’- tatctgagtgtgaggatacca -3’
AN9174 ReTi frw	5’- ttctttgcacattgccaactc -3’
AN9174 ReTi rev	5’- gcattccaatagcgaaagcc -3’
AN9179 ReTi frw	5’- gtgatgaacgagtacaccca -3’
AN9179 ReTi rev	5’- aggaccttcgtatgcttgac -3’
AN8360 ReTi frw	5’- gcctgggaatctgtatggtg -3’
AN8360 ReTi rev	5’- ccgacaattccaaatgtcttctc -3’
AN5650 ReTi frw	5’- atgtctgctgttatctatctgctc -3’
AN5650 ReTi rev	5’- gccaatcctccttaccttcc- 3’
**DNA probes for Northern or Southern analysis**	
hxnS probe frw	5’- tgacactgaggatcttcgca -3’
hxnS probe rev	5’- ggcgatatcactgagatgac -3’
hxnT probe frw	5’- ctttgcgctgcagcaccgtg -3’
hxnT probe rev	5’- gtaggctggcctgctggact -3’
hxnR probe frw	5’- ggtcactgccgttgatatt -3’
hxnR probe rev	5’- atgctctaggcgatggaatga -3’
hxnP probe frw	5’- ccatctcattgcgggtgtag -3’
hxnP probe rev	5’- gactgcatggatcgagtatg -3’
hxnY probe frw	5’- actcttgtccgtacatctcg -3’
hxnY probe rev	5’- cattgatatagtacgggtga -3’
hxnZ probe frw	5’- gtcgatagcctgcaacatcg -3’
hxnZ probe rev	5’- gatatcggctgtccagccac -3’
hxB probe frw	5’- caggtgcttcgtaaatgaagac -3’
hxB probe rev	5’- gaattggcttctccgcaag -3’
actA probe frw	5’- catggaaggtaaggtttctgcc -3’
actA probe rev	5’- cttagaagcacttgcggtgg -3’
***hxnS* deletion**	
hxnS r up frw	5'- gtgtactcgttcatcacgccaaag -3'
hxnS r up rev	5'- cttgctgttggagacgacttgg -3'
hxnS r paba chim frw	5'- *ccaagtcgtctccaacagcaag*gcacatagctattacacgtatgtttgagac -3'
hxnS r paba chim rev	5'- *catcgccacagctcaagttct*gtagtttgcttgaatggctaacgaggcattg -3'
hxnS r down frw	5'- agaacttgagctgtggcgatg -3'
hxnS r down rev	5'- agagatacagaacatgcatttcttccc -3'
hxnS r up nest frw	5'- cagttgaggcatcttgatgtgag -3'
hxnT rev	5'- ctattgagcgaaagggtagtccgtatag -3'
***hxnR* and *hxnS* deletion with Chaveroce's method**	
hxnR/zeo frw	5'- *taatataatcaaagcatctcctagctctatacggagtctccatcaaagca*ggaattctcagtcctgctcc -3'
hxnR/zeo rev	5'- *agcatgaatactacgacacagaatacagatagacagttgatgcaagctac*gacagaattcctgcagcccg -3'
hxnS/zeo frw	5'- *gagtaaagaggggttcgaggggagaaagggagcccatggtttcgactgtc*ggaattctcagtcctgctcc-3'
hxnS/zeo rev	5'- *tcaatataaacctgccaaaggtgccgtcacatctcgcgtcgaaggctcgg*gacagaattcctgcagcccg -3'
**for cloning**	

hxnR XbaI frw	5'- ttttttttctagacagctaatcctgcagtatagactcctc -3'
hxnR KpnI rev	5'- tttttttggtaccctcaatagagcatgaatactacgacac -3'
hxnS cDNA frw2	5'- tttttttgaattcggactgcaccgagaaccatg -3'
hxnS cDNA rev	5'- tttttttgaattctcaggcaacagcaacgaaaaatcc -3'
**gene specific primers, sequencing primers**	
hxnS 5UTR frw	5'- gtgcatacatcacgactgattgg -3'
hxnS frw0	5'- gtgcatacatcacgactgattgg -3'
hxnS frw2	5'- catgcgaagatcctcagtgtcaac -3'
hxnS rev2	5'- ggacgtttgtcacctgagagagg -3'
hxnS rev5	5'- ggacgtttatggaacgttagagg-3'
hxnS rev6	5'- ccatgctgtctcgtctgaaggacgac -3'
hxnS 2k down rev	5’- aggaccttcgtatgcttgac -3’
hxnS 4k up frw	5'- cagagacagcagaatgtagggactgggtc -3'
hxnR downst probe frw	5’- ggtagtccgtatagcccttctctg-3’
hxnR downst probe rev	5’- gtggagattcatggtactgtaacc -3’
hxnR 2F	5'- gcacgcttatcgtctccactg -3'
hxnR 800F	5'- gtatgatgccaatacagtaaagctacc -3'
hxnR 1375F	5'- cttctcccgcttcaatactacatacc -3'
hxnR 1958 F	5'- agagatacagaacatgcatttcttccc -3'
UR frw	5'- gacggacctgtcgagtcttattgtg -3'
UR rev	5'- cagctataccattctccgttcgcac -3'
UR 1F	5'- gacggacctgtcgagtcttattgtg -3'
UR 2F	5'- cccttcttcagagaagatctgagc -3'
UR 3F	5'- acagtaccatgaatctccactccg -3'
UR 4F	5'- gcgctgggcgtagtattccacattcag -3'
UR 5F	5'- cccaactcctctcatcgcatctgaatttcg -3'
UR 6F	5'- gctccaagaacggatcgcccaactc -3'
UR 7F	5'- cacaccaaccaccgaactaatttacc -3'
UR 8F	5'- cggcgtctcccatttcagacatgaacc -3'
UR 9F	5'- gggaatggcgacataccgaaggac -3'
UR 10F	5'- accgattggtcttgtgtatgcagatgac -3'
UR 11F	5'- aaagagtctcgatcggtcccaatagc -3'
UR 12F	5'- cccttcaaccaggtgcttgacgag -3'
UR 13F	5'- gggaggatgcagacctaggtacga -3'
UR 14F	5'- agtattcaatgtgagtttcctgcagg -3'
UR 15F	5'- gagatgtgacggcacctttggcag -3'
UR 1R	5'- gctatacggactaccctttcgctc -3'
hxnS F0	5'- atggacgccctccttccgagatcatcg -3'
hxnS F420	5'- gggaagttccagctatccgcggatgatatcg -3'
hxnS F820	5'- tcgccaaattcgtacccgaactg -3'
hxnS F1227	5'- ggaaacattgccacggcgtctc -3'
hxnS F1627	5'- aggctgtgctcgatatcgttctc -3'
hxnS 2030	5'- gggcgctgtgggatatgttgatcatacttctc -3'
hxnS F2430	5'- gacgggagtatggatgtttgga -3'
hxnS F2830	5'- acatcccgaatgtctggctgcg -3'
hxnS F3230	5'- ccgtgtctacacagacggatcag -3'
hxnS F3636	5'- tttactcagggagtggcatgca -3'
hxnS F4055	5'- ggtaagcgagccgttggtgctagac -3'
hxnS R275	5'- gtgataacatgctttccagatactc -3'

Underlined letters in the primer sequences or in the primer names refer to the restriction sites designed within.

Italic letters at the 5' end refer to the chimeric nature of the primer.
