# Supplementary material for: A eukaryotic nicotinate-inducible gene cluster: convergent evolution in fungi and bacteria
Source: Open Biol. 2017 Dec 6;7(12):170199. doi: 10.1098/rsob.170199 (PMC5746545; doi:10.1098/rsob.170199)
Supplement: Supplementary Materials and Methods [file rsob170199supp4.pdf]

Supplementary Materials and Methods

Identification of *hxnS* and *hxnR*

Identification of an *hxnS*-carrying cosmid of W31:H08 from the *A. nidulans* ordered cosmid library of chromosome VI (pWE15 based genomic library) [1] was identified by Southern blot analysis using the PCR product of "hxnS probe frw" – "hxnS probe rev" primers as hybridisation probe. A 16,703 bp long *hxnS*-positive BglII fragment was subcloned into the BamHI site of the pKS-Bluescript vector (pHZ-hxnS). Transformation of the W31:H08 cosmid and the 16.7 Kb subclone into *hxnS41* and the *hxnR2* loss-of-function mutants (HZS.109 and CS302, respectively) resulted in the full complementation of the mutations. A 6,820 nt PCR product amplified from total DNA by "UR frw" and "UR rev" primers were cloned into SmaI digested pBluescript SK vector and sequenced by using "hxnS 5UTR frw", "hxnS frw0", "hxnS frw2", "hxnS rev2", "hxnS rev5", "hxnS rev6", "UR 1F", "UR 2F", "UR 3F", "UR 4F", "UR 5F", "UR 6F", "UR 7F", "UR 8F", "UR 9F", "UR 10F", "UR 11F", "UR 12F", "UR 13F", "UR 14F" and "UR 15F" primers. The sequenced region carried the 4,359 nt long complete coding sequence of *hxnS*, which was identical with the AN9178 locus in AspGD genome database (GeneBank accession number KY962010). Upstream to the *hxnS* start codon we identified an ORF (to be called *hxnT*) and a 178 nt long 3' end of a Zn-finger transcription factor (identified as *hxnR*). A PCR product amplified from total DNA by "hxnS 4k up frw" and "hxnS 2k down rev" primers carrying a 4,753 nt long region upstream to the start codon of *hxnS*, the *hxnS* ORF and a 2,825 nt long region downstream to the stop codon of *hxnS* was cloned into SmaI digested pBluescript SK plasmid. From this clone *hxnR* was sequenced by using "hxnR 2F", "hxnR 800F", "hxnR 1375F", "hxnR 1958 F" and pBluescript SK specific "T3" and "T7" primers. Primer sequences are listed in Table S6.

Sequence determination of *hxnS* and *hxnR* mutations and *hxnS* cDNA.

Sequence analysis was carried out either on cloned PCR products or purified PCR products. The *hxnR* PCR products from control- and mutant strains (used primers were "hxnR XbaI frw" and "hxnR KpnI rev") were cloned into KpnI/XbaI digested pBluescript SK plasmid prior to sequence analysis. Sequencing was carried out by using the "hxnR 2F", "hxnR 800F" "hxnR 1375F" and "hxnR 1958 F" primers. The *hxnS* cDNA was amplified with the "hxnS cDNA frw2" and "hxnS cDNA rev" primer pairs from cDNA generated from total RNA sample of an *hxnR*$^{+}$ (FGSC A26) strain grown on 1mM acetamide as sole N-source for 8 hours at 37°C and induced with 1 mM 6-OH nicotinic acid for further 2 hours. The PCR product was cloned into EcoRI digested pBluescript SK plasmid and sequenced with "hxnS F0", "hxnS F420", "hxnS F820", "hxnS F1227", "hxnS F1627", "hxnS 2030", "hxnS F2430", "hxnS F2830", "hxnS F3230", "hxnS F3636", "hxnS F4055" and "hxnS R275" primers. The primers used are listed in Table S6. DNA sequencing was done over both strands by the Sanger sequencing service of LGC (http://www.lgcgroup.com) analysing three independent biological samples.

Deletion of the *hxnR* and *hxnS*

Deletion of *hxnR*. *hxnR* deletion was carried out by the Chaveroce's method [2]. The *hxnR* gene on an *hxnR*-carrying cosmid of W31:H08 from the *A. nidulans* ordered cosmid library of chromosome VI was replaced *in vivo* in *E. coli* KS272 by the *zeo* gene using a phage λ Red expressing *Escherichia coli* strain. The transformation marker *zeo* gene was amplified from plasmid pCDA21 [2] using chimeric primers "hxnR/zeo frw" and "hxnR/zeo rev". These

primers are composed, respectively, of 50 nucleotides with identity to both sides of *hxnR* UTR (upstream the ATG and downstream the termination codon) and 20 nucleotides with identity to the *zeo* gene. The plasmid p-hxnR and the PCR product of zeocin marker gene (flanked by *hxnR* UTR specific sequences) were sequentially transformed into *E. coli* KS272 strain, the *in vivo* recombination was triggered by 0.2% L-arabinose in LB medium at 30°C and subsequently, the recombinant p-hxnRΔ::zeo plasmid containing *E. coli* strains were selected on ampicillin/zeocin containing LB medium. The *zeo* gene leads to no phenotype in *A. nidulans* and therefore we developed a direct selection strategy for the *hxnR* deletion. The p-hxnRΔ::zeo plasmid was used (5.4 µg of DNA) to transform *A. nidulans* strain *alX4 biA1* (HZS.546) and *hxnR* deletion strains were isolated from the selective hypoxanthine + acetamide + allopurinol + 100 µM nicotinic acid medium. Strains carrying *alX4*, a chain termination mutation in the gene encoding allantoinase [3], are strongly inhibited by allantoin or any of its precursors, such as hypoxanthine, in the presence on non-repressive nitrogen sources [4]. Strain HZS.546 is strongly inhibited by hypoxanthine on acetamide as sole nitrogen source. This inhibition is clearly seen in a medium with acetamide as sole nitrogen source, containing allopurinol, which inhibits completely PHI (HxA) [5], and 1mM or 100 µM nicotinic acid. On this medium, inhibition of growth depends on the nicotinate-mediated induction of HxnS, which hydroxylates hypoxanthine to xanthine, the latter converted to uric acid by XanA [6], which is further oxidized to allantoin. A deletion of *hxnR* will prevent the induction of *hxnS,* and thus hypoxanthine toxicity. We took advantage of this to select *hxnR* deletion strains.

Out of 42 tested transformants, 9 showed *hxnRΔ* phenotype (i.e. they did not grow on either allopurinol supplemented hypoxanthine-, nicotinic acid and 6-OH nicotinic acid N-sources). Southern blot analysis of EcoRV digested total DNA using a PCR probe that hybridises with the region directly downstream of *hxnR* (generated by PCR using "hxnR downst probe frw" - "hxnR downst probe rev" primers) showed that the desired genetic exchange between *hxnR* locus on chromosome and the *zeo* allele of the recombinant plasmid had occurred in three transformants. The *hxnRΔ::zeo alX4 biA1* strains were crossed with NA0313 and the selected progeny *hxnRΔ::zeo pantoB100* (HZS.136) was used in further experiments. Primer sequences are listed in Table S6.

Deletion of *hxnS*. The *hxnS* gene was deleted by two different methods. One which originated strain HZS.106 was done as above (using "hxnS/zeo frw" and "hxnS/zeo rev" primers and by co-transformation of the p-hxnSΔ::zeo plasmid with Aspergillus autoreplicative, *N. crassa pyr4* carrying plasmid into HZS.105), the second which originated strain HZS.599 was done by the transformation of a gene substitution cassette constructed by the double-joint PCR method [7] as described previously [8]. The *hxnS* deletion was obtained by using a $pabaA^{+}$ substitution cassette. The "A", "B" and "C" components of the cassette were amplified with the "hxnS rup frw" – "hxnS rup rev", "hxnS r paba chim frw" – hxnS r paba chim rev" and "hxnS rdown frw" – "hxnS rdown rev" primer pairs, respectively. The 2,825 bp, 3,905 bp and 2,664 bp long "A", "B" and "C" components were assembled to a 8,264 bp long substitution cassette using "hxnS r up nest frw" and "hxnT rev" primers. The substitution cassette was transformed into HZS.120 and 50 PABA prototroph strains were selected, pre-screened for the deletion by growth test on nicotinic acid and 6-OH nicotinic acid as sole N-sources and checked with Southern blot analysis using the "C" component (*hxnS* downstream flanking region) as the probe on PvuII digested total DNA. Out of 9 putative *hxnSΔ* transformants, 6 carried the desired single copy integration at the *hxnS* locus. Growth ability of the *hxnSΔ* strains was tested while co-segregation of $pabaA^{+}$ marker gene with the *hxnS* deletion in the progeny of genetic crosses (with HZS.223) was verified. Primer sequences are listed in Table S6.

References:

1. Brody H, Griffith J, Cuticchia AJ, Arnold J, Timberlake WE (1991) Chromosome-specific recombinant DNA libraries from the fungus *Aspergillus nidulans*. Nucleic Acids Res 19: 3105-3109.
2. Chaveroche MK, Ghigo JM, d'Enfert C (2000) A rapid method for efficient gene replacement in the filamentous fungus *Aspergillus nidulans*. Nucleic Acids Res 28: E97.
3. Galanopoulou K, Scazzocchio C, Galinou ME, Liu W, Borbolis F, et al. (2014) Purine utilization proteins in the Eurotiales: cellular compartmentalization, phylogenetic conservation and divergence. Fungal Genet Biol 69: 96-108.
4. Darlington AJ, Scazzocchio C (1967) Use of analogues and the substrate-sensitivity of mutants in analysis of purine uptake and breakdown in *Aspergillus nidulans*. J Bacteriol 93: 937-940.
5. Scazzocchio C, Holl FB, Foguelman AI (1973) The genetic control of molybdoflavoproteins in *Aspergillus nidulans*. Allopurinol-resistant mutants constitutive for xanthine-dehydrogenase. Eur J Biochem 36: 428-445.
6. Cultrone A, Scazzocchio C, Rochet M, Montero-Moran G, Drevet C, et al. (2005) Convergent evolution of hydroxylation mechanisms in the fungal kingdom: molybdenum cofactor-independent hydroxylation of xanthine via alpha-ketoglutarate-dependent dioxygenases. Mol Microbiol 57: 276-290.
7. Yu JH, Hamari Z, Han KH, Seo JA, Reyes-Dominguez Y, et al. (2004) Double-joint PCR: a PCR-based molecular tool for gene manipulations in filamentous fungi. Fungal Genet Biol 41: 973-981.
8. Karacsony Z, Gacser A, Vagvolgyi C, Scazzocchio C, Hamari Z (2014) A dually located multi-HMG-box protein of *Aspergillus nidulans* has a crucial role in conidial and ascospore germination. Mol Microbiol 94: 383-402.
